# Supplementary material for: Regulatory network topology and the genetic architecture of gene expression
Source: Cell Genom. 2026 Apr 22;6(6):101219. doi: 10.1016/j.xgen.2026.101219 (PMC13261694; doi:10.1016/j.xgen.2026.101219)
Supplement: Document S1. Figures S1–S19, Table S1, and Methods S1 [file mmc1.pdf]

Cell Genomics, Volume *6*

Supplemental information

Regulatory network topology and the genetic architecture of gene expression

Matthew Aguirre, Jeffrey P. Spence, Guy Sella, and Jonathan K. Pritchard

Supplemental Note

Methods S1: Modeling gene expression on a directed acyclic graph, related to **STAR Methods**.

In this section, we provide additional motivation for the statistical methods and derive some mathematical formulas used in our study.

We model gene expression with a linear structural equation model (SEM) given a set of causal regulatory relationships specified by a directed acyclic graph (DAG) with n genes. We assume that the structure of the DAG is given and the parameters of the SEM are fully specified, and consider relaxations of this assumption later.

To start, consider the expression of a single gene i: suppose the gene harbors q_i independent cis-eQTLs ($q_i > 0$) and is affected by r_i regulators ($r_i \geq 0$), which may or may not be independent. If we measure the expression y_i of this gene in a randomly chosen individual from population, we can write that

$$y_i = \underbrace{\sum_{k=1}^{q_i} x_{ki}\beta_{ki}}_{\text{cis}} + \underbrace{\sum_{j=1}^{r_i} y_j\gamma_{ji}}_{\text{trans}} + \underbrace{s_i}_{\text{noise}}$$

where above, β_{ki} and x_{ki} are respectively the effect of the k'th eQTL for gene i and an indicator variable for its genotype; γ_{ji} and y_j are the effect of the j'th regulator of gene i and its expression; and s_i is non-genetic noise with zero mean and variance σ_i^2, which we assume is uncorrelated across genes.

Throughout this work, we assume that (1) each regulator acts consistently as an activator or repressor and has the same magnitude of effect (i.e., $\gamma_{ji} = p_j\gamma$, with p_j denoting the sign for gene j), and that (2) the variance due to *cis*-acting eQTLs and transcriptional noise are the same for all genes (respectively 1, and σ^2), and that (3) all genotypes and noise terms are independent. The variance for gene i is then

$$Var(y_i) = \underbrace{1}_{\text{cis}} + \underbrace{\gamma^2 \left(\sum_{j=1}^{r_i} Var(y_j) + 2\sum_{j=1}^{r_i}\sum_{j'=1}^{j-1} p_j p_{j'} Cov(y_j, y_{j'}) \right)}_{\text{trans}} + \underbrace{\sigma_i^2}_{\text{noise}}$$

Now consider the expression of all genes in the entire network: if G is the weighted adjacency matrix of the provided DAG for the GRN, with entries γ_{ji} where regulatory relationships exist and zero otherwise, then we can more compactly write

$$y = \underbrace{Bx}_{\text{cis}} + \underbrace{Gy}_{\text{trans}} + \underbrace{s}_{\text{noise}}$$

where B is the matrix of genetic effects from *cis*-eQTLs and x is a vector of their genotypes. We note that the above expression is valid since G is the matrix representation for DAG — this specifies a coherent set of equations for the expression, y_i, of each gene, and guarantees that the matrix $(I - G)$ is invertible. We can rearrange terms in the above to compute the variance of y:

$$\begin{aligned}
(I - G)y &= Bx + s \\
y &= (I - G)^{-1}(Bx + s) \\
Var(y) &= L^\top Var(Bx + s)L, \quad L = (I - G)^{-1} \\
&= L^\top (I + \sigma^2 I)L \\
&= (1 + \sigma^2)L^\top L
\end{aligned}$$

in which the marginal genetic variance of gene i can be read off the diagonal of $L^\top L$: $V_G(y_i) = (L^\top L)_{ii}$. Moreover, this matrix product implies a decomposition of variance across genes in the network: since $(L^\top L)_{ii} = \sum_{j=1}^{n} L_{ji}^2$ then the contribution of each gene j to the variance of gene i is

$$B_{ji}^2 = \frac{L_{ji}^2}{(L^\top L)_{ii}},$$

and the *cis*-acting fraction of genetic variance is correspondingly

$$\frac{V_{\text{cis},i}}{V_{\text{G},i}} = \frac{L_{ii}^2}{(L^\top L)_{ii}}.$$

Furthermore, the equations above show that the genetic and non-genetic covariances have similar forms:

$$\Sigma_G(y) = L^\top L$$
$$\Sigma_E(y) = \sigma^2 L^\top L.$$

This means that any non-zero genetic contribution to variance has a corresponding non-genetic term which is scaled by a factor of σ^2, and so the heritability for all genes in the network is constant: $h_i^2 = 1/(1+\sigma^2)$. This parity also means that decompositions of genetic variance across genes in the network will generally correspond to decompositions of heritability, as long as this scaling factor is consistent across genes; for clarity in the main text, however, we refer to our work with genetic variance as such when using simulations from this model.

Furthermore, the matrix $L = (I-G)^{-1}$ in the above expression is a total effects matrix for the structural equation model, which gives the marginal effect of the expression of gene j on the expression of gene i. More concretely, this equation corresponds to the Taylor expansion

$$L = (I-G)^{-1} = I + G + G^2 + G^3 + \dots$$

which enumerates all paths through the graph — moreover, since G is the adjacency matrix of a DAG, this is convergent since G is nilpotent (in particular, G^n is the zero matrix since G cannot have a path of length n). We use the squared entries of this matrix as the basis of our proxy for scaled *trans*-eQTL effect sizes. If *trans*-eQTL k for gene i has *cis*-effect β_{kj} on gene j, then the squared *trans*-effect is $\beta_{ki}^2 = (\beta_{kj} L_{ji})^2$. In practice, however, these effects are computed relative to the variance in expression of the focal gene (j for the *cis* effect, or i for the *trans* effect). Hence, to compare the relative scales of *cis* and *trans* effect sizes in this model, we use the proportion of variance of gene i explained by gene j,

$$B_{ji}^2 = \frac{L_{ji}^2}{(L^\top L)_{ii}}$$

as a proxy for *trans* effects on gene i from *cis*-eQTLs of gene j. This corresponds to the assumption that *cis* effects $\beta_{kj}, \beta_{k'i}$ (of eQTL k on gene j, and eQTL k' on gene i) are equal.

Supplementary Tables

Algorithm 1 Modular scale-free DAG

Require:

- n: Number of genes (nodes) in the network ($n \geq 3$).
- k: Number of groups in the network ($1 \leq k \leq n$).
- r: Expected number of regulators per gene ($r > 0$).
- m -or- w: Expected fraction of edges within groups ($0 \leq m \leq 1$; $w \geq 0$; $m = w/(w + k - 1)$)
- δ: Out-degree uniformity term ($\delta \geq 0$).

▷ *Temporarily set the top of the graph G to be a cycle on $\lfloor 2r \rfloor$ nodes.* ◁
$G \leftarrow \{(1 \rightarrow 2), (2 \rightarrow 3), \ldots, (\lfloor 2r \rfloor \rightarrow 1)\}$
▷ *Randomly assign each of the n nodes to one of k evenly sized groups.* ◁
group(i) $\leftarrow I_i, \quad I_i \in \{1, 2, \ldots, k\}$

▷ *First simulate the bottom section of the DAG, from genes $\lfloor 2r \rfloor + 1, \ldots, n$.* ◁
for $i \in \{\lfloor 2r \rfloor + 1, \ldots, n\}$ **do**
  ▷ *Pick a Poisson number of regulators for gene i (i.e., its in-degree):* ◁
  in-deg(i) $\sim$ Poisson(r)
  for $j \in \{1, \ldots,$ in-deg(i)$\}$ **do**
    ▷ *Sample the j'th regulator with replacement, weighted by group membership and out-degrees.* ◁
    $j \leftarrow j \in \{1, \ldots, i-1\}, \quad p_{ji} \propto (1 + (w-1)\mathbb{1}(I_i = I_j)) \times$ (out-deg(j) $+ \delta$)
    ▷ *Add the edge (j, i), allowing duplicate edges, to the graph* ◁
    $G \leftarrow (j \rightarrow i)$

▷ *Then replace the temporary edges at the top of the DAG with random structure.* ◁
for $i \in \{1, \ldots, \lfloor 2r \rfloor\}$ **do**
  for $j \in \{1, \ldots, i-1\}$ **do**
    ▷ *These edges exist independently with equal probability.* ◁
    $G \leftarrow (j \rightarrow i)$, with probability $p_{ji} = 2r/(n-1)$

Table S1: Algorithm to generate modular scale-free directed acyclic graphs, related to **Fig. 2**.

Supplementary Figures

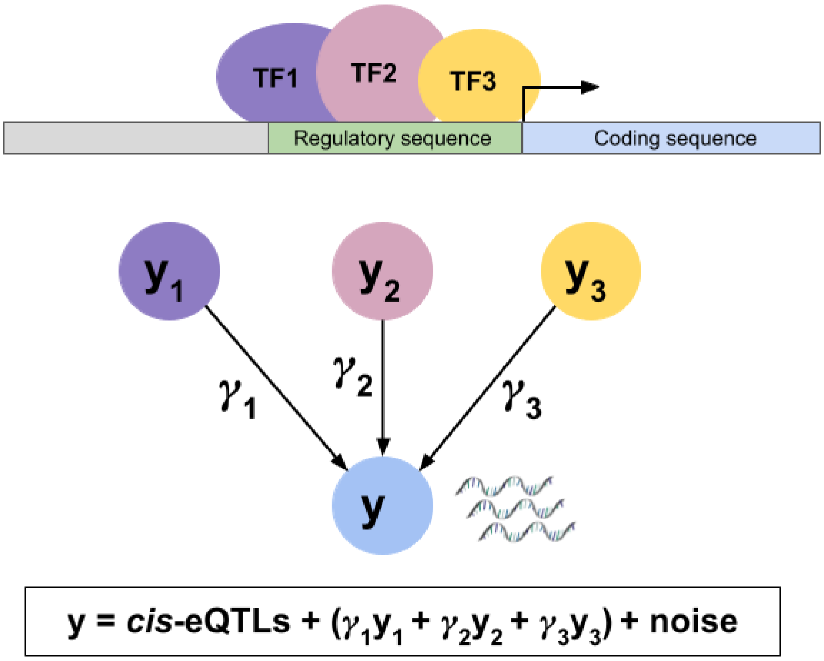

Figure S1: Biological interpretation of linear structural equation model, related to **Fig. 1**. Nodes in the gene regulatory network represent genes. Edges represent regulatory interactions, e.g., between transcription factors and a target gene. Each interaction has strength γ, which corresponds to the (linear) effect of one unit change in expression of the regulator (e.g., y_1) on the expression of the target gene (here, y). Note that the equation in this figure abstract away the effect sizes of *cis*-eQTLs and the nature of endogenous transcriptional noise for the focal gene. Its *trans*-eQTL effects are likewise implicit in the expression in terms of its regulators — they are *cis*-eQTLs for the expression of y_1, y_2, and y_3.

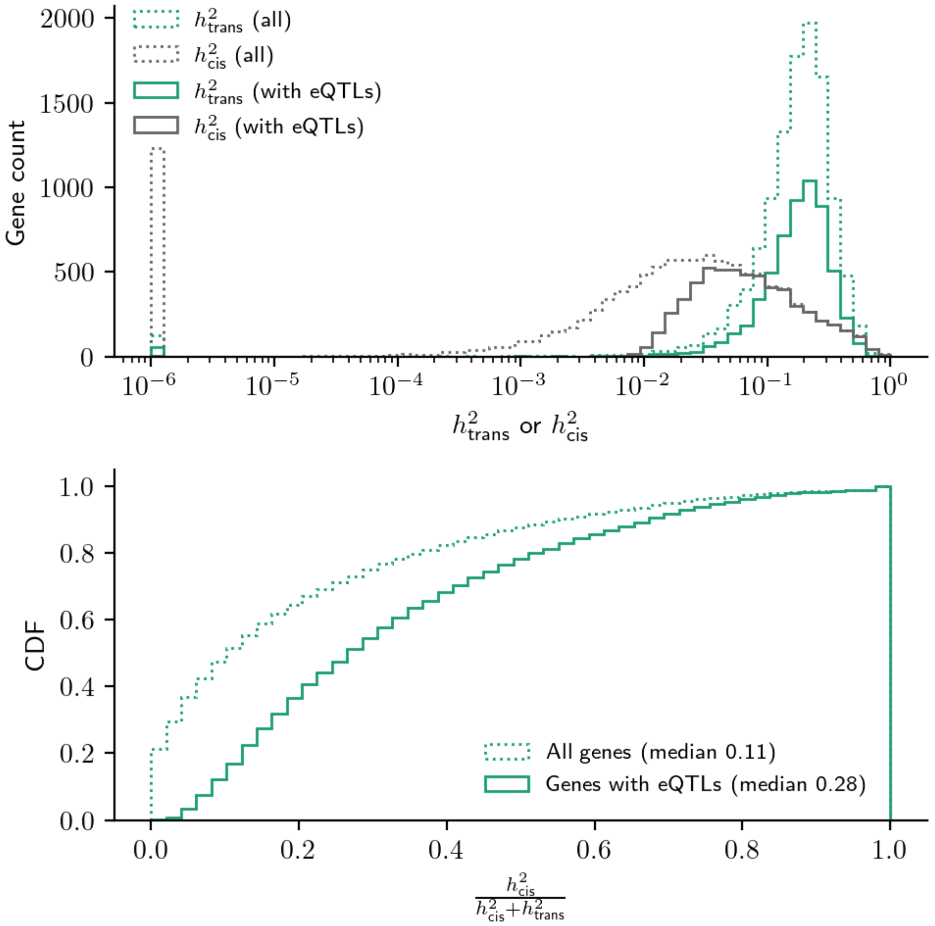

Figure S2: Distribution of *cis* and *trans* heritability for genes with and without eQTLs, related to **Fig. 1**. Data from (Ouwens *et. al.*, 2020)[1] **(A)** Distribution of h^2_{cis} and h^2_{trans} for all $11{,}353$ genes with available data from the study (dotted lines) or for all $5{,}902$ genes in the analysis subset from **Fig. 1**, with a detected eQTL (solid lines). **(B)** Distribution of *cis*-heritability fraction from these same gene sets.

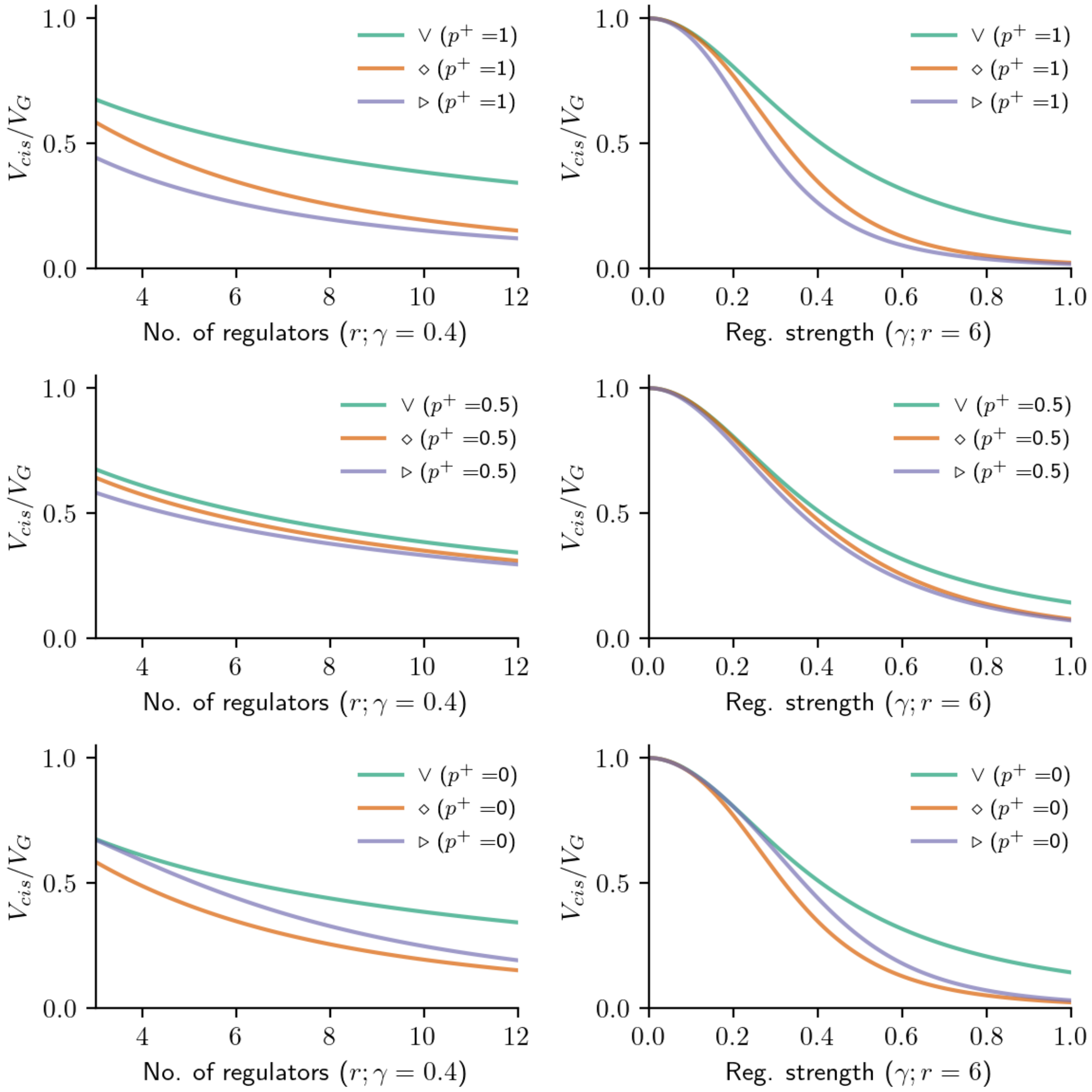

Figure S3: Effect of motifs with different fractions of activators, related to **Fig. 2**. Representative effects of the number of regulators (r; left panels) and the strength of regulation (γ; right panels) on the distribution of *cis*-acting variance for the three motifs in **Fig. 2**. The underlying mathematical equations for vee, triangle, and diamond motifs are as in the main text and corresponding figure — here, the expressions are also stratified by representative fractions of activators p^+ (the panels in the top row are exactly as in **Fig. 2**).

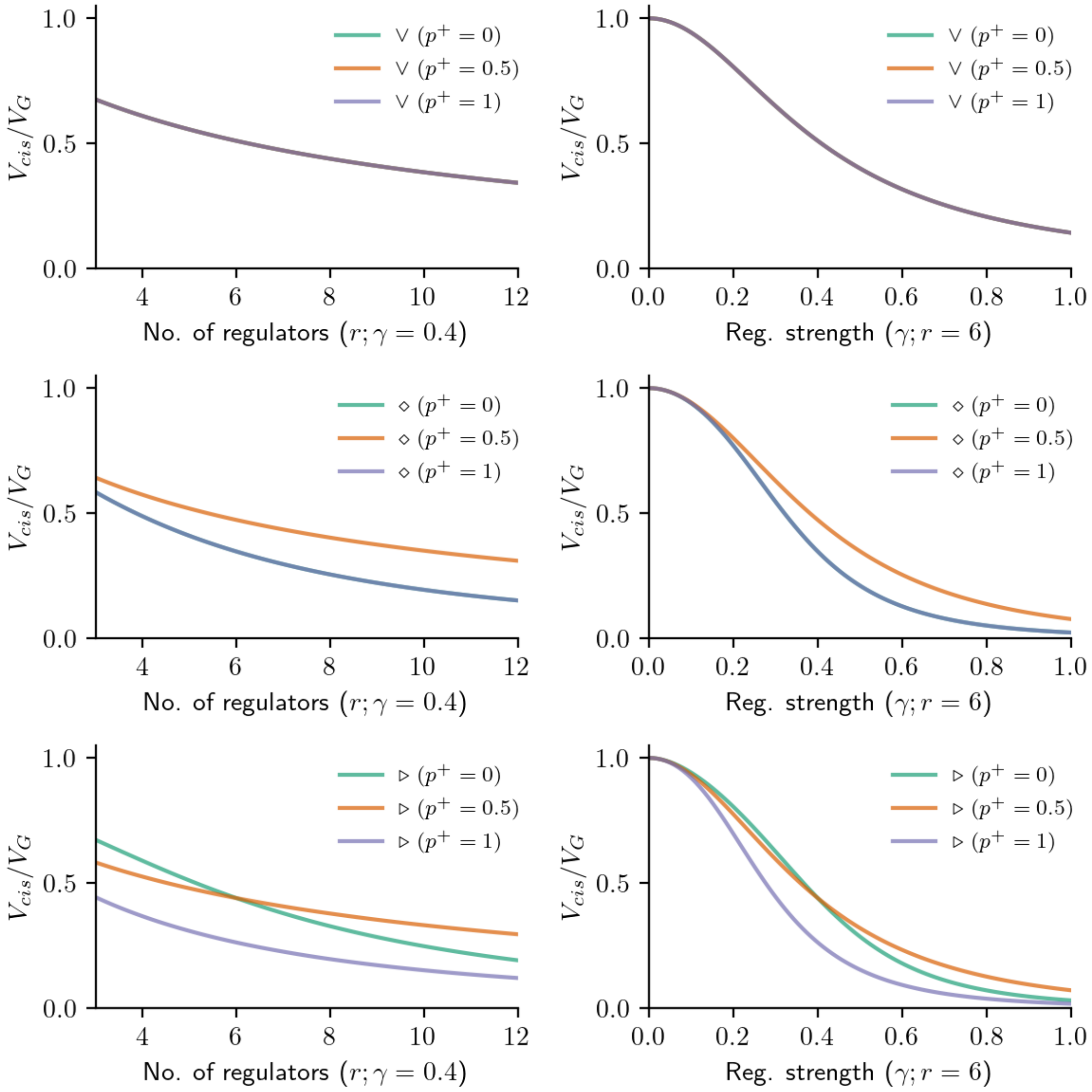

Figure S4: Effect of regulatory sign on different motifs, related to **Fig. 2**. Representative effects of the number of regulators (r; left panels) and the strength of regulation (γ; right panels) on the distribution of *cis*-acting variance for the three motifs in **Fig. 2**. The lines in this plot are the same as in **Fig. S3**, but subpanels here correspond to the separate motifs (vee, diamond, and triangle, in each row) rather than distinct values for the fraction of activators (p^+).

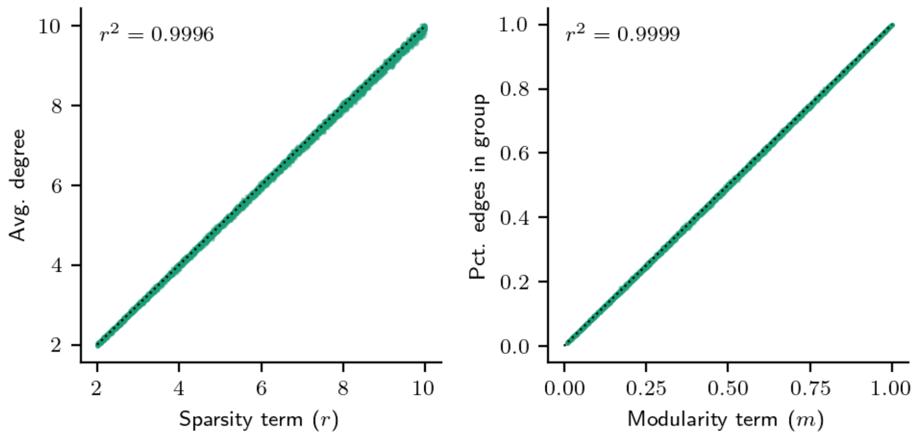

Figure S5: Parameters of the planted partition model (PPM) control key graph properties, related to **Fig. 3**. Values for the sparsity term (r; left panel) and modularity term (m; right panel) in the $10{,}000$ synthetic GRNs generated using the planted partition model (see **Methods**). The sparsity term r is extremely correlated with the average degree in the resulting GRN, and the modularity term m is extremely correlated with the resulting fraction of edges in the GRN that are drawn between genes in the same group (rather than genes in separate groups).

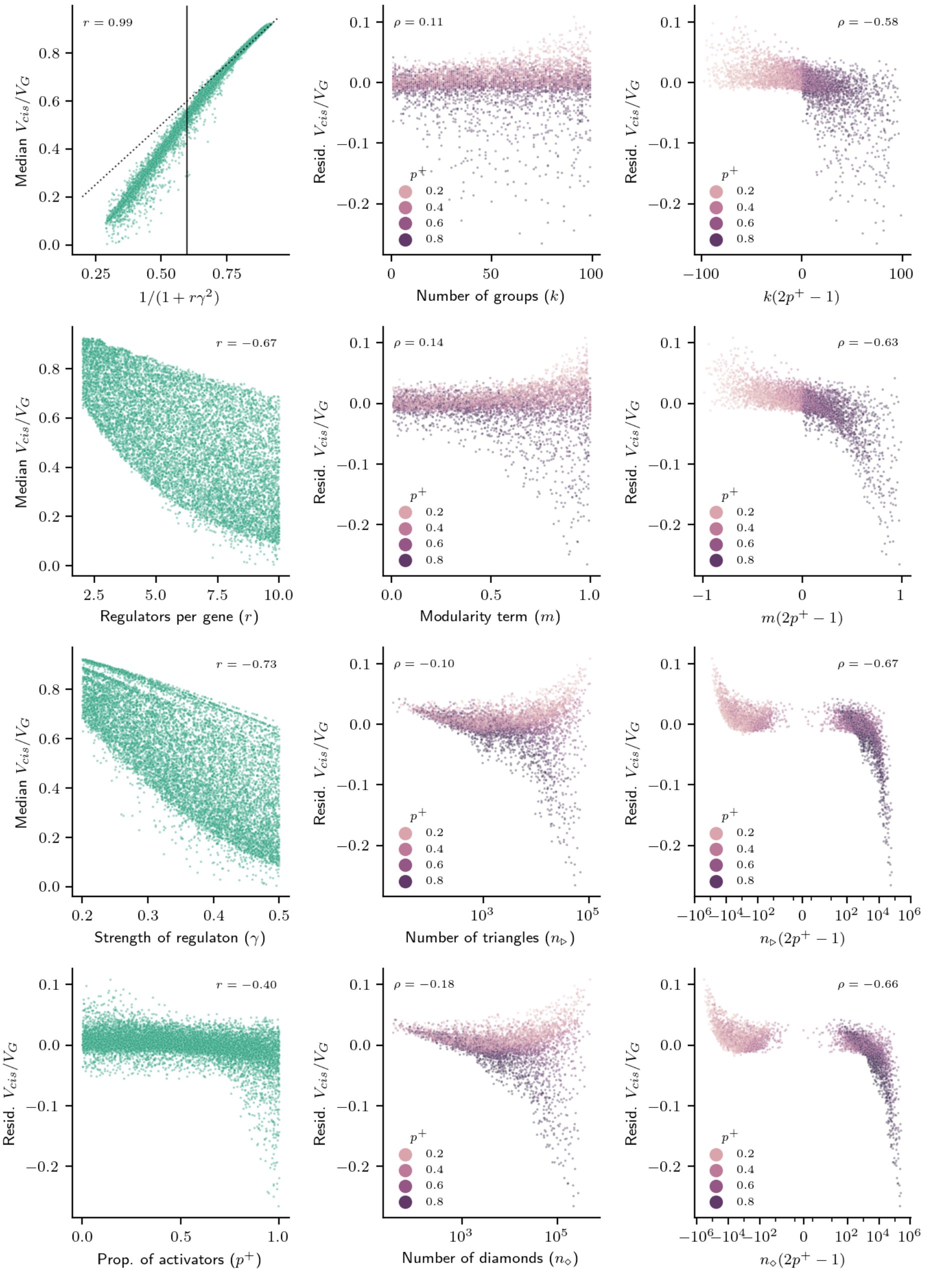

Figure S6: Parameters of the planted partition model (PPM) affect the distribution of heritability, related to **Fig. 3**. Relationships between properties of GRNs produced using the PPM and summaries of the median fraction of *cis*-acting expression variance in the corresponding $10{,}000$ GRNs. Subpanels are annotated by the Pearson (r) or Spearman (ρ) correlation between the quantities shown on the axes — median $V_{\text{cis}}/V_{\text{G}}$ denotes the untransformed value, while "Resid. $V_{\text{cis}}/V_{\text{G}}$" denotes the residual having regressed out direct effects as $1/(1+r\gamma^2)$. Networks in the middle and right columns are shown stratified by the fraction of activators p^+ and are subsetted to GRNs with lower expected contributions from direct effects $(1/(1+r\gamma^2) < 0.6)$, denoted by a solid vertical line in the top left panel (the dashed line is $y = x$); these are also the networks shown in **Fig. 3**.

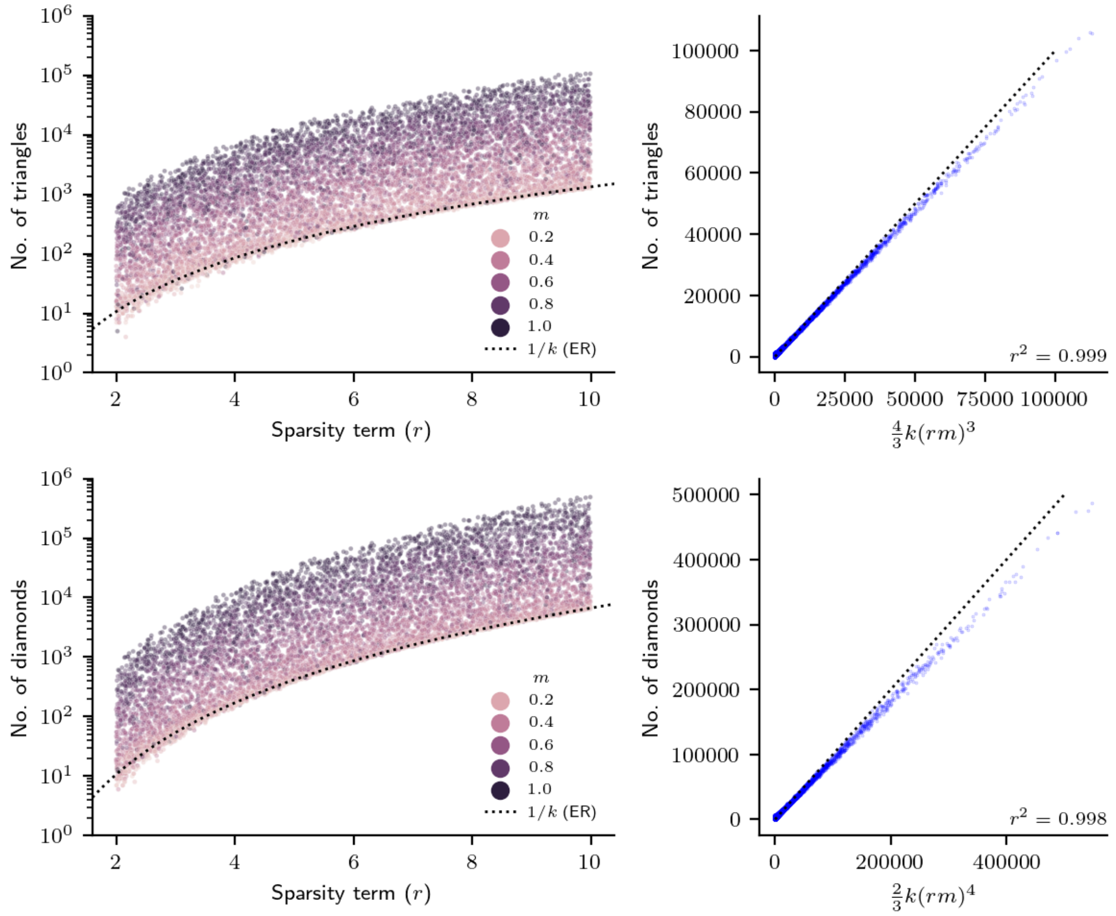

Figure S7: Parameters of the planted partition model (PPM) affect the number of motifs in the network, related to **Fig. 3**. Relationships between the sparsity (r) and modularity (m) parameters of the PPM and the resulting number of triangle and diamond motifs (defined as diagrammed in **Fig. 2**) in $10{,}000$ PPM GRNs. In both plots, each point is a network — the left panels show the interaction between r and m (note that $m = 1/k$ is the minimum value simulated in the study, and corresponds to the binomial graph); the right panels show the relationship between motif counts and the leading term of a mathematical approximation to the expected number of motifs.

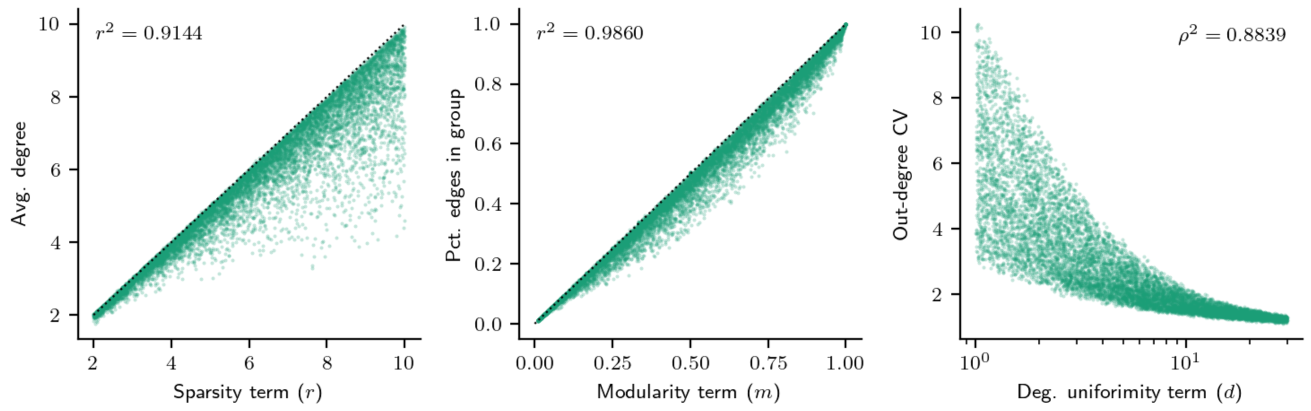

Figure S8: Parameters of the modular scale-free graph model control key graph properties, related to **Fig. 4**. Values for the sparsity term (r; left panel), modularity term (m; center panel), and degree uniformity term (d; right panel) in the $10{,}000$ synthetic GRNs generated using the modular scale-free graph generating algorithm (see **Methods**). The sparsity term r is strongly correlated with the average degree in the GRN. The modularity term m is strongly correlated with the fraction of edges in the GRN that are drawn between genes in the same group (rather than genes in separate groups). The degree uniformity term d is strongly correlated with the coefficient of variation (CV) of the out-degree distribution — here, CV is the standard deviation of the degree distribution over its mean, and a larger CV corresponds to a more dispersed distribution.

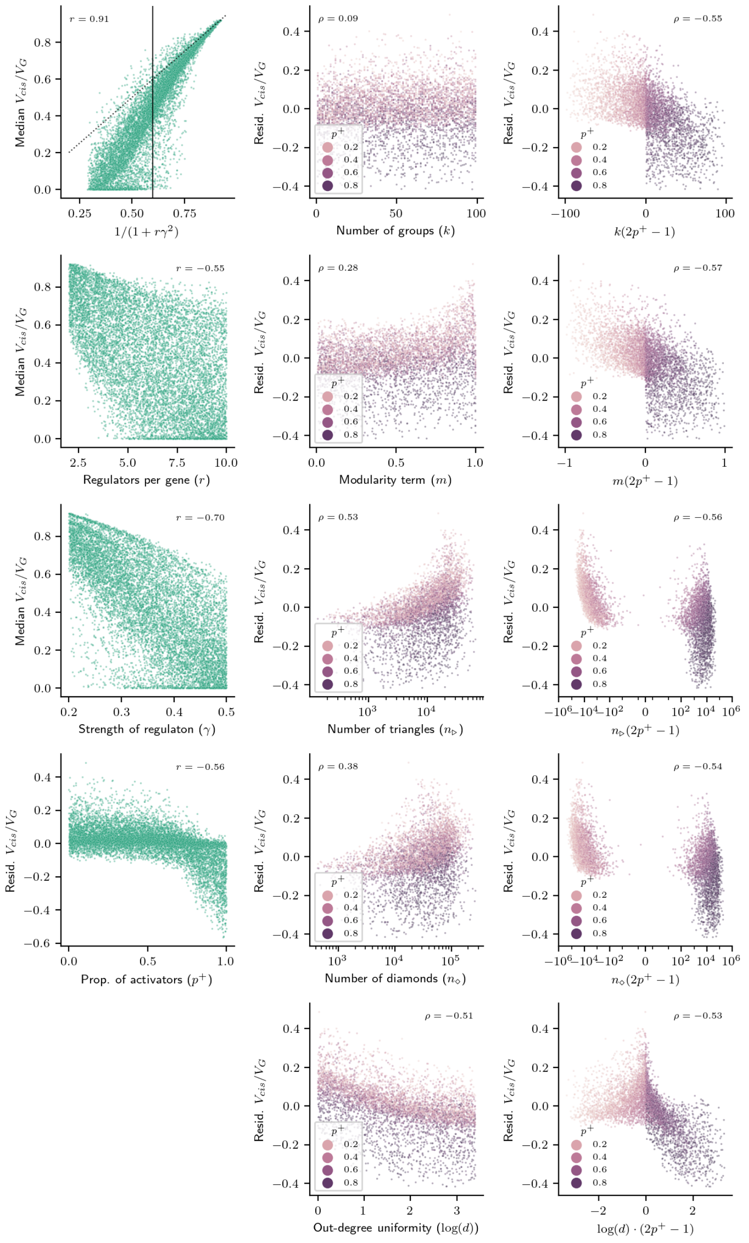

Figure S9: Parameters of the modular scale-free graph model affect the distribution of heritability, related to **Fig. 4**. Relationships between properties of GRNs produced using the modular scale-free graph model and summaries of the median fraction of *cis*-acting expression variance in the corresponding $10{,}000$ GRNs. Subpanels are annotated by the Pearson (r) or Spearman (ρ) correlation between the quantities shown on the axes — median $V_{\text{cis}}/V_{\text{G}}$ denotes the untransformed value, while "Resid. $V_{\text{cis}}/V_{\text{G}}$" denotes the residual variance after regressing out direct effects as $1/(1 + r\gamma^2)$. Networks in the middle and right columns are shown stratified by the fraction of activators p^+ and are subset to GRNs with lower expected contributions from direct effects ($1/(1 + r\gamma^2) < 0.6$, denoted by a solid vertical line in the top left panel (the dashed line is $y = x$); these are also the networks shown in **Fig. 4**.

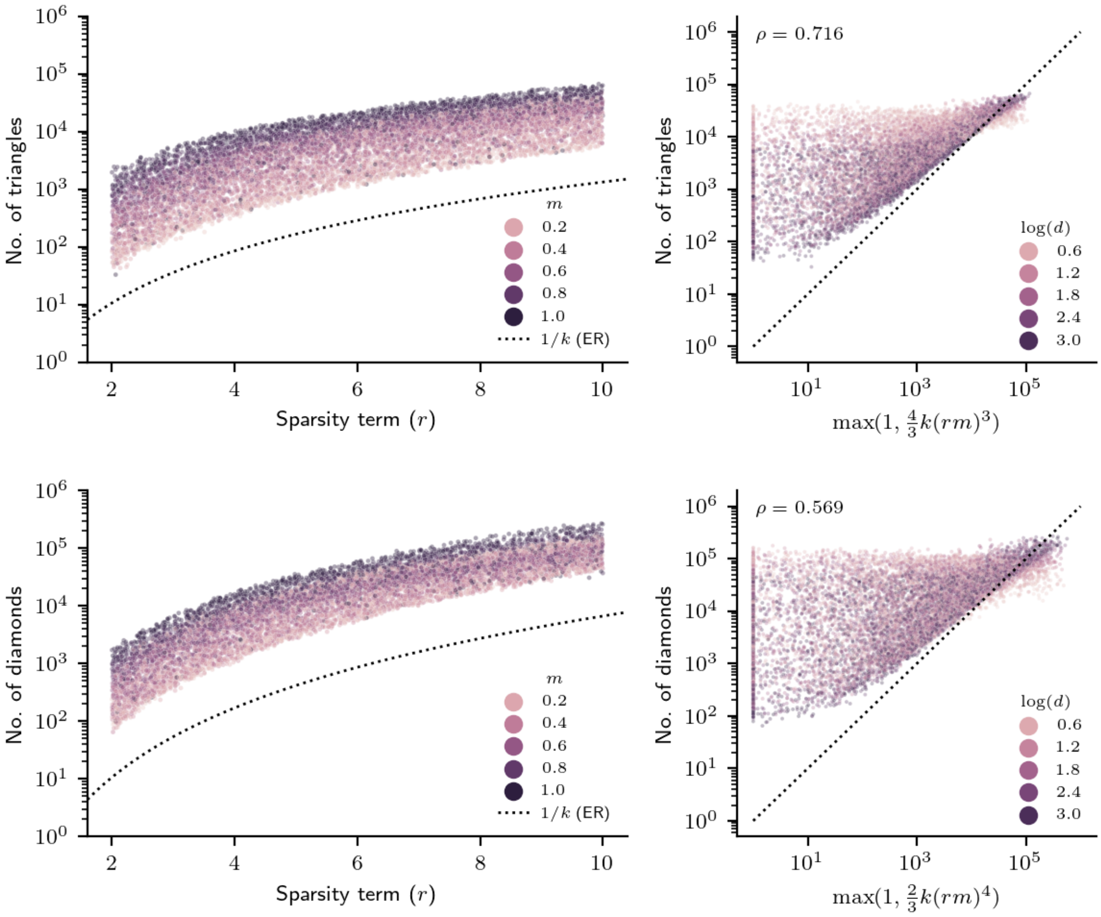

Figure S10: Parameters of the modular scale-free graph model affect the number of motifs in the network, related to **Fig. 4**. Relationships between the sparsity (r) and modularity (m) parameters of the modular scale-free graph model and the resulting number of triangle and diamond motifs (defined as diagrammed in **Fig. 2**) in $10{,}000$ GRNs. In both plots, each point is a network — the left panels show the interaction between r and m (note that $m = 1/k$ is the minimum value simulated in the study, and corresponds to the value which yields the binomial graph in the PPM); the right panels show the relationship between motif counts and the leading term of a mathematical approximation to the expected number of motifs to the PPM, and that smaller values of d (which introduce hubs into the graph) drive deviations from this expected value.

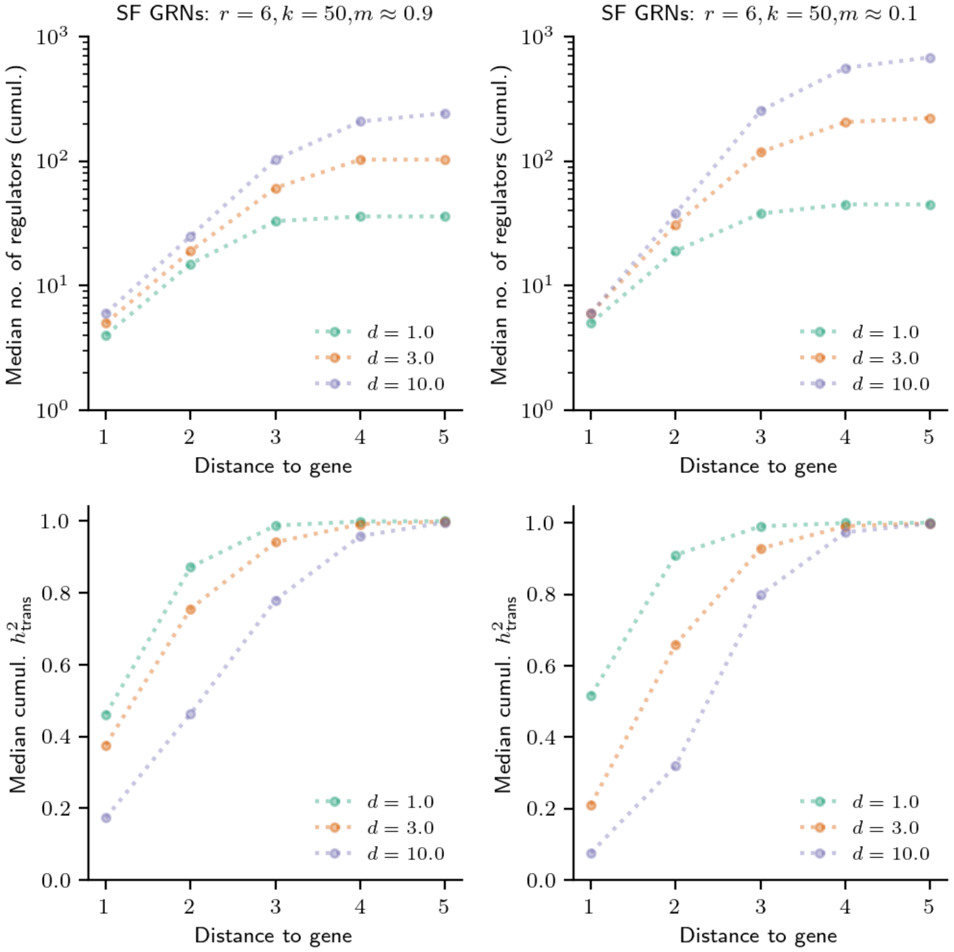

Figure S11: Degree uniformity alters the distribution of path lengths in the network, related to **Fig. 4**. Median number of regulators (top panels) and cumulative *trans*-acting expression variance (bottom panels) in six example modular scale-free GRNs, generated with identical terms for sparsity ($r = 6$), number of groups ($k = 50$), regulatory strength ($\gamma = 0.5$), and composition ($p^+ = 0.7$), but varying modularity ($m \approx 0.9$ or $m \approx 0.1$) or degree dispersion ($d = 1$, 3, or 10). Medians are over genes in the middle of the topological sorted order of the DAG (indexes 2000 to 3000). GRNs with regulatory hubs (smaller values of d) have fewer distant *trans*-regulators and *trans*-acting heritability is thus typically closer to a given gene in the network.

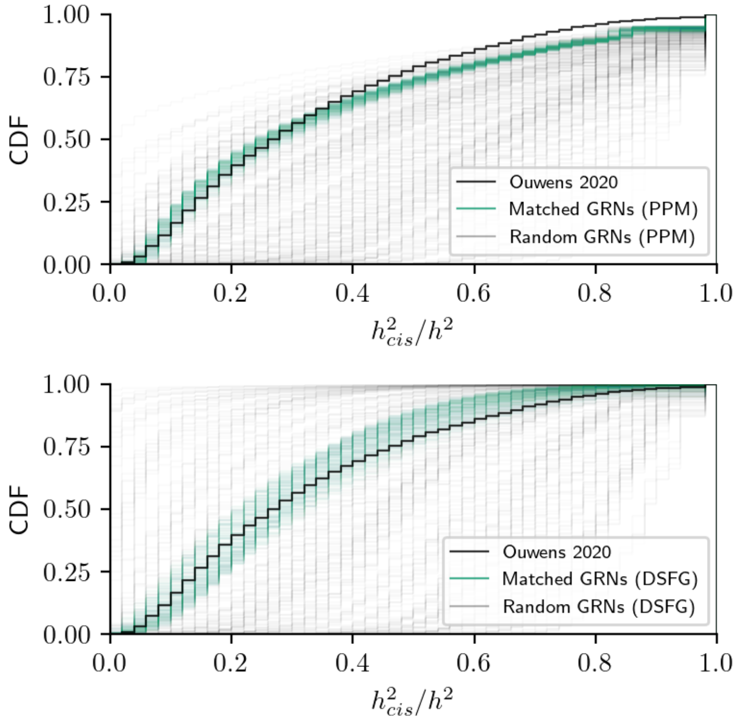

Figure S12: Distribution of heritability in synthetic GRNs and for whole-blood gene expression, related to **Fig. 5**. Distribution of the fraction of *cis*-acting heritability (h^2_{cis}/h^2) from real data (Ouwens *et. al.* 2020)[1] and from synthetic GRNs generated using the planted partition model (PPM; top panel) or the directed scale-free graph generating algorithm (SF; bottom panel). In both plots, teal lines are the 250 GRNs closest to the distribution from data (lowest K-S test statistics; see **Methods**), and grey lines are a random sample of 250 GRNs from the other $9{,}750$ simulated GRNs.

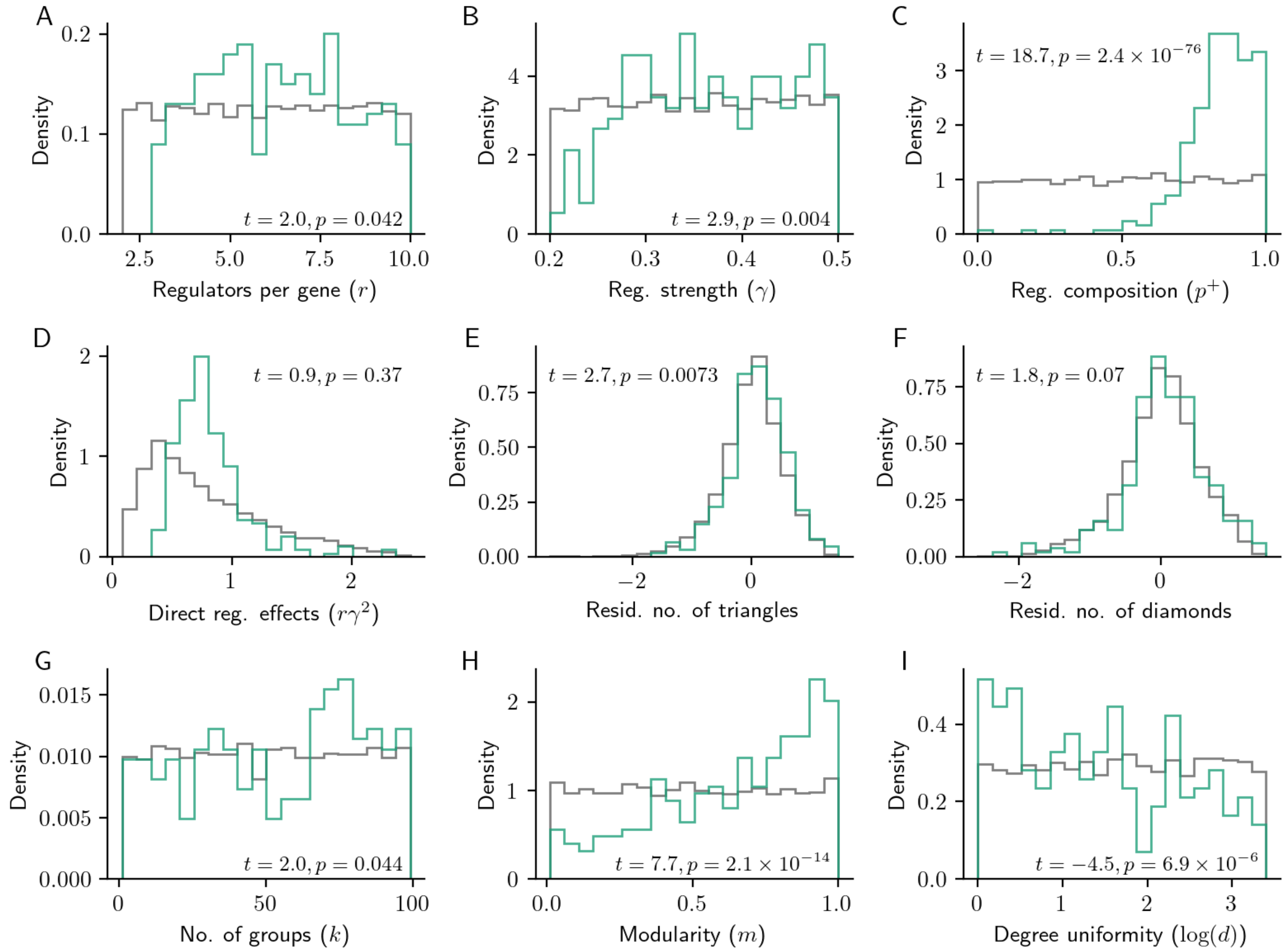

Figure S13: Properties of synthetic GRNs that best resemble real data, related to **Fig. 5**. Additional properties of the 250 GRNs that were best matched to the observed distribution of *cis*-acting heritability fractions (shown in teal, as in **Fig. 5**). Each panel is annotated with results from a two-sample *t*-test for a difference in mean with the remaining $9{,}750$ GRNs (shown in grey).

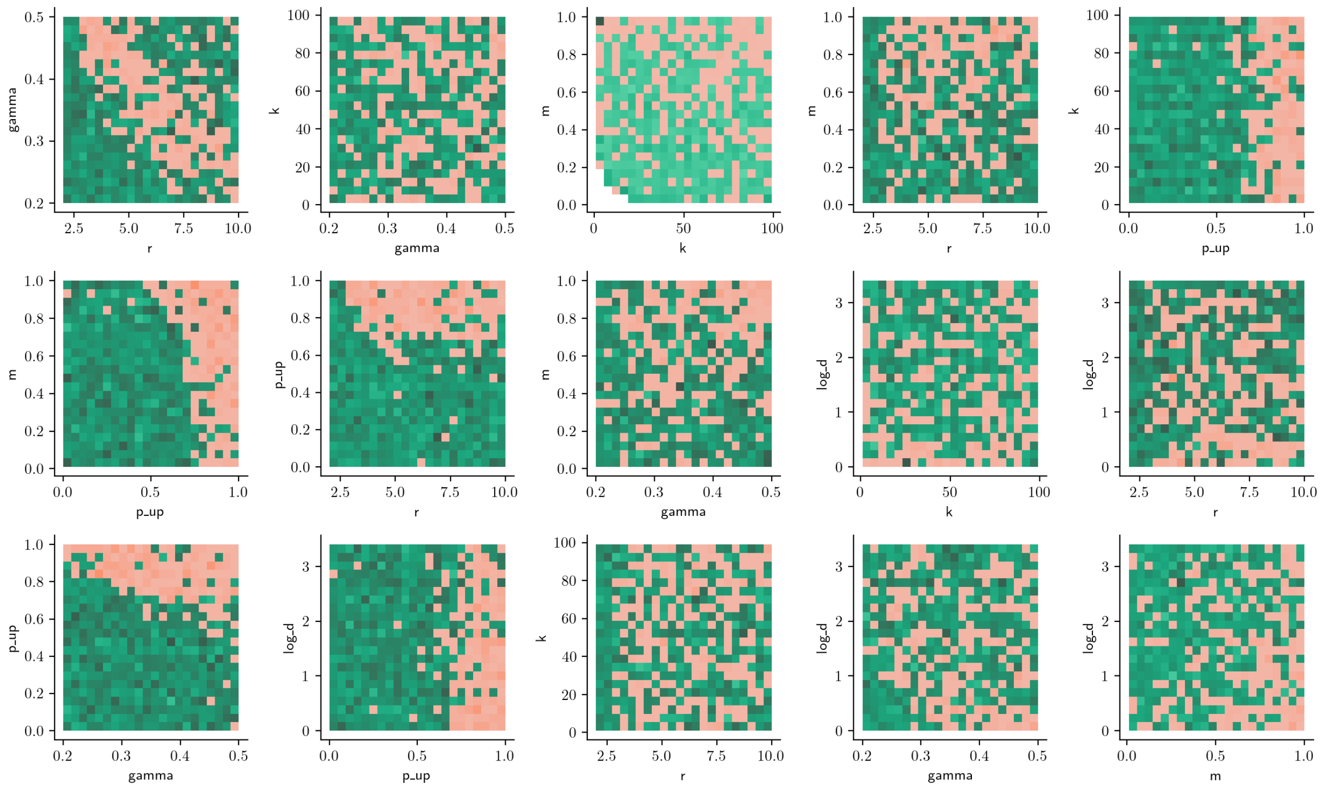

Figure S14: Interactions between properties of synthetic GRNs that best resemble real expression heritability data, related to **Fig. 5**. Pairwise interactions between properties of the 250 GRNs that were best matched to the observed distribution of *cis*-acting heritability fractions. In each subpanel, red denotes an enrichment of matched GRNs in and green denotes depletion.

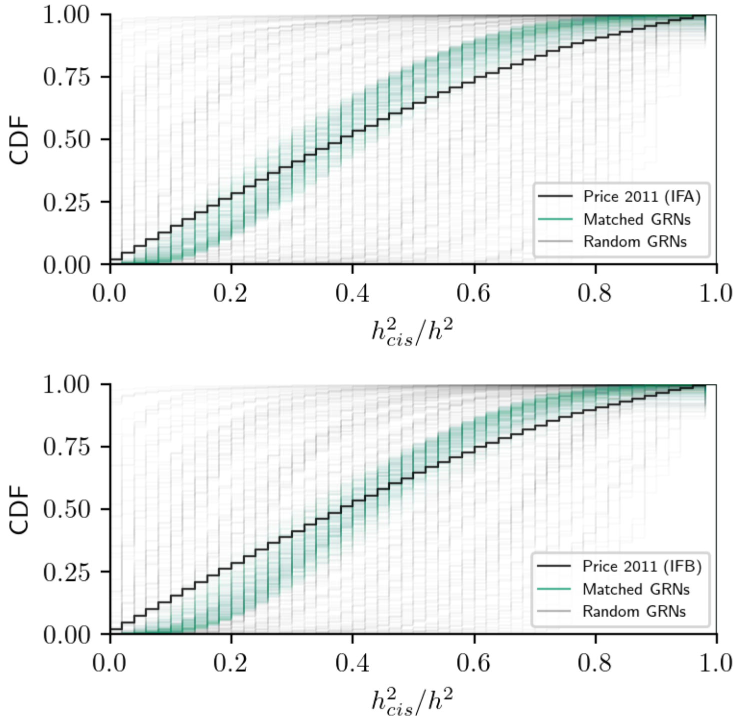

Figure S15: Distribution of heritability in synthetic GRNs and for whole-blood and adipose tissue gene expression, related to **Fig. 5**. Distribution of the fraction of *cis*-acting heritability (h^2_{cis}/h^2) from real data (Price *et. al.* 2011[2]; top panel [IFA] is adipose tissue, and bottom panel [IFB] is whole blood) and from synthetic GRNs generated using the directed scale-free graph generating algorithm. In both plots, teal lines are the 250 GRNs closest to the distribution from data (lowest K-S test statistics; see **Methods**), and grey lines are a random sample of 250 GRNs from the other $9{,}750$ simulated GRNs.

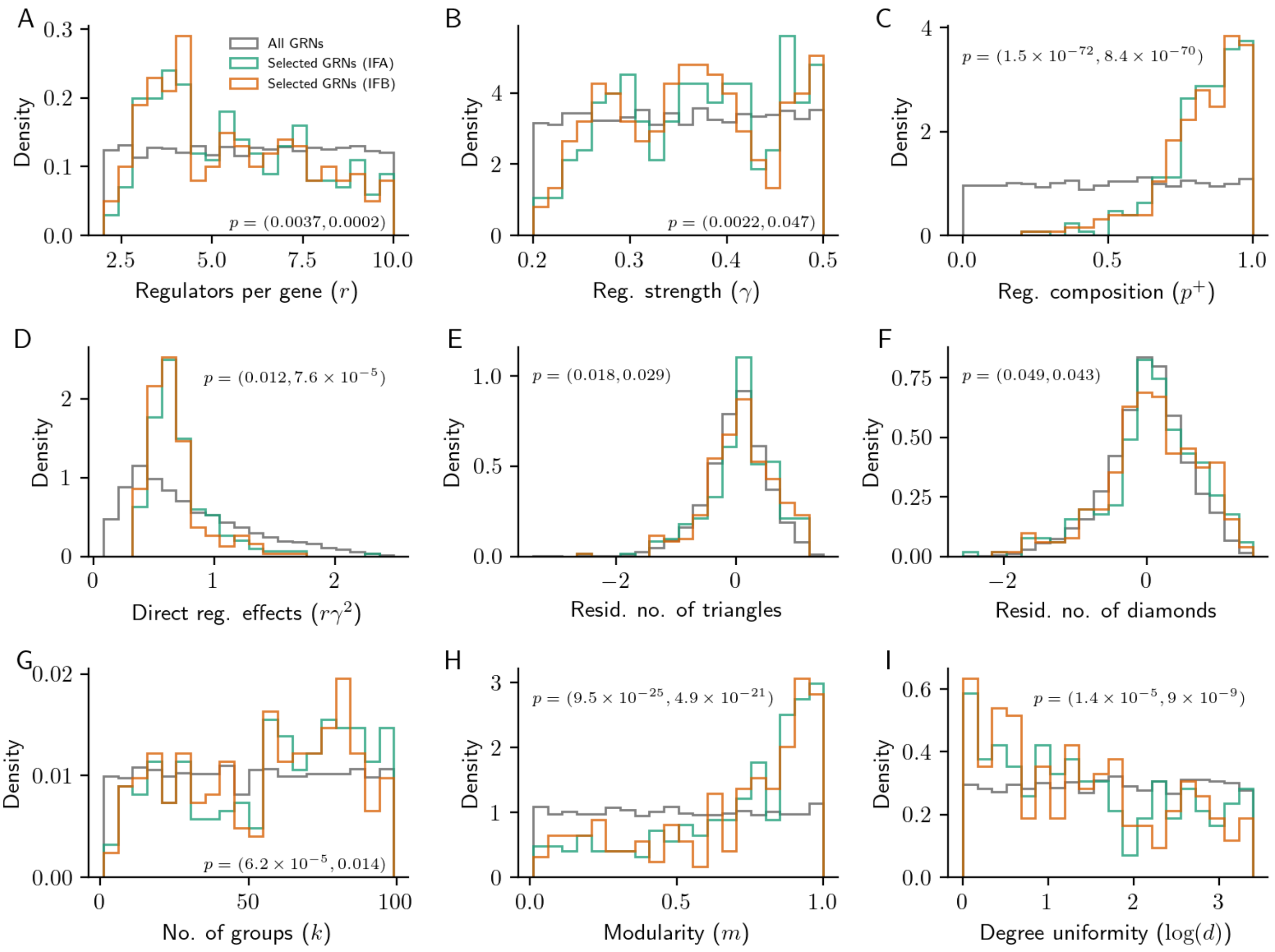

Figure S16: Properties of synthetic GRNs that best resemble replication data, related to **Fig. 5**. Additional properties of the 250 GRNs that were best matched to the observed distribution of *cis*-acting heritability fractions from the replication data (Price *et. al.* 2011[2]), as in **Fig. 5**. GRNs matched to results from adipose (IFA) are shown in teal, GRNs matched to results from whole blood are shown in orange, and unmatched GRNs are shown in grey. Each panel is annotated with results from a two-sample *t*-test for a difference in mean with the remaining $9{,}750$ GRNs (shown in grey).

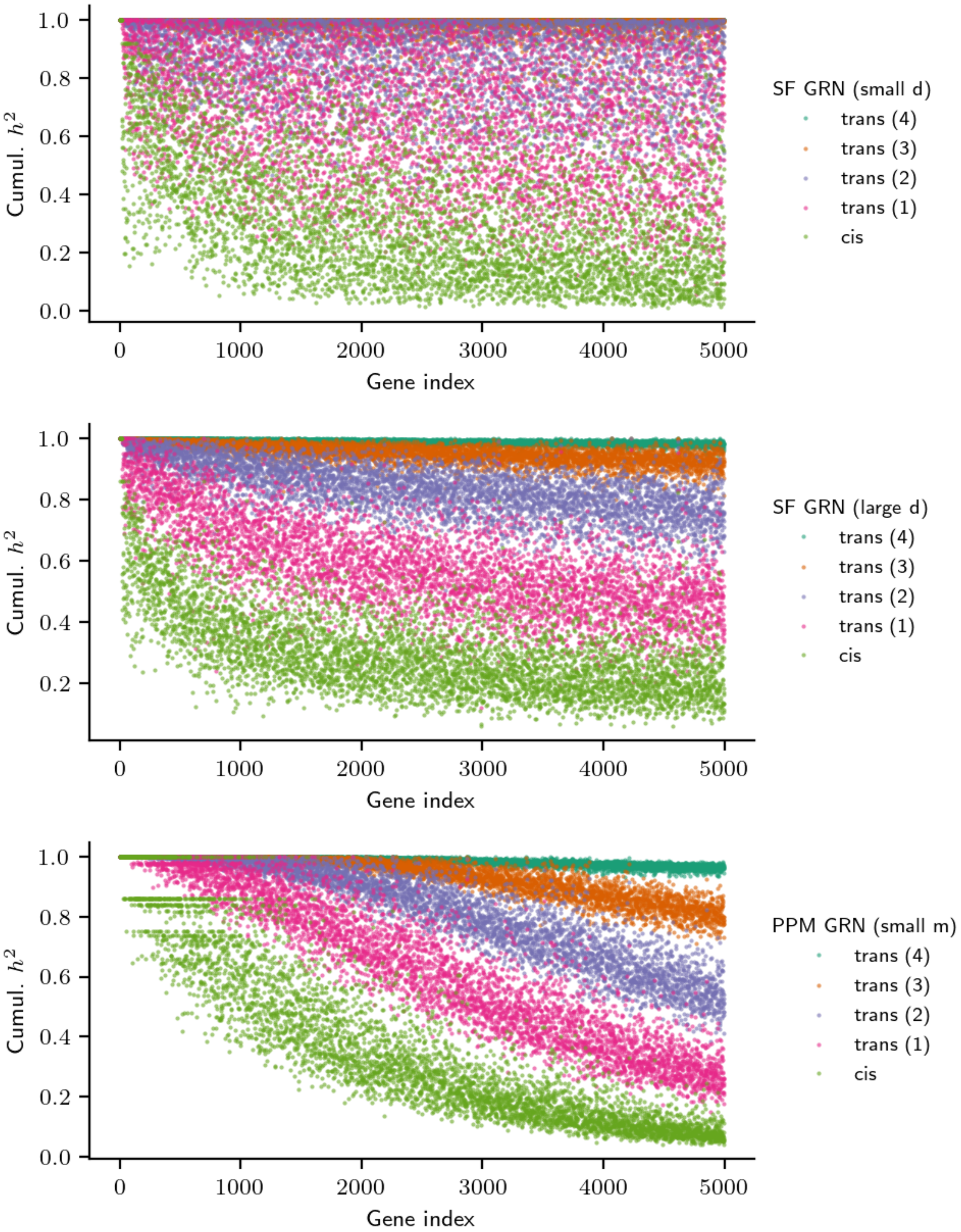

Figure S17: Gene-level heritability distribution over network distances in three example GRNs, related to **Fig. 6**. Cumulative heritability in *cis* and in *trans*, stratified by distance, for all genes in the three example networks from **Fig. 6**.

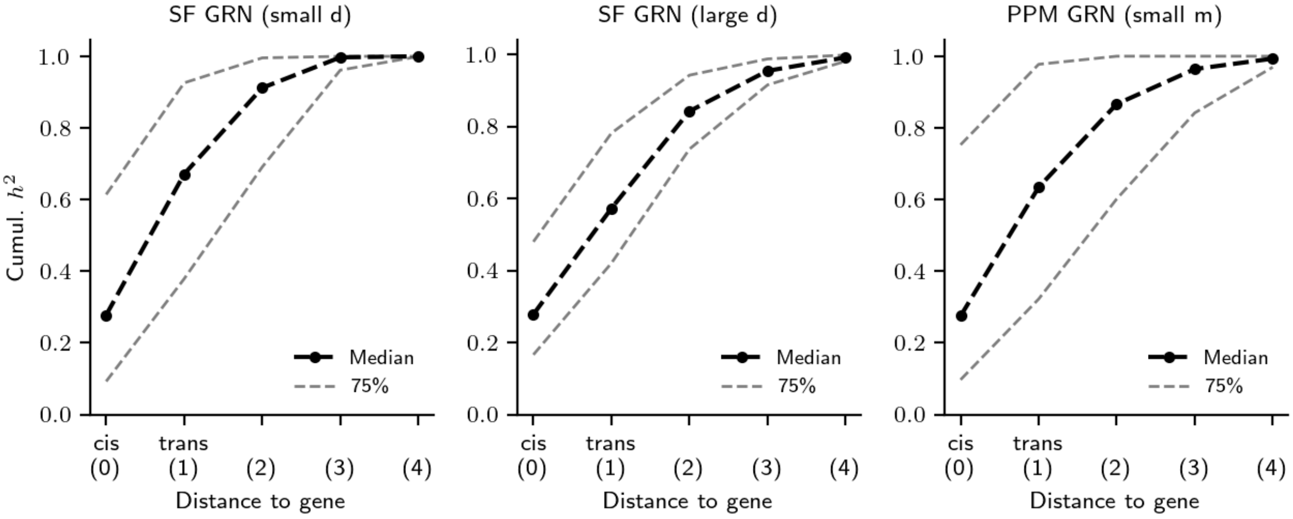

Figure S18: Cumulative heritability distribution over network distances in three example GRNs, related to **Fig. 6**. Cumulative heritability in *cis* and in *trans*, stratified by distance, for the median gene (or quartiles) in the three example networks from **Fig. 6B** — note that the medians and quantiles are computed separately for each tick in the x-axis (i.e., the gene with the median *cis*-acting heritability fraction may not be the gene with the median one-hop *trans*-acting heritability fraction).

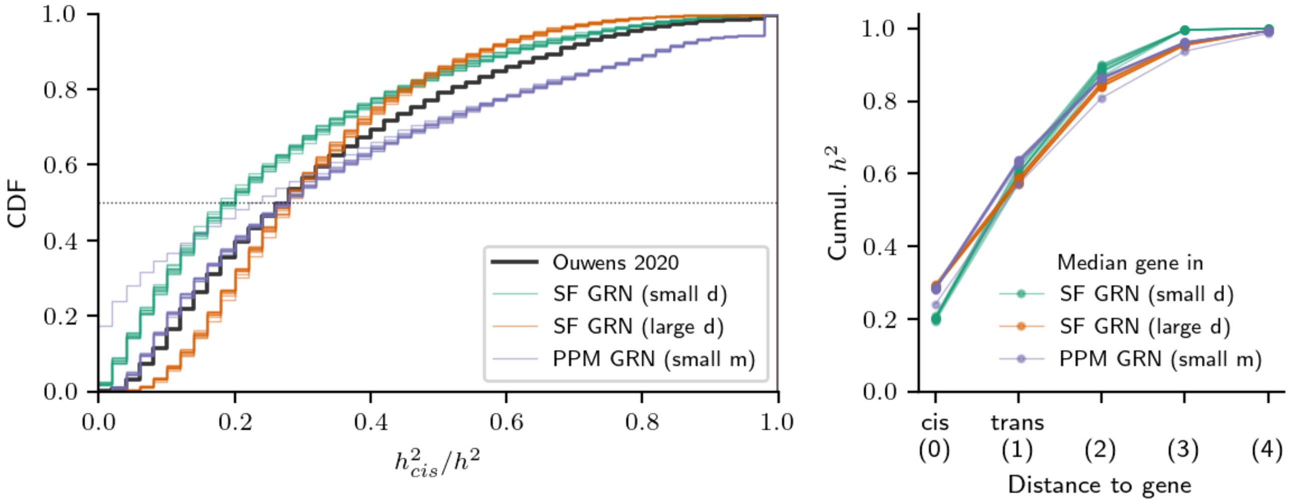

Figure S19: Median *cis*- and cumulative heritability over distances in several example GRNs, related to **Fig. 6**. Replication of results in **Fig. 6A-B**. Thirty GRNs in this figure have the same edges as the three example GRNs in **Fig. 6**, but have resampled parameters of the gene expression model, taking $|\gamma|_i \sim_{iid} \mathcal{N}(\gamma, \gamma/5)$ for each gene i (10 resampled GRNs for each in the main Figure). **(A)** Cumulative distribution of the fraction of *cis*-acting expression variance in each resampled GRN, plotted against the distribution from real data (Ouwens et. al., 2020)[1] **(B)** Median cumulative heritability as a function of network distance in each resampled GRN. Median is over genes in the network; cis-effects are at distance 0, trans-effects from direct regulators are at distance 1, etc.

References

1. Ouwens, K.G., Jansen, R., Nivard, M.G., van Dongen, J., Frieser, M.J., Hottenga, J.J., Arindrarto, W., Claringbould, A., van Iterson, M., Mei, H., Franke, L., Heijmans, B.T., A. C. 't Hoen, P., van Meurs, J., Brooks, A.I., Penninx, B.W.J.H., and Boomsma, D.I. (2020). A characterization of cis- and trans-heritability of RNA-Seq-based gene expression. Eur J Hum Genet *28*, 253–263. URL: https://www.nature.com/articles/s41431-019-0511-5. doi: 10.1038/s41431-019-0511-5. Publisher: Nature Publishing Group.

2. Price, A.L., Helgason, A., Thorleifsson, G., McCarroll, S.A., Kong, A., and Stefansson, K. (2011). Single-Tissue and Cross-Tissue Heritability of Gene Expression Via Identity-by-Descent in Related or Unrelated Individuals. PLOS Genetics *7*, e1001317. URL: https://journals.plos.org/plosgenetics/article?id=10.1371/journal.pgen.1001317. doi: 10.1371/journal.pgen.1001317. Publisher: Public Library of Science.
