# Supplementary material for: Regulatory network topology and the genetic architecture of gene expression
Source: Cell Genom. 2026 Apr 22;6(6):101219. doi: 10.1016/j.xgen.2026.101219 (PMC13261694; doi:10.1016/j.xgen.2026.101219)
Supplement: Document S2. Article plus supplemental information [file mmc2.pdf]

Cell Genomics

Article

Regulatory network topology and the genetic architecture of gene expression

Graphical abstract

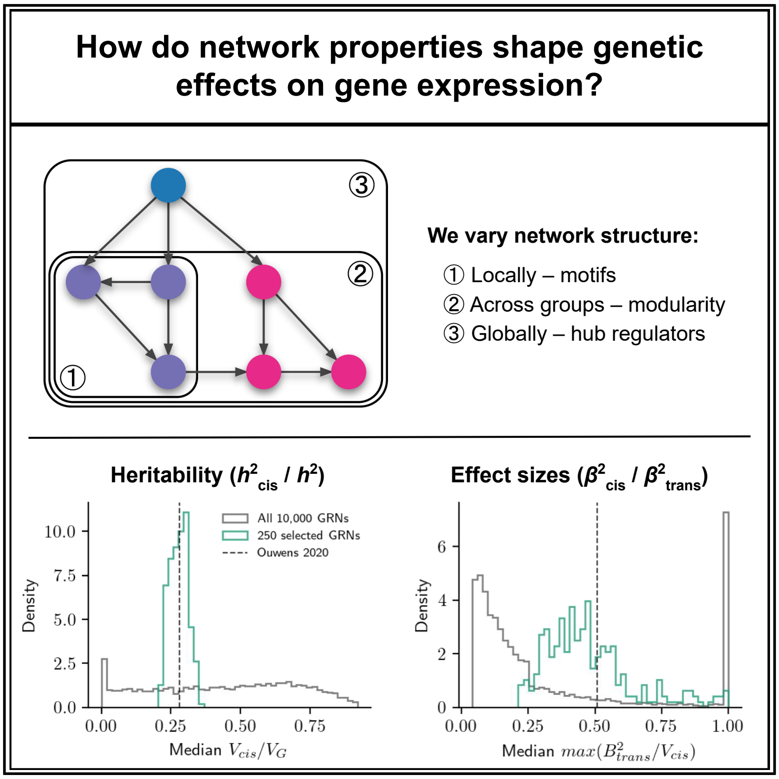

Authors

Matthew Aguirre, Jeffrey P. Spence, Guy Sella, Jonathan K. Pritchard

Correspondence

aguirm18@gene.com (M.A.),
jeff.spence@ucsf.edu (J.P.S.),
gs2747@columbia.edu (G.S.),
pritch@stanford.edu (J.K.P.)

In brief

Aguirre et al. describe a model of gene regulatory networks and show how their properties shape the distribution of genetic effects on gene expression. Their results imply constraints on real network structures and suggest that the architecture of gene expression is less polygenic but more pleiotropic than previously anticipated.

Highlights

- Model key properties of regulatory networks and gene expression data
- Sparsity, modularity, and hub regulators shape the distribution of *trans*-eQTLs
- All of these properties are needed to match real eQTL data
- Gene expression is less polygenic but more pleiotropic than previously anticipated

Aguirre et al., 2026, Cell Genomics *6*, 101219
June 10, 2026 © 2026 The Author(s). Published by Elsevier Inc.
https://doi.org/10.1016/j.xgen.2026.101219

CellPress

Cell Genomics

CellPress OPEN ACCESS

Article

Regulatory network topology and the genetic architecture of gene expression

Matthew Aguirre,[1,8,9,*] Jeffrey P. Spence,[2,3,4,*] Guy Sella,[5,6,*] and Jonathan K. Pritchard[2,7,*]
[1]Department of Biomedical Data Science, Stanford University, Stanford, CA, USA
[2]Department of Genetics, Stanford University, Stanford, CA, USA
[3]Institute for Human Genetics, University of California, San Francisco, San Francisco, CA, USA
[4]Department of Epidemiology & Biostatistics, University of California, San Francisco, San Francisco, CA, USA
[5]Department of Biological Sciences, Columbia University, New York, NY, USA
[6]Program for Mathematical Genomics, Columbia University, New York, NY, USA
[7]Department of Genetics, Stanford University, Stanford, CA, USA
[8]Present address: Genentech, Inc., South San Francisco, CA 94080, USA
[9]Lead contact
*Correspondence: aguirm18@gene.com (M.A.), jeff.spence@ucsf.edu (J.P.S.), gs2747@columbia.edu (G.S.), pritch@stanford.edu (J.K.P.)
https://doi.org/10.1016/j.xgen.2026.101219

SUMMARY

Most genetic variance in gene expression is due to *trans*-acting expression quantitative trait loci (eQTLs) spread across the genome. However, these loci are generally hard to map due to limited discovery power. Here, we simulate how local properties of expression regulation and global properties of regulatory networks alter the genome-wide proportions of *cis*- and *trans*-heritability. We find that network motifs and modular groups can reduce or enhance the effects of *trans*-eQTLs and that hub regulators shorten paths across the network and act as key sources of *trans*-acting variance. Critically, networks with all these features best recapitulate the observed distribution of *cis*- and *trans*-heritability. Taken together, our results suggest that the genome-wide genetic architecture of gene expression involves fewer regulators for each gene but implicates the same regulators more often across genes (i.e., is less polygenic and more pleiotropic) than previously anticipated.

INTRODUCTION

Gene expression is widely thought to be an important mediator of the effects of non-coding genetic variation, which comprises the majority of hits from genome-wide association studies (GWASs).[1–3] However, one major surprise from human genetic studies of gene expression has been that expression quantitative trait loci (eQTLs) close to a given gene tend to explain a minority of its genetic variance. This fraction of *cis*-acting heritability, h^2_{cis}, is estimated to have a genome-wide median of around 20% in studies of bulk tissue.[4–7] Similarly, *cis*-eQTLs explain a modest fraction of the heritability of many complex traits and diseases and exhibit systematic differences with leading hits from GWASs.[8,9]

Still, *cis*-eQTLs have offered mechanistic insight into GWAS signals through approaches including statistical colocalization and transcriptome-wide association studies,[10–13] even as it is debated whether most trait-relevant genetic variation can be discovered using assays of steady-state expression in bulk tissue.[14] But meanwhile, *trans*-eQTL discovery and analysis have been hampered by limited statistical power due to the higher multiple testing burden of genome-wide analysis and the likely smaller effect sizes of *trans*-eQTLs compared to *cis*-eQTLs. Even though *trans*-acting variants are thought to explain the majority of gene expression heritability, their number and distribution throughout the genome remain unclear.[6,15]

At the same time, efforts to further map the genetic architecture of gene expression have seen growth in the size and resolution of functional genomics studies,[3,16–20] with concurrent development of inference methods that scale to these data.[21–23] These advances have increasingly relied on models of gene regulatory networks (GRNs), as more data are being used to assay real GRNs in many cell types and contexts. Specifically, network properties such as modularity and regulatory hierarchy—the ideas that transcriptional master regulators control coherent gene sets and can direct cell-type differentiation, respectively—have been used to motivate considerations around cell-type composition effects in bulk and single-cell eQTL studies,[16,17,22] as well as aggregation tests for *trans*-eQTLs.[21,24] The relevance of these properties beyond gene expression has also been considered: in particular, genetic covariance induced by the structure of regulatory networks has been hypothesized to explain a substantial proportion of *trans*-acting heritability in complex traits.[5,6]

Here, we assess the implications of two simple and well-replicated observations about the distribution of expression heritability in bulk tissue—namely, that h^2_{cis} is low for the typical gene and that *cis*-eQTLs have categorically larger effects than *trans*-eQTLs. We use a linear causal model of gene expression to build intuition about key local and global properties of regulatory networks. Next, we analyze the effects of these properties, showing how they can buffer or enhance the effects of *trans*-acting

© 2026 The Author(s). Published by Elsevier Inc.
This is an open access article under the CC BY license (http://creativecommons.org/licenses/by/4.0/).

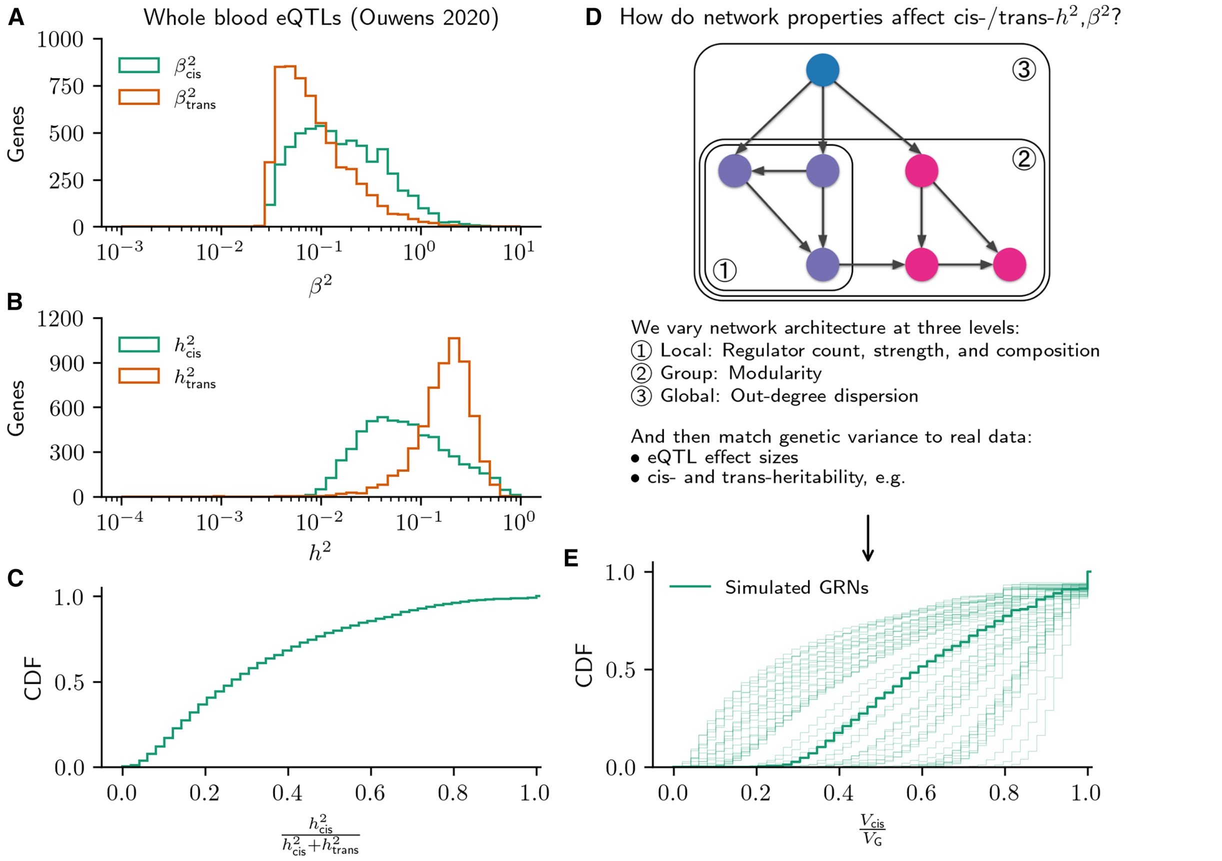

Figure 1. Overview of the study

(A and B) Lead eQTL effect sizes (A) and heritability (B) from *cis*- and *trans*-acting genetic effects for 5,902 genes from a recent twin study of whole blood gene expression.

(C) The cumulative distribution of *cis*-acting expression heritability from these same genes.

(D) Schematic of network properties and generating algorithm. In synthetic GRNs, nodes represent genes, and arrows represent regulatory interactions. Our GRN model has terms that relate to network architecture at (1) the local level, as shown by the purple nodes arranged in a triangle motif; (2) the group level, represented by the second module of pink nodes; and (3) the global level, as exemplified by the blue node (a hub regulator).

(E) Cumulative distribution of *cis*-acting expression heritability in 50 example synthetic GRNs. The overlay is the median of these distributions.

See also Figures S1 and S2.

variation. Finally, we show that the observed distribution of *cis*- and *trans*-heritability constrains the space of plausible regulatory network structures, with the most realistic GRNs having a sparse architecture with master regulators and modular groups. In these GRNs, the bulk of *trans*-acting expression variance can be found along short paths in the network and at key genes with many pleiotropic effects. These features may therefore be useful in further mapping real genetic effects on gene expression.

RESULTS

Genetic effects on gene expression

We motivate our work with data from a recent analysis of whole-blood gene expression from a twin study design to discover eQTLs and estimate heritability.[7] The data consist of eQTL summary statistics for 5,902 genes and heritability estimates for 11,409 protein-coding genes measured in 1,497 individuals (STAR Methods). Similar to other studies,[4,6,16] the typical lead *cis*-eQTL effect size is 0.14 (standard deviations of expression), which is an order of magnitude greater than the typical lead *trans*-eQTL effect size (0.07; both are medians over 5,902 genes with a *cis*-eQTL; Figure 1A). Meanwhile, the contributions to heritability are reversed, with *trans*-acting variation contributing the bulk of expression variance (Figure 1B). The typical fraction of *cis*-acting heritability, $h^2_{cis}/(h^2_{cis} + h^2_{trans})$, is 0.28 (median over 5,902 genes; Figure 1C).

In this study, we use a two-part model of how genetic variation affects gene expression through GRNs. The first component of the model is a graph-generating algorithm, which we use to change the architecture of the causal network (of *trans*-acting regulators). Here, network architecture includes regulatory properties, such as the number and strength of regulators, and organizational properties, such as group structure and hierarchy (Figure 1D; STAR Methods). The second component of the model is a linear structural equation model (SEM) of genetic effects on gene expression, which we use to measure the effects

CellPress
OPEN ACCESS

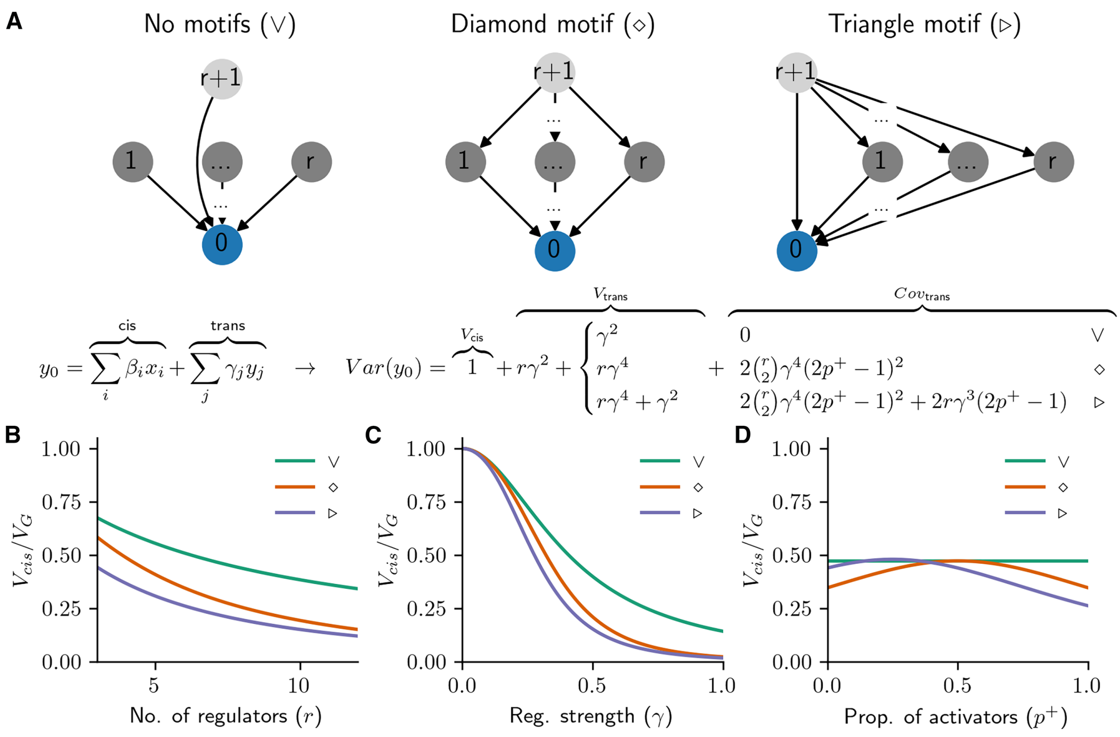

Figure 2. Local regulatory motifs

(A) Linear causal model of *cis*- and *trans*-eQTL effects on a focal gene (0), with three different ways to add a new regulator ($r + 1$): as a direct regulator (no/V-motif), as a peripheral master regulator (diamond motif), or as both (triangle motif). We assume that *cis*-eQTLs contribute unit variance to each gene shown, which results in the *cis*- and *trans*-acting contributions to genetic variance given below the motif diagrams.

(B–D) Plots of this expression for each of the three motifs as a function of the number of regulators per gene, r (B), the strength of regulation, γ (C), and the proportion of regulators that are activators, p^+ (D). Note that at the bottom of the plots, unless otherwise stated, $r = 6$, $\gamma = 0.4$, and $p^+ = 1$.

See also Figures S3 and S4.

of network properties on the distribution of expression variance throughout the network. Specifically, we consider the size of typical lead *trans*-eQTL effects relative to lead *cis*-eQTL effects, $\beta^2_{trans}/\beta^2_{cis}$, and the typical fraction of heritability due to *cis*-eQTLs, h^2_{cis}/h^2 (Figure 1E).

Local regulatory architecture

Following previous work in GRN inference[25,26] and quantitative genetics,[6,27] we use a linear model of gene expression regulation under the assumption that genetic effects on gene expression are generally small. This model is a SEM in which the expression, y, of a focal gene is determined by the effects of q *cis*-eQTLs (each with effect β_i from genotype x_i) and r regulators (each with effect γ_j):

$$y = \underbrace{\sum_{i=1}^{q} x_i\beta_i}_{\text{cis}} + \underbrace{\sum_{j=1}^{r} y_j\gamma_j}_{\text{trans}} + s; \underbrace{s \sim f\left(0, \sigma^2\right)}_{\text{noise}}. \quad \text{(Equation 1)}$$

A diagram of the biological interpretation of this model is shown in Figure S1. Here, for simplicity, we assume that all regulators have the same strength, γ, but that their effects can differ in sign: in particular, we assume gene j will act consistently as an activator for all of its targets with probability p^+ and is otherwise a repressor. That is, with probability p^+, the effect of a gene on the expression of all of its targets is $+\gamma$, and with probability $(1 - p^+)$, the effect is $-\gamma$. We further assume that *cis*-eQTLs are independent and collectively contribute unit variance to each gene. The variance of the expression of the focal gene across individuals is then

$$Var(y) = \underbrace{1}_{\text{cis}} + \underbrace{r\gamma^2 + 2\gamma^2 \sum_{j=1}^{r}\sum_{j'=1}^{j-1} \text{sign}\left(\gamma_j\gamma_{j'}\right)\cdot Cov\left(y_j, y_{j'}\right)}_{\text{trans}}. \quad \text{(Equation 2)}$$

We will first use this model to show how local aspects of regulatory architecture—namely, the number, strength, and sign of regulators—affect how heritability and genetic effects on the expression of a focal gene are distributed among its regulators. Later, we will consider properties of entire regulatory networks. A more detailed description of the expression model and its assumptions can be found in the STAR Methods.

To show how properties of local regulatory architecture influence expression variance, we consider three different roles for one specific regulator of a gene, which we suppose has $r + 1$ regulators. If all of the regulators of this gene are independent, as in Figure 2A (left), each regulator contributes *trans*-acting genetic variance with magnitude γ^2, lowering the *cis*-fraction of

heritability by the same amount (Figures 2B and 2C). This is true regardless of how many of the regulators act as activators or repressors (Figures 2D and S3).

If the regulator is instead a peripheral master regulator (i.e., a gene that regulates the direct regulators of the focal gene), it can contribute *trans*-acting genetic covariance in addition to its direct effects on the focal gene (Figure 2A, middle and right). The expected magnitude and sign of this covariance depend not only on the number of existing regulators, r, and their strength, γ, but also on the fraction of activators, p^+, and on whether the master regulator also directly regulates the focal gene (as in the "diamond" and "triangle" motifs; Figures 2B–2D; these are also called "bi-parallel" and "feedforward" motifs in the systems biology literature, respectively[28]). Further, these covariance terms are only nonzero in expectation over a random assignment of regulators as activators or repressors if these fractions are unequal ($p^+ \neq \frac{1}{2}$; Figure 2D; STAR Methods).

Because of the covariance introduced by master regulators, it is possible for an indirect regulator of a gene to contribute more to its *trans*-acting genetic variance than one of its direct regulators (Figures 2B, 2C, and S3). In particular, if p^+ is close to 1, motifs are more likely to be "coherent" (i.e., all paths from the master regulator to the target gene have the same sign[29]), and the expected *trans*-acting (co)variance is larger (Figure S4). Meanwhile, covariance introduced by "incoherent" motifs, where paths differ in sign,[29] is expected to be negative. Hence, there is an important difference in how genetic effects flow through triangle and diamond motifs under activating and repressing regulation (since the chance of a motif being coherent or incoherent is related to powers of $(2p^+ - 1)$; Figure 2D).

Modular network structure

Next, we consider the distribution of heritability and eQTL effects in the context of an entire GRN. Since we are interested in computing variances using a SEM, we simulate random directed acyclic graphs (DAGs) of causal regulatory relationships between genes. We do this using a standard random graph model, the planted partition model (PPM; STAR Methods).[30] Briefly, in the PPM, each of n nodes is assigned to one of k groups; edges exist between members of the same group with probability p or different groups with probability q. To produce DAGs with this algorithm, nodes are randomly assigned indices, and edges are oriented to originate at the node with the lower index and point to the node with the higher index (STAR Methods). Here, for ease in interpreting the structural properties of the network, we re-parameterize the model so that the expected number of regulators (edges) per gene is r and the expected fraction of edges within groups is m (Figures 3A and S5; STAR Methods).

We use the relationships specified by the simulated DAG as the basis for the gene expression model. As in the previous section, the expression, y_g, of gene g is

$$y_g = \underbrace{\sum_{i=1}^{q} x_i \beta_i}_{\text{cis}} + \underbrace{\sum_{j \in \text{par}(g)} y_j \gamma_j}_{\text{trans}}, \qquad \text{(Equation 3)}$$

where par(g) denotes the "parents" of gene g—nodes with edges that point into node g. We again assume that γ_j has fixed magnitude γ, that each gene acts consistently as an activator with probability p^+ or as a repressor with probability $(1 - p^+)$, and that all genes in the GRN have *cis*-eQTLs that collectively contribute unit variance to their expression.

To describe the effects of local and global network properties on expression heritability, we simulated 10,000 synthetic DAGs using the PPM and computed the resulting fraction of *cis*-heritability, h^2_{cis}/h^2, for every gene in the network (STAR Methods). We used the median of h^2_{cis}/h^2 across genes as a summary statistic for each GRN and considered how this median *cis*-heritability fraction changes as a function of the parameters of the PPM and the gene expression model.

As was the case for a single gene, strong local regulation reduced the fraction of *cis*-acting heritability across networks. This was true both for the number of regulators r and their strength γ—in particular, h^2_{cis}/h^2 was tightly related to $1/(1 + r\gamma^2)$ (Figure S6). After regressing out this direct effect among networks with sufficiently strong *trans*-regulation ($1/(1 + r\gamma^2) > 0.6$), the other parameters had more obvious effects: the fraction of genes that are activators, p^+, increased contributions from *trans*-eQTLs and scaled the contribution of triangle and diamond motifs to *trans*-acting variance (Figure S6).

Further, group structure also had an important effect on the magnitude of indirect *trans*-acting genetic variance. Similar to motifs, the number of groups, k, and their modularity, m, interacted with the fraction of activators, p^+, to explain some residual variance in the *cis*-heritability fraction. In networks with many activators (p^+ near 1), modularity amplified *trans*-effects, but in networks with many repressors, modularity dampened them (Figure 3B). Since m is strongly related to the number of triangle and diamond motifs in the network (Figure S7), we conducted a mediation analysis to consider the extent to which these motifs explain away the effects of group structure. In these networks, nearly all of the statistical variance explained by group structure (k and m, scaled by $(2p^+ - 1)$) is explained away by triangle and diamond motifs (Figures 3C and 3D; model R^2 = 0.177,0.260 without regressing out motifs and model R^2 = 0.010,0.020 when regressing out motifs for groups and modularity, respectively). Thus, modular groupings altered global patterns of expression heritability in the network by introducing local structures.

Regulatory hubs

In biological networks across species, regulatory activity is thought to concentrate at hubs with downstream effects on modular groups.[16,31] We therefore expect GRNs to have heavy-tailed out-degree distributions. To assess how hubs affect *cis*- and *trans*-heritability, we modified a previously described directed scale-free network-generating algorithm to produce acyclic graphs (STAR Methods).[32,33] The parameters of this algorithm are interpreted identically to the PPM but with an additional out-degree uniformity term, d. Smaller values of d produce networks with a heavy-tailed out-degree distribution, while larger values of d produce networks with a less hub-like regulatory architecture (Figures 4A and S8).

In networks generated using this model, the effects of both local and global regulatory properties (number and strength of regulators, number of triangle and diamond motifs, and modular

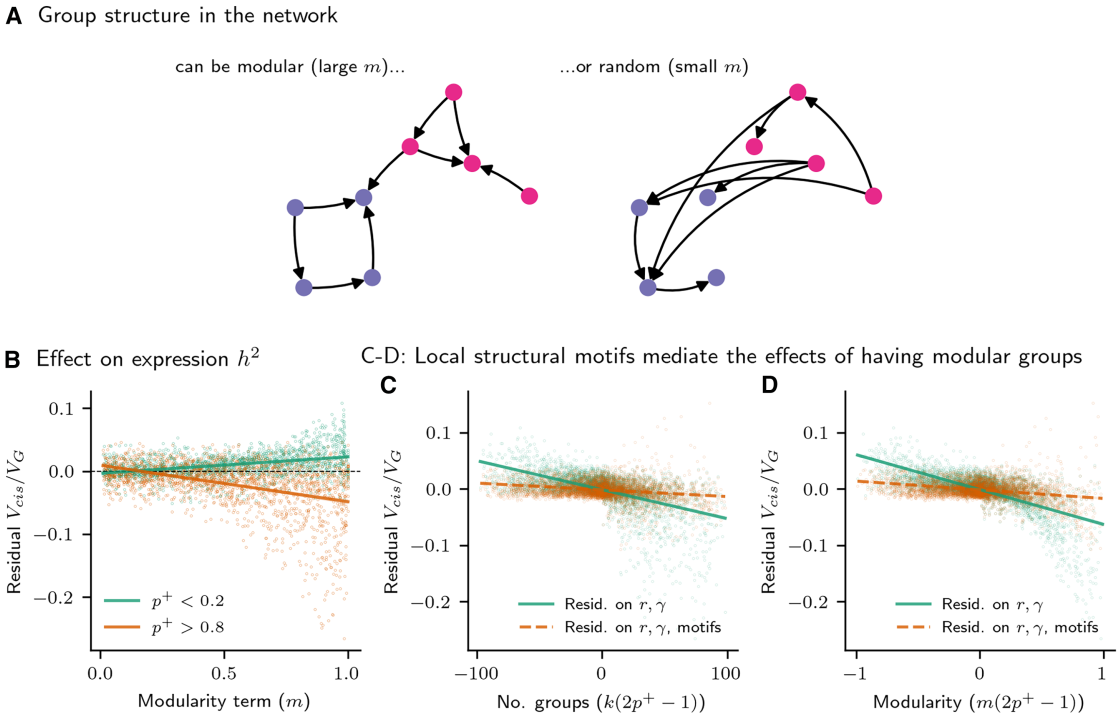

Figure 3. Modular network structure

(A) Toy example of a GRN with modular groups that account for most edges in the network (large m) or groups that are random with respect to edges (small m).

(B) The effect of modularity (m) on the median fraction of *cis*-acting genetic variance, residualized on direct effects from the number of regulators (r) and the strength of regulation (γ). The effect depends on the fraction of genes in the network that are activators in a subset of 10,000 GRNs simulated using the planted partition model (PPM; STAR Methods).

(C and D) Structural motifs explain away the effect of the number of groups (k; C) and modularity (m; D) when appropriately scaled by the expected sign of regulation ($2p^+ - 1$)—residualizing the median fraction of *cis*-acting genetic variance in the GRN by the number of triangle and diamond motifs nearly eliminates the relationship with the scaled group structure terms.

See also Figures S5–S7.

group structures) were consistent with results from the PPM model. Namely, direct regulatory effects had the largest impact on the *cis*-acting fraction of heritability (in particular $1/(1 + r\gamma^2)$, which we again filter and regress out for downstream analysis; Figure S9). Furthermore, the fraction of genes that are activators, p^+, was still a significant driver of *trans*-acting heritability and retained significant interactions with other structural properties of the GRN (Figure S9).

We also found that out-degree dispersion reduced the *cis*-fraction of expression heritability in a manner that does not depend on p^+ (Figure 4B). Further, this effect was not mediated by the number of motifs in the network (Figure 4E), although motifs were important mediators of the effects of modular groups (Figures 4C and 4D). Rather, hub-like regulatory architecture decreased *trans*-acting heritability even though these networks were motif rich compared to networks with a dispersed regulatory architecture (unless the network was dense and had many modular groups; Figure S10). Instead, introducing hubs into the architecture of the network (i.e., lowering d) resulted in fewer long paths through the GRN, which lowered the number of (total) regulators per gene and meant that regulators closer to genes explained more *trans*-heritability (Figure S11).

Network distribution of heritability

We have so far considered how network properties alter the distribution of expression heritability for the median gene in the GRN. But this raises a key question: which of these properties, if any, are necessary to explain the observed distribution of *cis*- and *trans*-heritability?

To address this question, we returned to our motivating dataset.[7] Inspired by approaches from approximate Bayesian computation (ABC),[34–36] we compared synthetic networks generated using the PPM and scale-free network models to the observed distribution of *cis*-heritability fraction across genes in these data and evaluated characteristics of the 250 GRNs that are closest to it using the Kolmogorov-Smirnov test (STAR Methods).

Overall, we found that the observed distribution of *cis*-heritability was well matched by some networks generated by the scale-free network-generating algorithm (Figure 5A), while networks from the PPM tended to be less similar (Figure S12). Since we did not assume a particular distribution of *cis*-eQTL effect sizes for the synthetic GRNs, we compared the contribution of the largest *trans*-regulator to the *cis*-acting variance for each gene as a proxy for *cis*- and *trans*-eQTL effect sizes. This corresponds to the assumption that the lead *cis*-eQTL effects are

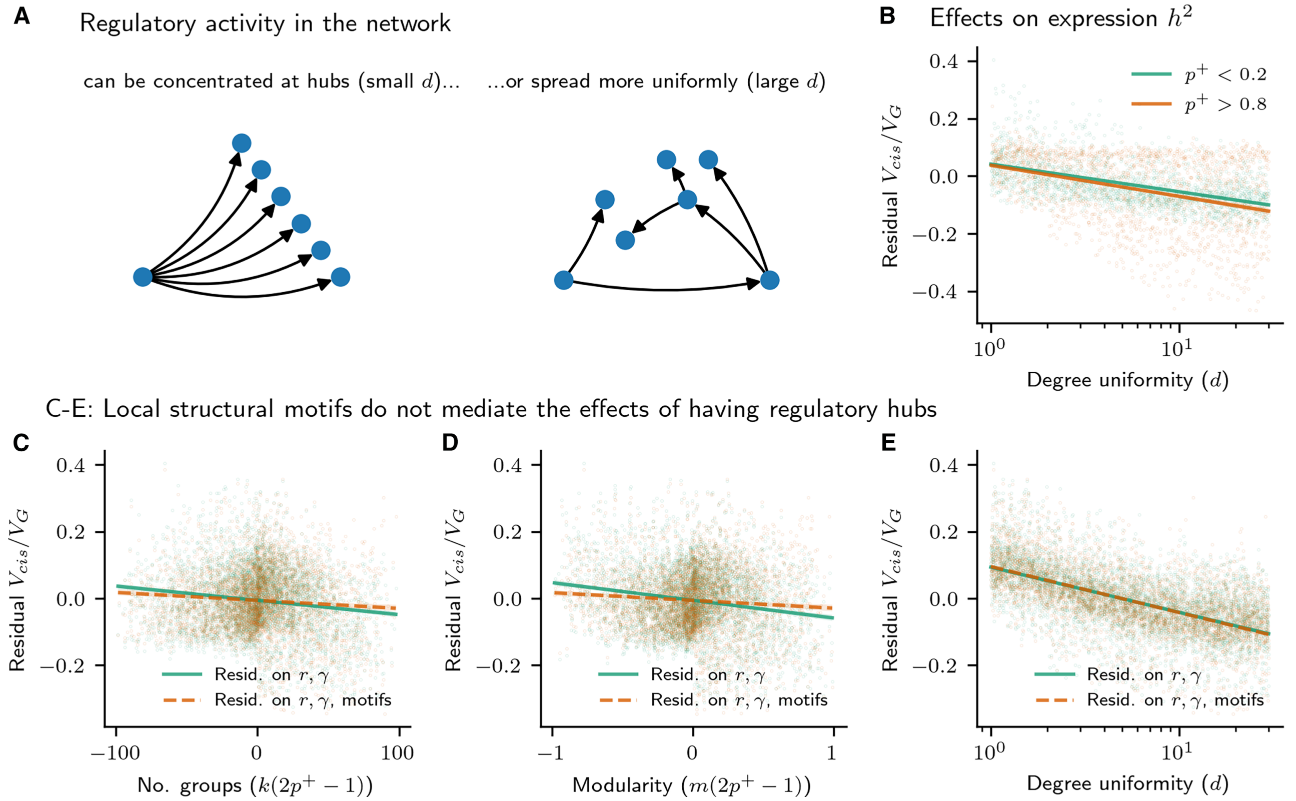

Figure 4. Regulatory hubs in the GRN

(A) Toy example of a GRN with hub regulators (small d) or with a more uniformly spread regulatory architecture (large d).

(B) The effect of degree uniformity, d, on the median fraction of *cis*-acting genetic variance, residualized on direct effects from the number of regulators (r) and the strength of regulation (γ). The effect is no longer on the fraction of genes in the network that are activators in a subset of 10,000 GRNs simulated using our generating algorithm (STAR Methods).

(C–E) Structural motifs explain away the effect of the number of groups, k (C), and modularity, m (D), when appropriately scaled by the expected sign of regulation ($2p^+ - 1$) but not the effect of degree uniformity, d (E). Further residualizing the median fraction of *cis*-acting genetic variance in the GRN by the number of triangle and diamond motifs nearly eliminates the relationship with the scaled group structure terms but has no effect on the relationship with degree uniformity.

See also Figures S8–S11.

similarly distributed for all genes and that all *trans*-eQTLs are also *cis*-eQTLs. Even without explicitly matching simulated networks on the distribution of *trans*-eQTL effect sizes, we found that the simulated GRNs that were well matched to the observed distribution of *cis*-heritability fraction also tended to match the observed ratio of median lead *trans*- to *cis*-eQTL effect sizes (median of 0.51 in matched GRNs; Figure 5B).

Further, we found that the GRNs matched to real data share characteristic properties. First, while r and γ vary in the set of well-matched networks, the quantity $1/(1 + r\gamma^2)$ is tightly constrained, indicating that the distribution of *cis*-acting heritability is highly informative about the strength of direct regulation (Figure 5C). Similarly, the fraction of activators p^+ tends to be at the high end of its range, mirroring the empirical observation that activation is a more common regulatory mechanism than repression (Figure 5D).[29] Further, when p^+ is large in our model, motifs such as the feedforward loop are more likely to be coherent than incoherent. This would run counter to the view that incoherent feedforward loops are key motifs in biological networks,[29] but it may be instead that these motifs are structured (rather than random) with respect to the sign of each regulator in real GRNs.

Finally, the well-matched GRNs tend to be modular and have hub regulators, as evidenced by a minor enrichment of m near 1 and d at the low end of its range (Figures 5E and 5F). These parameters, as with many terms in our model, have largely independent effects on how well GRNs match real data (Figure S14). However, after adjusting for the effects of the modularity and degree parameters (m and d), we find only weak statistical evidence for an enrichment of structural motifs among matched networks (triangles and diamonds; Figure S13).

These results broadly replicate across cohorts and data types. First, we find a similar distribution of heritability across genes in the network when resampling parameters of the expression model in each of these three example GRNs (i.e., taking $|\gamma|$ as independent normal random variables for each gene; see Figure S19). Second, our enrichment analysis of matched network structures broadly replicates in two family cohort studies of gene expression in adipose and whole blood tissue samples.[4] The median *cis*-contribution to gene expression

CellPress
OPEN ACCESS

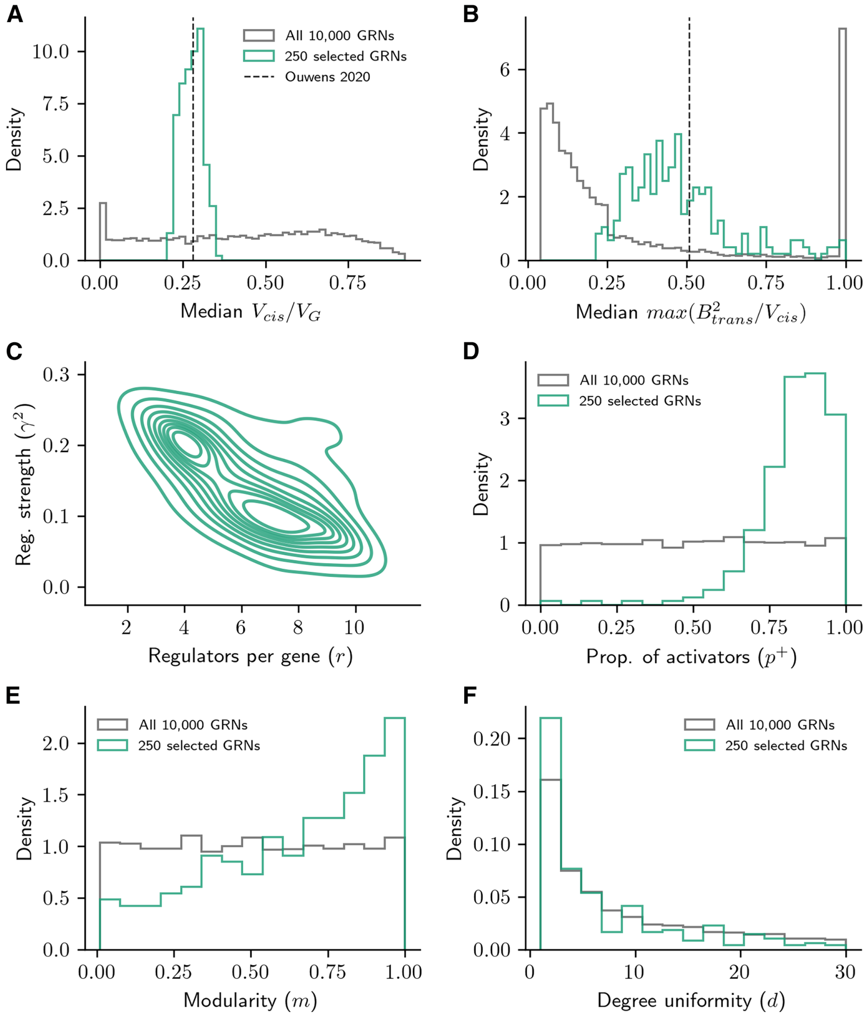

Figure 5. Relationship to real data

(A and B) Median fraction of *cis*-acting expression variance (A) and scaled *trans*-acting effects (B) in all 10,000 scale-free GRNs and in the 250 GRNs most closely matched to the cumulative distribution of h^2_{cis}/h^2 from real data.

(C–F) Distribution of local regulatory parameters (r and γ) (C), fraction of activators (p^+) (D), and structural parameters for modularity (m; E) and degree uniformity (d; F) in the matched networks against the background distribution of GRN parameters.

See also Figures S12–S16.

heritability is different in these studies compared to our motivating dataset (median h^2_{cis}/h^2_{trans} = 0.39 in adipose and 0.44 in whole blood), which shifts estimates of regulatory sparsity and strength (r and $r\gamma^2$) but not terms for global network topology (m and d; Figures S15 and S16).

Further, in previous work, we showed that experimental perturbation data also suggested that GRNs are sparse and modular and have hub regulators.[33] On balance, the effects of these parameters in this setting were to dampen the effects of a random perturbation on the expression state of the network.[33] Here, we find that these same properties are also associated with weaker *trans*-eQTL effects in aggregate (as measured by the fraction of *cis*-acting heritability; Figure S13). Thus, these two distinct modeling frameworks, matched to two distinct types of data, both indicate that sparsity, modularity, and hub regulators are important structural properties of GRNs that coherently affect key measures of their function.

Implications for discovery

Finally, we consider the implications of these characteristic properties of GRNs for the genetic architecture of gene expression. For this, we compared how expression heritability is distributed throughout the network in three exemplar GRNs that have diverse structural features—one GRN with realistic scale-free structure and that closely matches summaries of the twin study estimates of expression heritability, another GRN with similar properties to this network but lacking regulatory hubs (large d), and a third GRN lacking modular structure but with a similar number of edges (PPM). Although we hand-selected these networks to be similarly sparse ($r \approx 8$) and have a median $h^2_{\text{cis}}/h^2 \approx 0.28$ as in the real eQTL data, their distributions of *cis*-heritability fraction are otherwise quite different, and only the first GRN matched the full distribution in the real data (Figure 6A).

These GRNs were further differentiated when considering contributions to heritability beyond *cis* and *trans*. For this, we decomposed expression variance as a function of distance in the GRN to a focal gene. In the well-matched scale-free GRN, h^2_{trans} was modestly more attributable to regulators closer to their target genes in the GRN (i.e., at distance 1 or 2 than at distance 3 or 4; Figures 6B, S17, and S18). Regulators within two hops of a focal gene (e.g., those as diagrammed in Figure 2) cumulatively explained 91.2% of heritability for the median gene in the well-matched GRN, compared to 84.3% or 86.6% in the GRNs without hubs or modular groups. It has similarly been observed on the yeast transcriptional network, which has a topology similar to our synthetic GRNs,[37,38] that most genetic variance in gene expression can be attributed to regulatory connections within two or three hops of a focal gene.[27]

This concentration of expression heritability near a given gene in the well-matched GRN occurred despite the typical gene in it having fewer regulators (Figures 6C and 6D). Though genes in all three GRNs were expected to have a similar number of direct regulators (r = 7.93, 7.93, and 8.04 across the three GRNs), their varied structural properties dramatically altered the architecture of indirect regulation. Genes near the top of the PPM DAG had tens (or fewer) of upstream regulators, but genes near the bottom

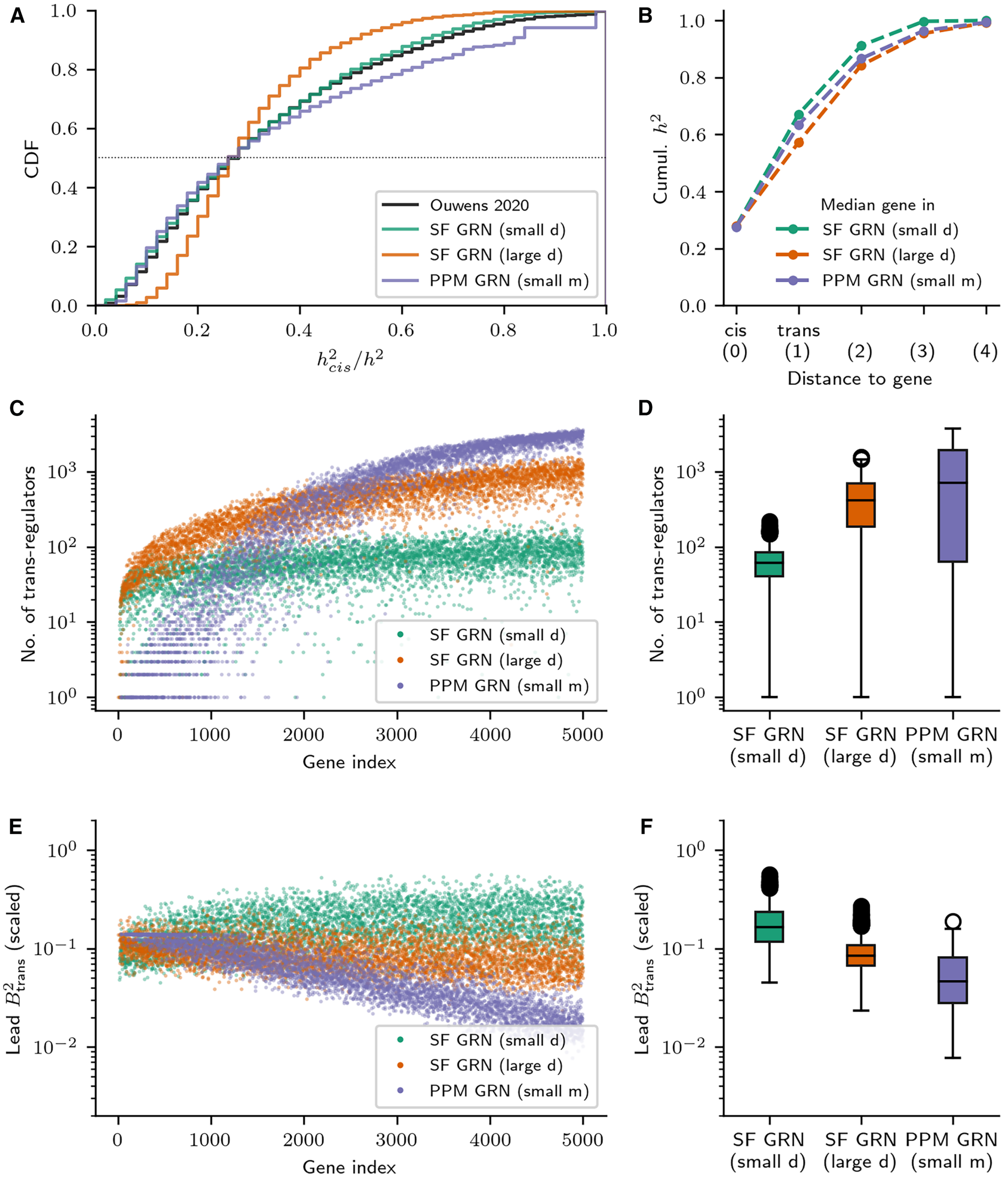

Figure 6. Genetic architecture in diverse GRNs

(A) Cumulative distribution of the fraction of *cis*-acting expression variance in three example GRNs with different structural properties against the distribution from real data.[7] Two GRNs were generated with our directed scale-free algorithm (SF), one with many hub regulators (small *d*) and one with fewer (large *d*), and one GRN was generated with the planted partition model and has neither hub regulators nor modular groups (PPM, small *m*).

(B) Median cumulative heritability as a function of network distance in three example GRNs. Median is over genes in the network; *cis*-effects are at distance 0, *trans*-effects from direct regulators are at distance 1, etc.

(C and E) Number of *trans*-regulators (direct and indirect) (C) and lead *trans*-eQTL effect sizes (E) for all genes in the example GRNs as a function of a gene's position ("index") in the topological ordering of the underlying DAG.

(D and F) Boxplots of the distributions in (C) and (E). Boxes extend to the interquartile range (IQR) of the distribution, with a line at its median. Whiskers extend to the range of the data, capped at 1.5 times the IQR.

See also Figures S17–S19.

sometimes had thousands (Figure 6C)—meanwhile, the number of regulators of genes in both scale-free GRNs tended to plateau toward the bottom of the DAG (Figure 6C). Similarly, a gene in the scale-free GRN with hubs typically had substantially fewer upstream regulators (median 61) than genes in either other GRN (median of 413 in the scale-free GRN without hubs and 589 in the PPM GRN; Figure 6D). We further measured this difference in indirect regulatory architecture by decomposing heritability as a function of distance between regulators and target genes in each GRN; the fraction of variance explained by regulators at various distances also stabilized toward the bottom of the DAG in the scale-free GRNs but not the PPM GRN (Figure S17).

These exemplar GRNs also had different distributions of lead *trans*-eQTL effect sizes across genes in the network (Figures 6E and 6F). As in real eQTL studies, we computed these effects in units of expression standard deviation, separately for each gene in the network. We found that across networks, genes with more upstream regulators typically had smaller (scaled) leading *trans*-eQTL effects, as each regulator contributed a smaller proportion of variance. This was particularly true for genes near the bottom of the PPM GRN (Figures 6C and 6E). Meanwhile, in the comparatively motif-rich scale-free networks, where the number of upstream regulators grew more slowly as a function of gene index (Figure 6C), the distribution of *trans*-eQTL effect sizes was more uniform across genes (Figures 6E and 6F).

Overall, lead *trans*-eQTL effects were largest in the GRN that was well matched to real data and smaller in the matched GRNs that had a similar median h^2_{cis} but lacked hub regulators and modular groups (Figure 6F). We found this to be somewhat counterintuitive, as low out-degree uniformity (small d) and high modularity (large m) were marginally associated with lower *trans*-acting variance (i.e., with higher median h^2_{cis}/h^2; Figure S9). However, the median h^2_{cis}/h^2 of genes in a GRN does not specify the full distribution of h^2_{cis}/h^2 (Figure 6A), nor does it specify the underlying architecture of *trans*-effects (Figure 6F). The distribution of *trans*-eQTL effect sizes depends on the number and nature of paths between a gene's upstream regulators, which is dramatically altered by modular group structure or hub regulatory architecture. Taken together, these network properties suggest that *trans*-acting effects should be larger and closer to focal genes than would be naively expected under an unstructured GRN model with low *cis*-heritability, with fewer loci contributing *trans*-acting variance.

DISCUSSION

In this work, we have characterized the space of GRNs that are compatible with a simple observation about the genetic architecture of gene expression: namely, that h^2_{cis} is generally low despite β^2_{trans} usually being small. To do this, we evaluated the effects of various structural properties of GRNs and their necessity (or lack thereof) to match patterns in real data. Our results illuminate what eQTLs can tell us about incompletely mapped GRNs, but we have not yet discovered what the space of plausible GRNs can tell us about the eQTLs.

Across networks, we found that direct regulatory effects (i.e., the number of regulators per gene and the average strength of regulation) have a key role in determining the relative sizes of *trans*-eQTL effects and the proportion of *trans*-acting heritability. However, peripheral effects due to the organization of distal regulators were also important for matching the observed genetic architecture of genome-wide gene expression. Specifically, local structural motifs mediate a substantial portion of the effects of group structure in the network, and regulatory hubs shift *trans*-eQTL effects closer to genes and toward key loci in the network.

We acknowledge several limitations of this study. While our model describes key topological features of GRNs, it also makes simplifying assumptions about the functional form of gene expression and the activity of regulators throughout the GRN. In reality, gene expression is a dynamic, time-varying process with non-linearities due to molecular kinematics and protein interactions; regulatory effects are not all equal in magnitude, and regulators do not necessarily act with consistent sign, nor are local motifs random with respect to this direction of effect; and finally, not all genes have equal *cis*-acting variance, and the distribution of *cis*-eQTL effects is of further interest in the context of complex trait genetic architecture. While many of these parameters are also often abstracted away in quantitative genetics models (e.g., the infinitesimal model is agnostic to regulatory architecture), they are important aspects of GRNs and their function across time and tissues. Relatedly, our work does not resolve questions about eQTLs whose effects may only be visible over dynamic or developmental trajectories or in narrower populations of single cells in bulk tissues. The question of why some genes lack *cis*-eQTL variance in certain contexts also remains open for further study.

Still, it remains that the structural properties of GRNs exert a significant influence on the distribution of genetic effects on gene expression. In particular, synthetic GRNs with structural features characteristic of biological networks (i.e., sparsity, modularity, and a heavy-tailed out-degree distribution) were categorically better matched to real data than GRNs without them, as we previously observed with experimental perturbation data from a human erythroid progenitor cell line.[33] We therefore anticipate that structure learning should be biased toward GRNs having (1) a single-digit number of regulators per gene; (2) more activators than repressors; (3) an inverse relationship between graph density and regulatory strength; (4) modularity, with respect to some gene sets; and (5) a heavy-tailed out-degree distribution. These inductive biases can be introduced through model architecture, loss function, and the use of prior knowledge from the biomedical literature.

Our results further suggest that the architecture of gene expression is sparser than would be anticipated under unstructured models of GRNs. Recent work using genome-scale Perturb-seq has found that most regulators influence the expression of a small number of downstream genes in the cell (fewer than 50 for the median gene and 500 or more for the typical essential gene[39,40]). We find this to be consistent with our result that the median gene in a realistic synthetic GRN has tens of upstream regulators that contribute variance in its expression, as opposed to hundreds of such regulators in GRNs with less realistic topologies (Figure 6D).

Relatedly, we found that *trans*-eQTLs in realistic GRNs tend to cluster at key loci that have pleiotropic effects within modules. These results support the use of statistical aggregation tests

that exploit these features of GRNs to further map *trans*-acting genetic effects on gene expression. To the extent that this co-regulation reflects functional relatedness, we further anticipate that using this genetic signal to define gene ''programs'' will be a key way forward to uncover the biology of complex traits and diseases.

RESOURCE AVAILABILITY

Lead contact

Requests for further information and resources should be directed to and will be fulfilled by the lead contact, Matthew Aguirre (magu@stanford.edu).

Materials availability

This study did not generate new unique reagents.

Data and code availability

All original code used to perform simulations and analysis can be found on GitHub at https://doi.org/10.5281/zenodo.18893866. Summary statistics for networks generated in this study can be found at https://doi.org/10.5281/zenodo.19024416. Any additional information required to reanalyze the data reported in this paper is available from the lead contact upon request.

ACKNOWLEDGMENTS

We would like to thank members of the Pritchard Lab at Stanford University for helpful comments and discussion related to this work. M.A. acknowledges support from a Microsoft Research PhD Fellowship and from the National Library of Medicine (NLM) under training grant T15LM007033. This work was supported by the National Human Genome Research Institute under grants R01HG008140, R01HG014005, and U01HG012069 (J.K.P.) and by the National Institute of General Medical Sciences under grant R01GM115889 (G.S.).

AUTHOR CONTRIBUTIONS

Data curation, formal analysis, investigation, software, validation, and visualization, M.A.; resources, J.K.P.; funding acquisition, M.A., G.S., and J.K.P.; supervision, J.P.S., G.S., and J.K.P.; conceptualization, methodology, project administration, writing – original draft, and writing – review & editing, all authors.

DECLARATION OF INTERESTS

The authors declare no competing interests.

STAR★METHODS

Detailed methods are provided in the online version of this paper and include the following:

- KEY RESOURCES TABLE
- METHOD DETAILS
  - Empirical eQTL data
  - Gene expression model
  - Planted partition model
  - Choosing hyperparameters
  - Modular directed acyclic scale-free graph
  - Choosing hyperparameters
  - Expression variance decomposition
- QUANTIFICATION AND STATISTICAL ANALYSIS
  - Real data comparison

SUPPLEMENTAL INFORMATION

Supplemental information can be found online at https://doi.org/10.1016/j.xgen.2026.101219.

Received: August 15, 2025
Revised: January 11, 2026
Accepted: March 27, 2026
Published: April 22, 2026

REFERENCES

1. Maurano, M.T., Humbert, R., Rynes, E., Thurman, R.E., Haugen, E., Wang, H., Reynolds, A.P., Sandstrom, R., Qu, H., Brody, J., et al. (2012). Systematic Localization of Common Disease-Associated Variation in Regulatory DNA. Science *337*, 1190–1195. https://doi.org/10.1126/science.1222794.
2. Watanabe, K., Stringer, S., Frei, O., Umićević Mirkov, M., de Leeuw, C., Polderman, T.J.C., van der Sluis, S., Andreassen, O.A., Neale, B.M., and Posthuma, D. (2019). A global overview of pleiotropy and genetic architecture in complex traits. Nat. Genet. *51*, 1339–1348. https://doi.org/10.1038/s41588-019-0481-0.
3. GTEx Consortium (2020). The GTEx Consortium atlas of genetic regulatory effects across human tissues. Science *369*, 1318–1330. https://doi.org/10.1126/science.aaz1776.
4. Price, A.L., Helgason, A., Thorleifsson, G., McCarroll, S.A., Kong, A., and Stefansson, K. (2011). Single-Tissue and Cross-Tissue Heritability of Gene Expression Via Identity-by-Descent in Related or Unrelated Individuals. PLoS Genet. *7*, e1001317. https://doi.org/10.1371/journal.pgen.1001317.
5. Boyle, E.A., Li, Y.I., and Pritchard, J.K. (2017). An Expanded View of Complex Traits: From Polygenic to Omnigenic. Cell *169*, 1177–1186. https://doi.org/10.1016/j.cell.2017.05.038.
6. Liu, X., Li, Y.I., and Pritchard, J.K. (2019). Trans Effects on Gene Expression Can Drive Omnigenic Inheritance. Cell *177*, 1022–1034.e6. https://doi.org/10.1016/j.cell.2019.04.014.
7. Ouwens, K.G., Jansen, R., Nivard, M.G., van Dongen, J., Frieser, M.J., Hottenga, J.-J., Arindrarto, W., Claringbould, A., van Iterson, M., Mei, H., et al. (2020). A characterization of cis- and trans-heritability of RNA-Seq-based gene expression. Eur. J. Hum. Genet. *28*, 253–263. https://doi.org/10.1038/s41431-019-0511-5.
8. Yao, D.W., O'Connor, L.J., Price, A.L., and Gusev, A. (2020). Quantifying genetic effects on disease mediated by assayed gene expression levels. Nat. Genet. *52*, 626–633. https://doi.org/10.1038/s41588-020-0625-2.
9. Mostafavi, H., Spence, J.P., Naqvi, S., and Pritchard, J.K. (2023). Systematic differences in discovery of genetic effects on gene expression and complex traits. Nat. Genet. *55*, 1866–1875. https://doi.org/10.1038/s41588-023-01529-1.
10. Giambartolomei, C., Vukcevic, D., Schadt, E.E., Franke, L., Hingorani, A.D., Wallace, C., and Plagnol, V. (2014). Bayesian test for colocalisation between pairs of genetic association studies using summary statistics. PLoS Genet. *10*, e1004383. https://doi.org/10.1371/journal.pgen.1004383.
11. Wallace, C., Rotival, M., Cooper, J. D., Rice, C. M., Yang, J. H. M., McNeill, M., Smyth, D. J., Niblett, D., Cambien, F., Cardiogenics Consortium, et al (2012). Statistical colocalization of monocyte gene expression and genetic risk variants for type 1 diabetes. Hum. Mol. Genet. 21, 2815–2824. doi:https://doi.org/10.1093/hmg/dds098.
12. Gamazon, E.R., Wheeler, H.E., Shah, K.P., Mozaffari, S.V., Aquino-Michaels, K., Carroll, R.J., Eyler, A.E., Denny, J.C., GTEx Consortium; and Nicolae, D.L., et al. (2015). A gene-based association method for mapping traits using reference transcriptome data. Nat. Genet. *47*, 1091–1098. https://doi.org/10.1038/ng.3367.
13. Wainberg, M., Sinnott-Armstrong, N., Mancuso, N., Barbeira, A.N., Knowles, D.A., Golan, D., Ermel, R., Ruusalepp, A., Quertermous, T., Hao, K., et al. (2019). Opportunities and challenges for transcriptome-wide association studies. Nat. Genet. *51*, 592–599. https://doi.org/10.1038/s41588-019-0385-z.
14. Connally, N.J., Nazeen, S., Lee, D., Shi, H., Stamatoyannopoulos, J., Chun, S., Cotsapas, C., Cassa, C.A., and Sunyaev, S.R. (2022). The missing link between genetic association and regulatory function. eLife *11*, e74970. https://doi.org/10.7554/eLife.74970.

15. Umans, B.D., Battle, A., and Gilad, Y. (2021). Where Are the Disease-Associated eQTLs? Trends Genet. *37*, 109–124. https://doi.org/10.1016/j.tig.2020.08.009.

16. Võsa, U., Claringbould, A., Westra, H.-J., Bonder, M.J., Deelen, P., Zeng, B., Kirsten, H., Saha, A., Kreuzhuber, R., Yazar, S., et al. (2021). Large-scale cis- and trans-eQTL analyses identify thousands of genetic loci and polygenic scores that regulate blood gene expression. Nat. Genet. *53*, 1300–1310. https://doi.org/10.1038/s41588-021-00913-z.

17. Yazar, S., Alquicira-Hernandez, J., Wing, K., Senabouth, A., Gordon, M.G., Andersen, S., Lu, Q., Rowson, A., Taylor, T.R.P., Clarke, L., et al. (2022). Single-cell eQTL mapping identifies cell type–specific genetic control of autoimmune disease. Science *376*, eabf3041. https://doi.org/10.1126/science.abf3041.

18. van der Wijst, M., de Vries, D.H., Groot, H.E., Trynka, G., Hon, C.C., Bonder, M.J., Stegle, O., Nawijn, M.C., Idaghdour, Y., van der Harst, P., et al. (2020). The single-cell eQTLGen consortium. eLife *9*, e52155. https://doi.org/10.7554/eLife.52155.

19. Coorens, T.H.H., Guillaumet-Adkins, A., Kovner, R., Linn, R.L., Roberts, V.H.J., Sule, A., and Van Hoose, P.M.; dGTEx Consortium (2025). The human and non-human primate developmental GTEx projects. Nature *637*, 557–564. https://doi.org/10.1038/s41586-024-08244-9.

20. Cuomo, A.S.E., Spenceley, E., Tanudisastro, H.A., Bowen, B., Henry, A., Huang, H.L., Xue, A., Zhou, W., Welland, M.J., Lee, A.S., et al. (2025). Impact of Rare and Common Genetic Variation on Cell Type-Specific Gene Expression. Preprint at medRxiv. https://doi.org/10.1101/2025.03.20.25324352.

21. Wang, L., Babushkin, N., Liu, Z., and Liu, X. (2024). Trans-eQTL mapping in gene sets identifies network effects of genetic variants. Cell Genom. *4*, 100538. https://doi.org/10.1016/j.xgen.2024.100538.

22. Qi, G., Lila, E., Ji, Z., Shojaie, A., Battle, A., and Sun, W. (2025). Transcriptome-wide association studies at cell state level using single-cell eQTL data. Preprint at medRxiv. https://doi.org/10.1101/2025.03.17.25324128.

23. Zhang, Z.E., Kim, A., Suboc, N., Mancuso, N., and Gazal, S. (2025). Efficient count-based models improve power and robustness for large-scale single-cell eQTL mapping. Preprint at medRxiv. https://doi.org/10.1101/2025.01.18.25320755.

24. Yang, F., Wang, J., GTEx Consortium; Pierce, B.L., Chen, L.S., Aguet, F., Ardlie, K.G., Cummings, B.B., Gelfand, E.T., Getz, G., et al. (2017). Identifying cis-mediators for trans-eQTLs across many human tissues using genomic mediation analysis. Genome Res. *27*, 1859–1871. https://doi.org/10.1101/gr.216754.116.

25. Dong, Z., Song, T., and Yuan, C. (2013). Inference of Gene Regulatory Networks from Genetic Perturbations with Linear Regression Model. PLoS One *8*, e83263. https://doi.org/10.1371/journal.pone.0083263.

26. Cai, X., Bazerque, J.A., and Giannakis, G.B. (2013). Inference of Gene Regulatory Networks with Sparse Structural Equation Models Exploiting Genetic Perturbations. PLoS Comput. Biol. *9*, e1003068. https://doi.org/10.1371/journal.pcbi.1003068.

27. Ružičková, N., Hledík, M., and Tkačik, G. (2024). Quantitative omnigenic model discovers interpretable genome-wide associations. Proc. Natl. Acad. Sci. USA *121*, e2402340121. https://doi.org/10.1073/pnas.2402340121.

28. Milo, R., Shen-Orr, S., Itzkovitz, S., Kashtan, N., Chklovskii, D., and Alon, U. (2002). Network Motifs: Simple Building Blocks of Complex Networks. Science *298*, 824–827. https://doi.org/10.1126/science.298.5594.824.

29. Alon, U. (2007). Network motifs: theory and experimental approaches. Nat. Rev. Genet. *8*, 450–461. https://doi.org/10.1038/nrg2102.

30. Condon, A., and Karp, R.M. (2001). Algorithms for graph partitioning on the planted partition model. Random Struct. Algorithm *18*, 116–140. https://doi.org/10.1002/1098-2418(200103)18:2<116::AID-RSA1001>3.0.CO;2-2.

31. Albert, F.W., Bloom, J.S., Siegel, J., Day, L., and Kruglyak, L. (2018). Genetics of trans-regulatory variation in gene expression. eLife *7*, e35471. https://doi.org/10.7554/eLife.35471.

32. Bollobas, B., Borgs, C., Chayes, J., and Riordan, O. (2003). Directed Scale-Free Graphs. SODA *3*, 132–139.

33. Aguirre, M., Spence, J.P., Sella, G., and Pritchard, J.K. (2025). Gene regulatory network structure informs the distribution of perturbation effects. PLoS Comput. Biol. *21*, e1013387. https://doi.org/10.1371/journal.pcbi.1013387.

34. Tavaré, S., Balding, D.J., Griffiths, R.C., and Donnelly, P. (1997). Inferring Coalescence Times From DNA Sequence Data. Genetics *145*, 505–518. https://doi.org/10.1093/genetics/145.2.505.

35. Beaumont, M.A., Zhang, W., and Balding, D.J. (2002). Approximate Bayesian Computation in Population Genetics. Genetics *162*, 2025–2035. https://doi.org/10.1093/genetics/162.4.2025.

36. Craiu, R.V., and Levi, E. (2023). Approximate Methods for Bayesian Computation. Annu. Rev. Stat. Appl. *10*, 379–399. https://doi.org/10.1146/annurev-statistics-033121-110254.

37. Monteiro, P.T., Oliveira, J., Pais, P., Antunes, M., Palma, M., Cavalheiro, M., Galocha, M., Godinho, C.P., Martins, L.C., Bourbon, N., et al. (2020). YEASTRACT+: a portal for cross-species comparative genomics of transcription regulation in yeasts. Nucleic Acids Res. *48*, D642–D649. https://doi.org/10.1093/nar/gkz859.

38. Monteiro, P.T., Pedreira, T., Galocha, M., Teixeira, M.C., and Chaouiya, C. (2020). Assessing regulatory features of the current transcriptional network of Saccharomyces cerevisiae. Sci. Rep. *10*, 17744. https://doi.org/10.1038/s41598-020-74043-7.

39. Nadig, A., Replogle, J.M., Pogson, A.N., Murthy, M., McCarroll, S.A., Weissman, J.S., Robinson, E.B., and O'Connor, L.J. (2025). Transcriptome-wide analysis of differential expression in perturbation atlases. Nat. Genet. *57*, 1228–1237. https://doi.org/10.1038/s41588-025-02169-3.

40. Zhu, R., Dann, E., Yan, J., Retana, J.R., Goto, R., Guitche, R.C., Petersen, L.K., Ota, M., Pritchard, J.K., and Marson, A. (2025). Genome-scale perturb-seq in primary human CD4+ T cells maps context-specific regulators of T cell programs and human immune traits. Preprint at bioRxiv. https://doi.org/10.64898/2025.12.23.696273.

41. Yang, J., Lee, S.H., Goddard, M.E., and Visscher, P.M. (2011). GCTA: A Tool for Genome-wide Complex Trait Analysis. Am. J. Hum. Genet. *88*, 76–82. https://doi.org/10.1016/j.ajhg.2010.11.011.

42. Erdös, P., and Rényi, A. (1960). On the evolution of random graphs. In The Structure and Dynamics of Networks (Princeton University Press), pp. 38–82. https://doi.org/10.1515/9781400841356.38.

STAR★METHODS

KEY RESOURCES TABLE

REAGENT or RESOURCE	SOURCE	IDENTIFIER
Deposited data		
Blood gene expression summary statistics	Ouwens et al.[7]	https://doi.org/10.1038/s41431-019-0511-5
Blood and adipose gene expression summary statistics	Price et al.[4]	https://doi.org/10.1371/journal.pgen.1001317
Software and algorithms		
Python	Python software foundation	https://www.python.org/
GRN simulation and analysis code	This work	https://doi.org/10.5281/zenodo.18893866
GRN summary statistics	This work	https://doi.org/10.5281/zenodo.19024417

METHOD DETAILS

Empirical eQTL data

In our primary analysis, we make use of data from a study of whole blood gene expression in 1,497 individuals from 709 twin pairs (459 monozygotic and 150 dizygotic).[7] Study design and data collection procedures are described in the initial publication. Briefly, these data consist of measurements of 52,844 genes, which were subsequently filtered for being protein coding, having read counts above zero in at least 85% of samples in each zygosity group (i.e., expressed in in at least 780 MZ twins and 255 DZ twins), a median expression count above 10, and more than 20 SNPs in the *cis*-window, resulting in an analysis of 11,409 genes. Heritability was estimated using the software tool GCTA,[41] with estimates partitioned into *cis*- and *trans*-acting components by considering variation within a 250 kilobase window flanking the gene body as acting in *cis* (and in *trans* otherwise). These data were used in the format provided in Supplementary Table 2 of the initial publication.[7]

For this work, we computed the *cis*-fraction of expression heritability by dividing the point estimates of *cis*- and *trans*-heritability from GCTA as $h^2_{\mathrm{cis}}/(h^2_{\mathrm{cis}} + h^2_{\mathrm{trans}})$, and further subsetted the data to the 5,092 genes with at least one *cis*- and *trans*-eQTL called in the study. This additional filtration step removes 1,293 genes with estimates of h^2_{cis} or h^2_{trans} which were indistinguishable from zero, and introduces an upward shift in the distribution of *cis*-heritability (but not *trans*-heritability; Figure S2).

For replication, we use summary data from genetic studies of gene expression in two tissues, processed as used in previous work.[4] Briefly, one cohort (IFA) contains measurements of 18,735 transcripts in adipose tissue from 687 individuals, and the other (IFB) contains measurements of 19,099 transcripts in whole blood samples from 531 individuals. We used estimates of *cis*- and *trans*-heritability which were estimated using variance components methods, as provided in Table S1 of the original study. For this study, we computed the *cis*-fraction of expression heritability by dividing the point estimates of h^2_{cis} and h^2_{trans} by their sum, as above, and restricted our analysis to genes which had positive estimates of *cis*-, *trans*-, and total heritability, which left 8,954 transcripts in IFA (adipose) and 6,979 transcripts in IFB (blood).

Gene expression model

We model gene expression using a linear structural equation model using a set of causal gene regulatory relationships specified by a directed acyclic graph (DAG) between n genes. We consider the nature of the DAG and its structural properties in the ensuing sections—to start, we assume that gene i harbors q_i *cis*-acting quantitative trait loci (*cis*-eQTLs; $q_i > 0$) and is influenced by some number of regulators, r_i ($r_i \geq 0$). Denoting the expression of gene i in a randomly chosen individual by y_i, we have that

$$y_i = \underbrace{\sum_{k=1}^{q_i} x_{ki}\beta_{ki}}_{\text{cis}} + \underbrace{\sum_{j=1}^{r_i} y_j\gamma_{ji}}_{\text{trans}} + \underbrace{s_i}_{\text{noise}}, \quad \text{(Equation 4)}$$

where β_{ki} and x_{ki} are respectively the effect of the kth eQTL for gene i and an indicator variable for its genotype; γ_{ji} and y_j are the effect of the jth regulator of gene i and its expression respectively; and s_i is non-genetic noise with zero mean and variance σ_i^2, assumed to be independent of genotypes and uncorrelated across genes.

We further assume that each regulator acts consistently as an activator or repressor and has the same magnitude of effect (i.e., $\gamma_{ji} = p_j\gamma$, with p_j denoting the sign for gene j); and that the variance due to *cis*-acting eQTLs and non-genetic noise in expression are the same for all genes (respectively 1, and σ^2). The variance for gene i is then

$$Var(y_i) = \underbrace{1}_{\text{cis}} + \underbrace{\gamma^2\left(\sum_{j=1}^{r_i} Var(y_j) + 2\sum_{j=1}^{r_i}\sum_{j'<j} p_j p_{j'} Cov(y_j, y_{j'})\right)}_{\text{trans}} + \underbrace{\sigma^2}_{\text{noise}} \quad \text{(Equation 5)}$$

With these assumptions, we see that γ^2 scales the relative contributions of *cis*- and *trans*-acting genetic variation, and σ^2 scales the ratio of genetic and non-genetic variance (i.e., the heritability). Specifically, we have that $h^2 = 1/(1+\sigma^2)$ for all genes in the network, since the *trans*-acting terms above can be expanded in terms of *cis*-acting terms for other genes (i.e., the ratio of incoming genetic and noise terms is the same). We can then write that

$$\frac{h^2_{\text{cis},i}}{h^2_i} = \frac{1}{1+\gamma^2\left(\sum_{j=1}^{r_i} Var_G(y_j) + 2\sum_{j=1}^{r_i}\sum_{j'<j} p_j p_{j'} Cov_G(y_j, y_{j'})\right)} \quad \text{(Equation 6)}$$

with Var_G and Cov_G respectively denoting the genetic variance and genetic covariances of genes j and j'. A complete derivation is in Methods S1.

For the example motifs in Figure 2, we can further take the expectation over the random assignments of genes as activators or repressors (i.e., the signs of the edge weights). In this setting, we have the following equations for the expected *cis*-contribution to variance over the expected total genetic variance:

$$\vee\text{motif}: \mathbb{E}_p[V_{cis}] \Big/ \mathbb{E}_p[V_G] = \frac{1}{1+(r+1)\gamma^2} \quad \text{(Equation 7)}$$

$$\diamond\text{motif}: \mathbb{E}_p[V_{cis}] \Big/ \mathbb{E}_p[V_G] = \frac{1}{1+r\gamma^2+r\gamma^4+2\binom{r}{2}(2p^+-1)^2\gamma^4} \quad \text{(Equation 8)}$$

$$\triangleright\text{ motif}: \mathbb{E}_p[V_{cis}] \Big/ \mathbb{E}_p[V_G] = \frac{1}{1+(r+1)\gamma^2+r\gamma^4+2\binom{r}{2}(2p^+-1)^2\gamma^4+2r(2p^+-1)\gamma^3}. \quad \text{(Equation 9)}$$

where p^+ is the probability that each gene is an activator as opposed to a repressor.

Planted partition model

We use the planted partition model (PPM) to produce the synthetic directed acyclic graphs (DAGs) which form the basis of our simpler GRN model. The PPM is a simple extension of the binomial (Erdos-Renyi/ER) random graph model to include group structure.[30,42] The ER model has two parameters: the number of nodes, n, and the edge probability, p. In this model, all edges are independent and identically distributed Bernoulli random variables with parameter p. The PPM extends this by assigning the n nodes to one of k groups uniformly at random; edges exist between members of the same group with probability p and between members of different groups with probability q.

To produce DAGs using the PPM, we further generate a random topological ordering of the n nodes. Edges are directed from the node earlier in the ordering to the node later in the ordering; this constraint also prevents the creation of cycles.

For consistency in interpreting parameters of the gene expression model, and to account for imposition of acyclicity, we reparameterize the PPM in terms of r, k, and m—respectively, the expected number of regulators per gene, the number of groups in the network, and the expected fraction of edges drawn between nodes belonging to the same group. In terms of the original parameters p and q, we have

$$r = (n-1)\frac{p+q(k-1)}{2k} \quad \text{(Equation 10)}$$

$$m = \frac{p}{p+q(k-1)} \quad \text{(Equation 11)}$$

$$p = \frac{2krm}{(n-1)} \quad \text{(Equation 12)}$$

$$q = \frac{2kr(1-m)}{(k-1)(n-1)}. \quad \text{(Equation 13)}$$

We note that there is degeneracy in the above if $k = 1$; in this case, we have recovered the ER model. Further, in the typical PPM formulation, $p<q$ means that the network groups are *dissociative* (i.e., nodes tend to be connected with members of other groups rather than their own); with the above parameters, this occurs when $m < 1/k$. This case is not of interest here, and so we enforce $m > 1/k$ for simulations.

The output of the PPM is a DAG with n genes assigned into k groups. With the above parameters, the groups account for roughly m percent of the edges in the network, and the typical gene has r regulators (Figure S5). To simulate gene expression from this network, we fix the strength of regulation at γ and independently assign each gene a regulatory sign, which is positive with probability p^+. Taking G to be the weighted adjacency matrix after this process, we have that the fraction of *cis*-acting genetic variance for the i'th gene in the GRN is

$$\frac{V_{\mathrm{cis},i}}{V_{\mathrm{G},i}} = \frac{L_{ii}^2}{(L^\top L)_{ii}}, L = (I - G)^{-1} \quad \text{(Equation 14)}$$

where $(X)_{ii}$ is the ith entry on the diagonal of the matrix X and I is the n-dimensional identity matrix. A complete derivation of this result is in Methods S1.

Choosing hyperparameters

To produce the example distribution of heritability in Figure 1, we simulated 50 GRNs with DAG structures produced by the PPM using generating parameters as given below.

- Number of genes $n = 1000$
- Number of groups $k = 1$
- Modularity term m is not applicable in this case (these are ER graphs)
- Expected regulators per gene $r \sim \mathrm{Uniform}(4, 8)$
- Strength of regulation $\gamma \sim \mathrm{Uniform}(0.1, 0.5)$
- Expected fraction of activators $p^+ = 0.5$

In our investigation of the effects of modular network structure on the distribution of heritability, we simulated 10,000 PPM GRNs with generating parameters as given below.

- Number of genes $n = 5000$
- Number of groups $k \sim \mathrm{Uniform}(2, 100)$
- Modularity term $m \sim \mathrm{Uniform}(1/k, 1)$
- Expected regulators per gene $r \sim \mathrm{Uniform}(2, 10)$
- Strength of regulation $\gamma \sim \mathrm{Uniform}(0.2, 0.5)$
- Expected fraction of activators $p^+ \sim \mathrm{Uniform}(0, 1)$

Modular directed acyclic scale-free graph

We use a modular scale-free graph generating algorithm to produce the synthetic DAGs for our full GRN model. This algorithm is an extension of our previous work, which we used to study experimental perturbations; the prior work is itself an extension of a directed scale-free network generating algorithm due to Bollobas.[32,33] The algorithm we use here has an out-degree uniformity parameter d, and otherwise has parameters which are identical in interpretation to our re-parameterized PPM.

The output of this algorithm is a DAG with n nodes assigned into k groups. As with the PPM, the groups account for m percent of the edges in the graph, and the typical node has r neighbors—and further, the out-degree dispersion parameter d controls the spread of the out-degree distribution (Figure S8). When d is near zero, the GRN has a concentrated regulatory architecture with hubs of outgoing regulation; when d is large, regulatory activity is spread more uniformly throughout the network, and there are many more regulators (but typically fewer hubs). Pseudocode for the algorithm is in Table S1.

Choosing hyperparameters

To specify a full GRN model using a DAG produced from this algorithm, and to produce gene expression from it, we followed the same procedure as used for the PPM DAGs. In our investigation of the joint effects of modular network structure and regulatory hubs, we simulated 10,000 GRNs using the following scheme to sample parameters.

- Number of genes $n = 5000$
- Degree uniformity $\log d \sim \mathrm{Uniform}(\log(1), \log(30))$
- Number of groups $k \sim \mathrm{Uniform}(2, 100)$
- Modularity term $m \sim \mathrm{Uniform}(1/k, 1)$
- Expected regulators per gene $r \sim \mathrm{Uniform}(2, 10)$
- Strength of regulation $\gamma \sim \mathrm{Uniform}(0.2, 0.5)$

CellPress
OPEN ACCESS

- Expected fraction of activators $p^{+} \sim \text{Uniform}(0, 1)$

We used these same 10,000 GRNs when matching the cumulative distribution of h^2_{cis}/h^2 to that observed in real data.[7]

Expression variance decomposition

To further assess the genetic architecture of gene expression in example simulated GRNs, we used the same variance decomposition as in Equation 14—as a measure of the *trans*-effect B_{ji} of regulator j on gene i, we used the off-diagonal entries of the matrix L:

$$B_{ji}^2 = \frac{V_{\text{trans},j \to i}}{V_{\text{G},i}} = \frac{L_{ji}^2}{(L^\top L)_{ii}}, L = (I - G)^{-1} \quad \text{(Equation 15)}$$

The leading *trans*-effect for gene i (as in Figures 5B, 6E, and 6F) is the maximum effect over all regulators j: $\max_{j \neq i} B_{ji}^2$. To assess the contributions of *trans*-regulators at varied distances d from gene i in the network, we compute the all-pairs shortest path distances between all genes in the network, then sum contributions from regulators at distance d—if d_{ji} is the distance from gene j to gene i, then the fraction of variance due to regulators at distance d is:

$$\frac{V_{\text{trans},i,\text{dist. } d}}{V_{\text{G},i}} = \frac{\sum_{j, d_{ji} = d} L_{ji}^2}{(L^\top L)_{ii}} \quad \text{(Equation 16)}$$

as is shown in Figures 6, S17, and S18.

QUANTIFICATION AND STATISTICAL ANALYSIS

Real data comparison

To assess the similarity of simulated GRNs to experimental data, we compared the expected cumulative distribution of *cis*-acting expression variance ($V_{\text{cis}}/V_{\text{G}}$) in each synthetic GRN to the cumulative distribution of the fraction of estimated h^2_{cis}/h^2 for 5,902 genes with a *cis*-eQTL from whole blood expression data.[7] These data are described above. We computed Kolmogorov-Smirnov (K-S) test statistics as a non-parametric measure of distance between the distributions from the simulated GRNs and that of real data. To determine which features of the gene expression model and network generating parameters were associated with closer matches to data, we further examined the GRNs at the 2.5% tail of the distribution of K-S test statistics (i.e., 250 lowest values) against the remaining 97.5% of simulated GRNs (from both PPM and scale-free GRNs; Figures 5 and S12).

As independent replication, we repeated this analysis using the same set of simulated networks but comparing instead to the empirical distribution of the fraction of estimated h^2_{cis}/h^2 for 8,954 transcripts from adipose tissue (IFA) and for 5,979 transcripts from whole blood (IFB), using summary data from previous analyses of two Icelandic family studies.[4] These data are described above.

Cell Genomics, Volume *6*

Supplemental information

Regulatory network topology

and the genetic architecture of gene expression

Matthew Aguirre, Jeffrey P. Spence, Guy Sella, and Jonathan K. Pritchard

Supplemental Note

Methods S1: Modeling gene expression on a directed acyclic graph, related to **STAR Methods**.

In this section, we provide additional motivation for the statistical methods and derive some mathematical formulas used in our study.

We model gene expression with a linear structural equation model (SEM) given a set of causal regulatory relationships specified by a directed acyclic graph (DAG) with n genes. We assume that the structure of the DAG is given and the parameters of the SEM are fully specified, and consider relaxations of this assumption later.

To start, consider the expression of a single gene i: suppose the gene harbors q_i independent cis-eQTLs ($q_i > 0$) and is affected by r_i regulators ($r_i \geq 0$), which may or may not be independent. If we measure the expression y_i of this gene in a randomly chosen individual from population, we can write that

$$y_i = \underbrace{\sum_{k=1}^{q_i} x_{ki}\beta_{ki}}_{\text{cis}} + \underbrace{\sum_{j=1}^{r_i} y_j \gamma_{ji}}_{\text{trans}} + \underbrace{s_i}_{\text{noise}}$$

where above, β_{ki} and x_{ki} are respectively the effect of the k'th eQTL for gene i and an indicator variable for its genotype; γ_{ji} and y_j are the effect of the j'th regulator of gene i and its expression; and s_i is non-genetic noise with zero mean and variance σ_i^2, which we assume is uncorrelated across genes.

Throughout this work, we assume that (1) each regulator acts consistently as an activator or repressor and has the same magnitude of effect (i.e., $\gamma_{ji} = p_j\gamma$, with p_j denoting the sign for gene j), and that (2) the variance due to *cis*-acting eQTLs and transcriptional noise are the same for all genes (respectively 1, and σ^2), and that (3) all genotypes and noise terms are independent. The variance for gene i is then

$$Var(y_i) = \underbrace{1}_{\text{cis}} + \underbrace{\gamma^2 \left(\sum_{j=1}^{r_i} Var(y_j) + 2 \sum_{j=1}^{r_i} \sum_{j'=1}^{j-1} p_j p_{j'} Cov(y_j, y_{j'}) \right)}_{\text{trans}} + \underbrace{\sigma_i^2}_{\text{noise}}$$

Now consider the expression of all genes in the entire network: if G is the weighted adjacency matrix of the provided DAG for the GRN, with entries γ_{ji} where regulatory relationships exist and zero otherwise, then we can more compactly write

$$y = \underbrace{Bx}_{\text{cis}} + \underbrace{Gy}_{\text{trans}} + \underbrace{s}_{\text{noise}}$$

where B is the matrix of genetic effects from *cis*-eQTLs and x is a vector of their genotypes. We note that the above expression is valid since G is the matrix representation for DAG — this specifies a coherent set of equations for the expression, y_i, of each gene, and guarantees that the matrix $(I - G)$ is invertible. We can rearrange terms in the above to compute the variance of y:

$$\begin{aligned}
(I - G)y &= Bx + s \\
y &= (I - G)^{-1}(Bx + s) \\
Var(y) &= L^\top Var(Bx + s)L, \quad L = (I - G)^{-1} \\
&= L^\top (I + \sigma^2 I)L \\
&= (1 + \sigma^2)L^\top L
\end{aligned}$$

in which the marginal genetic variance of gene i can be read off the diagonal of $L^\top L$: $V_G(y_i) = (L^\top L)_{ii}$. Moreover, this matrix product implies a decomposition of variance across genes in the network: since $(L^\top L)_{ii} = \sum_{j=1}^n L_{ji}^2$ then the contribution of each gene j to the variance of gene i is

$$B_{ji}^2 = \frac{L_{ji}^2}{(L^\top L)_{ii}},$$

and the *cis*-acting fraction of genetic variance is correspondingly

$$\frac{V_{\text{cis},i}}{V_{\text{G},i}} = \frac{L_{ii}^2}{(L^\top L)_{ii}}.$$

Furthermore, the equations above show that the genetic and non-genetic covariances have similar forms:

$$\Sigma_G(y) = L^\top L$$
$$\Sigma_E(y) = \sigma^2 L^\top L.$$

This means that any non-zero genetic contribution to variance has a corresponding non-genetic term which is scaled by a factor of σ^2, and so the heritability for all genes in the network is constant: $h_i^2 = 1/(1+\sigma^2)$. This parity also means that decompositions of genetic variance across genes in the network will generally correspond to decompositions of heritability, as long as this scaling factor is consistent across genes; for clarity in the main text, however, we refer to our work with genetic variance as such when using simulations from this model.

Furthermore, the matrix $L = (I - G)^{-1}$ in the above expression is a total effects matrix for the structural equation model, which gives the marginal effect of the expression of gene j on the expression of gene i. More concretely, this equation corresponds to the Taylor expansion

$$L = (I - G)^{-1} = I + G + G^2 + G^3 + \dots$$

which enumerates all paths through the graph — moreover, since G is the adjacency matrix of a DAG, this is convergent since G is nilpotent (in particular, G^n is the zero matrix since G cannot have a path of length n). We use the squared entries of this matrix as the basis of our proxy for scaled *trans*-eQTL effect sizes. If *trans*-eQTL k for gene i has *cis*-effect β_{kj} on gene j, then the squared *trans*-effect is $\beta_{ki}^2 = (\beta_{kj} L_{ji})^2$. In practice, however, these effects are computed relative to the variance in expression of the focal gene (j for the *cis* effect, or i for the *trans* effect). Hence, to compare the relative scales of *cis* and *trans* effect sizes in this model, we use the proportion of variance of gene i explained by gene j,

$$B_{ji}^2 = \frac{L_{ji}^2}{(L^\top L)_{ii}}$$

as a proxy for *trans* effects on gene i from *cis*-eQTLs of gene j. This corresponds to the assumption that *cis* effects $\beta_{kj}, \beta_{k'i}$ (of eQTL k on gene j, and eQTL k' on gene i) are equal.

Supplementary Tables

Algorithm 1 Modular scale-free DAG

Require:

- n: Number of genes (nodes) in the network ($n \geq 3$).
- k: Number of groups in the network ($1 \leq k \leq n$).
- r: Expected number of regulators per gene ($r > 0$).
- m -or- w: Expected fraction of edges within groups ($0 \leq m \leq 1$; $w \geq 0$; $m = w/(w + k - 1)$)
- δ: Out-degree uniformity term ($\delta \geq 0$).

▷ *Temporarily set the top of the graph G to be a cycle on $\lfloor 2r \rfloor$ nodes.* ◁

$G \leftarrow \{(1 \to 2), (2 \to 3), \ldots, (\lfloor 2r \rfloor \to 1)\}$

▷ *Randomly assign each of the n nodes to one of k evenly sized groups.* ◁

group(i) $\leftarrow I_i, \quad I_i \in \{1, 2, \ldots, k\}$

▷ *First simulate the bottom section of the DAG, from genes $\lfloor 2r \rfloor + 1, \ldots, n$.* ◁

for $i \in \{\lfloor 2r \rfloor + 1, \ldots, n\}$ **do**

  ▷ *Pick a Poisson number of regulators for gene i (i.e., its in-degree):* ◁

  in-deg(i) $\sim$ Poisson(r)

  for $j \in \{1, \ldots,$ in-deg(i)$\}$ **do**

    ▷ *Sample the j'th regulator with replacement, weighted by group membership and out-degrees.* ◁

    $j \leftarrow j \in \{1, \ldots, i-1\}, \quad p_{ji} \propto (1 + (w-1)\mathbb{1}(I_i = I_j)) \times$ (out-deg(j) $+ \delta$)

    ▷ *Add the edge (j, i), allowing duplicate edges, to the graph* ◁

    $G \leftarrow (j \to i)$

▷ *Then replace the temporary edges at the top of the DAG with random structure.* ◁

for $i \in \{1, \ldots, \lfloor 2r \rfloor\}$ **do**

  for $j \in \{1, \ldots, i-1\}$ **do**

    ▷ *These edges exist independently with equal probability.* ◁

    $G \leftarrow (j \to i), \quad$ with probability $p_{ji} = 2r/(n-1)$

Table S1: Algorithm to generate modular scale-free directed acyclic graphs, related to **Fig. 2**.

Supplementary Figures

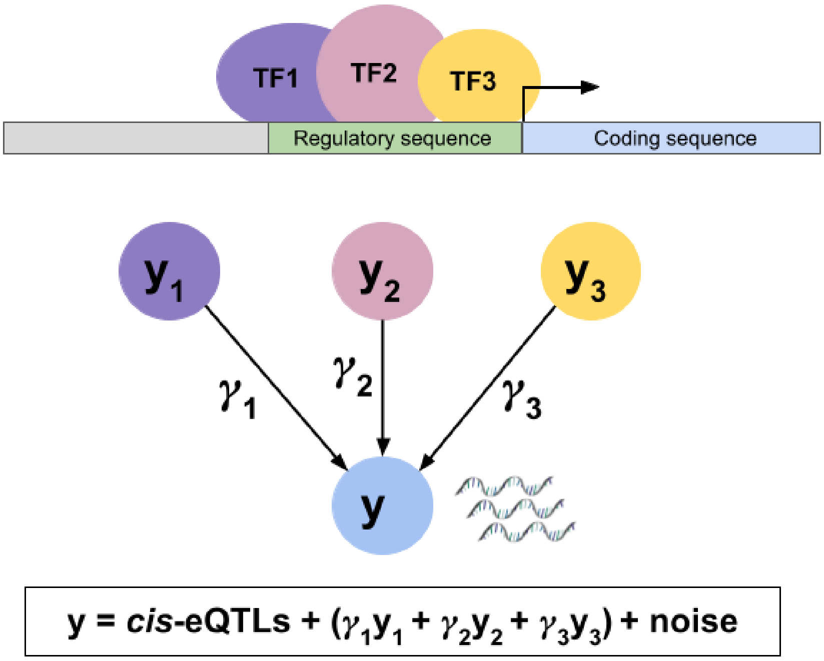

Figure S1: Biological interpretation of linear structural equation model, related to **Fig. 1**. Nodes in the gene regulatory network represent genes. Edges represent regulatory interactions, e.g., between transcription factors and a target gene. Each interaction has strength γ, which corresponds to the (linear) effect of one unit change in expression of the regulator (e.g., y_1) on the expression of the target gene (here, y). Note that the equation in this figure abstract away the effect sizes of *cis*-eQTLs and the nature of endogenous transcriptional noise for the focal gene. Its *trans*-eQTL effects are likewise implicit in the expression in terms of its regulators — they are *cis*-eQTLs for the expression of y_1, y_2, and y_3.

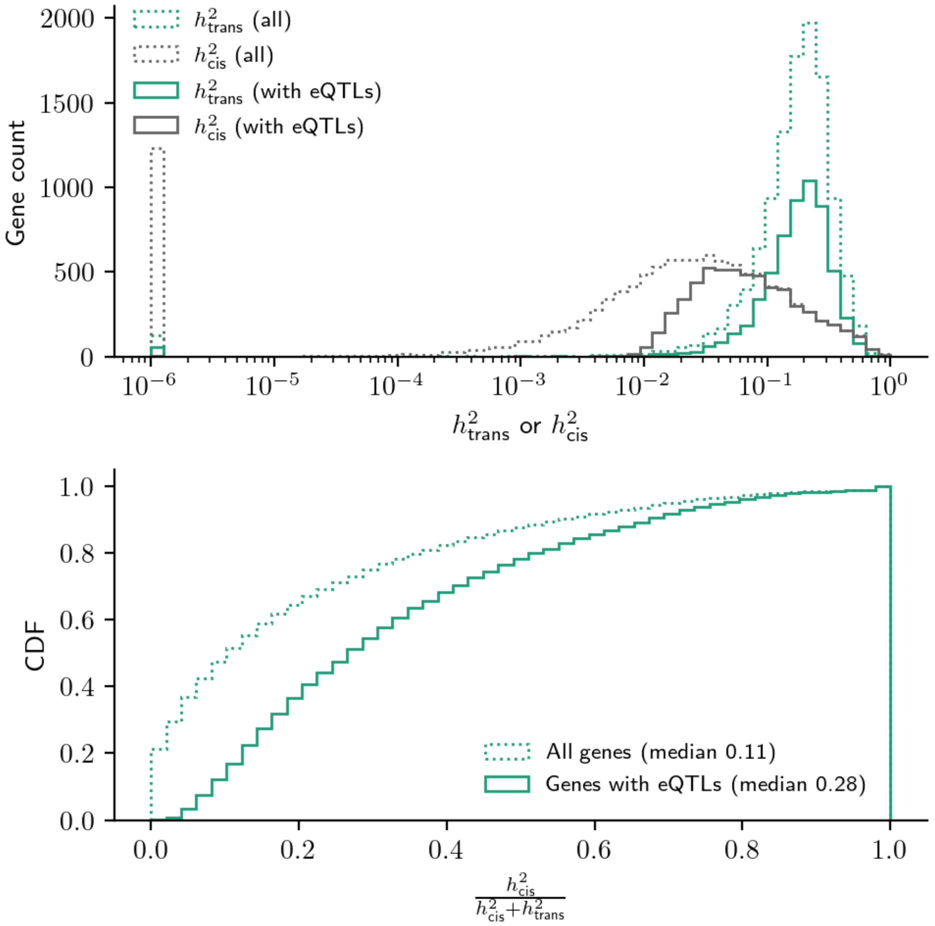

Figure S2: Distribution of *cis* and *trans* heritability for genes with and without eQTLs, related to **Fig. 1**. Data from (Ouwens *et. al.*, 2020)[1] **(A)** Distribution of h^2_{cis} and h^2_{trans} for all $11{,}353$ genes with available data from the study (dotted lines) or for all $5{,}902$ genes in the analysis subset from **Fig. 1**, with a detected eQTL (solid lines). **(B)** Distribution of *cis*-heritability fraction from these same gene sets.

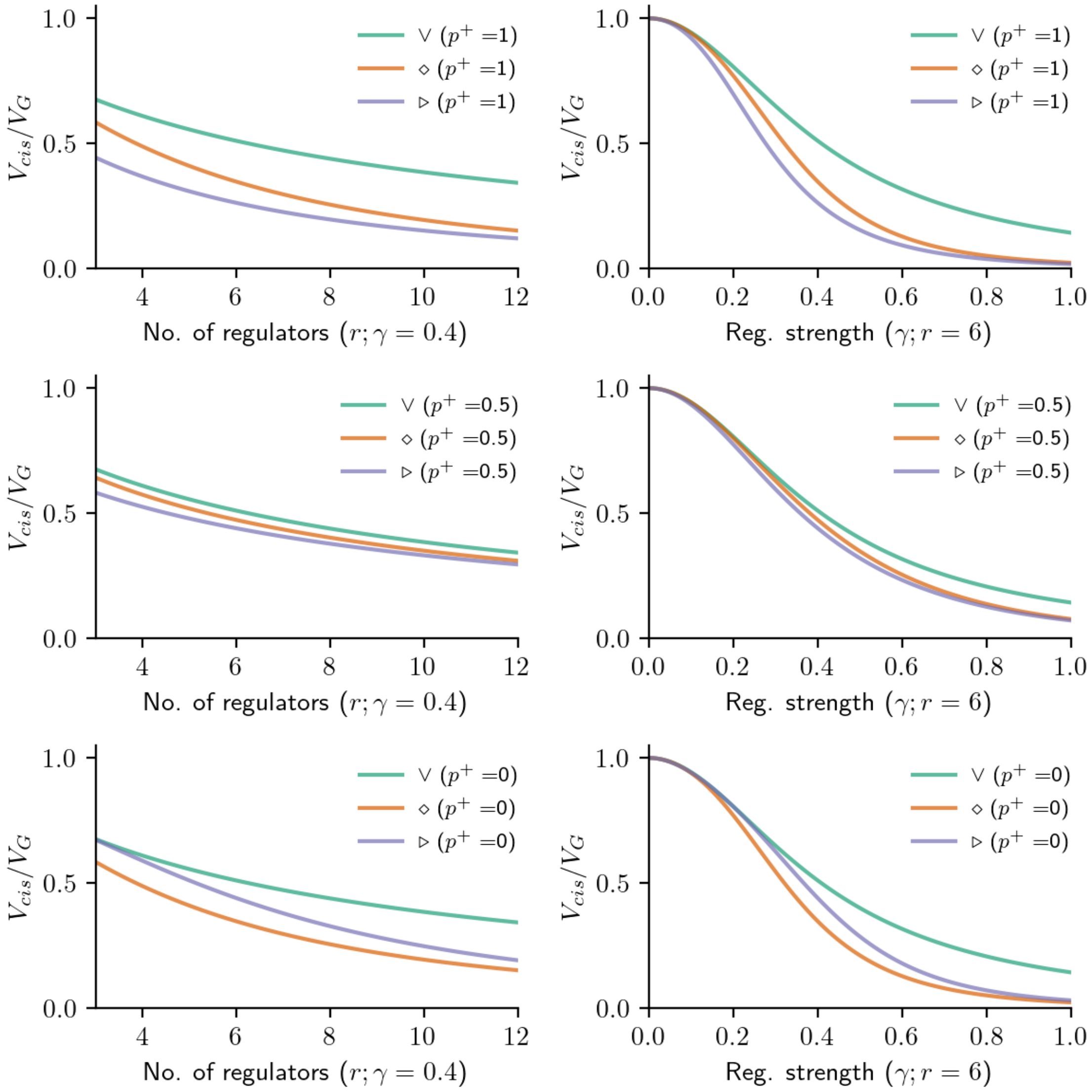

Figure S3: Effect of motifs with different fractions of activators, related to **Fig. 2**. Representative effects of the number of regulators (r; left panels) and the strength of regulation (γ; right panels) on the distribution of *cis*-acting variance for the three motifs in **Fig. 2**. The underlying mathematical equations for vee, triangle, and diamond motifs are as in the main text and corresponding figure — here, the expressions are also stratified by representative fractions of activators p^+ (the panels in the top row are exactly as in **Fig. 2**).

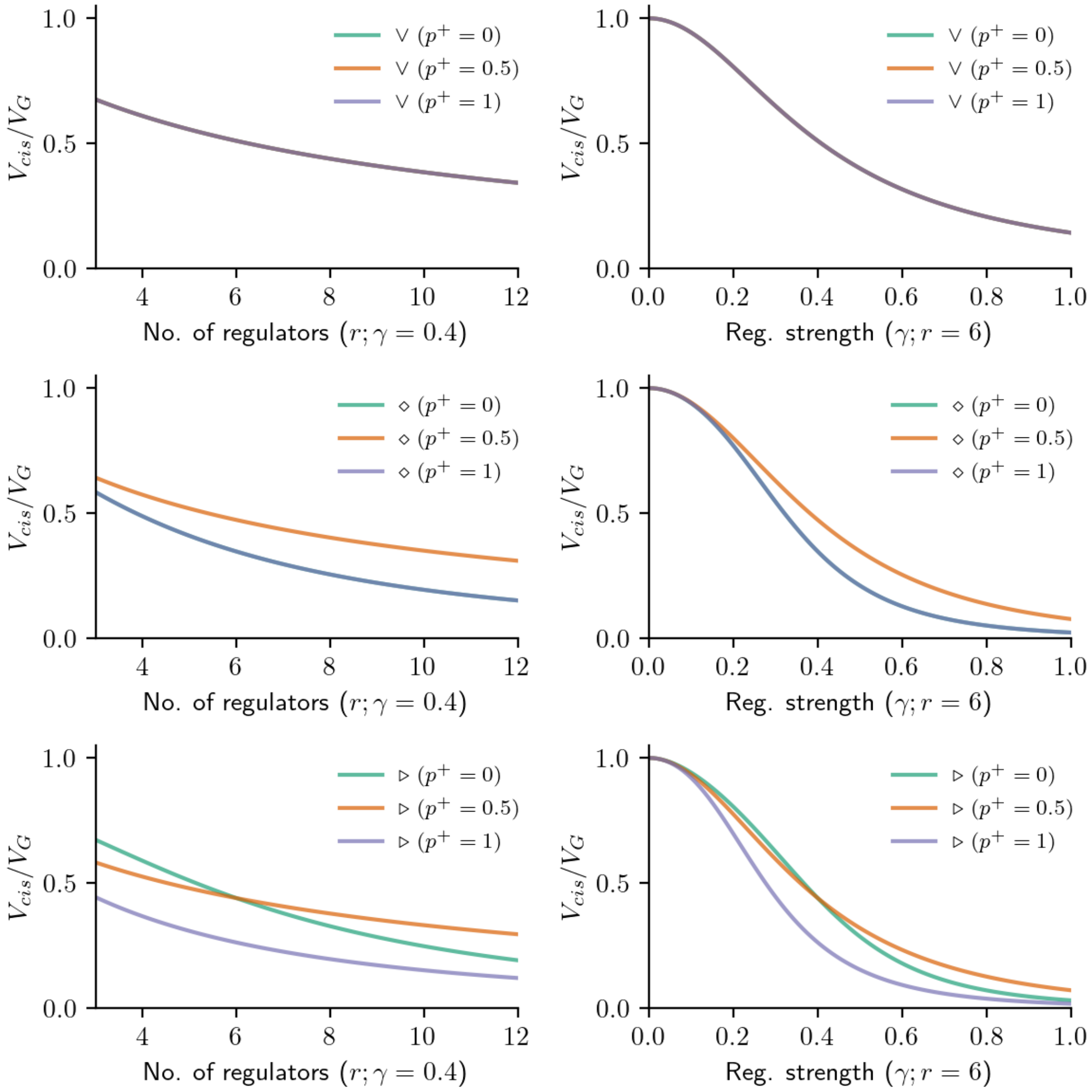

Figure S4: Effect of regulatory sign on different motifs, related to **Fig. 2**. Representative effects of the number of regulators (r; left panels) and the strength of regulation (γ; right panels) on the distribution of *cis*-acting variance for the three motifs in **Fig. 2**. The lines in this plot are the same as in **Fig. S3**, but subpanels here correspond to the separate motifs (vee, diamond, and triangle, in each row) rather than distinct values for the fraction of activators (p^+).

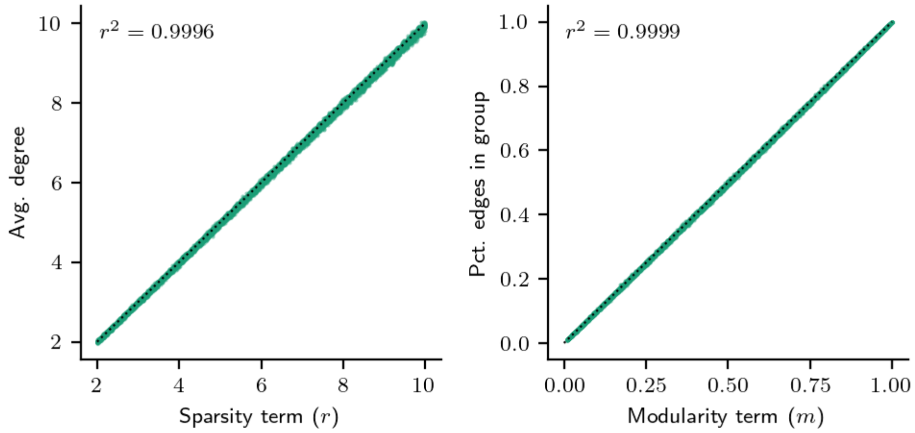

Figure S5: Parameters of the planted partition model (PPM) control key graph properties, related to **Fig. 3**. Values for the sparsity term (r; left panel) and modularity term (m; right panel) in the $10{,}000$ synthetic GRNs generated using the planted partition model (see **Methods**). The sparsity term r is extremely correlated with the average degree in the resulting GRN, and the modularity term m is extremely correlated with the resulting fraction of edges in the GRN that are drawn between genes in the same group (rather than genes in separate groups).

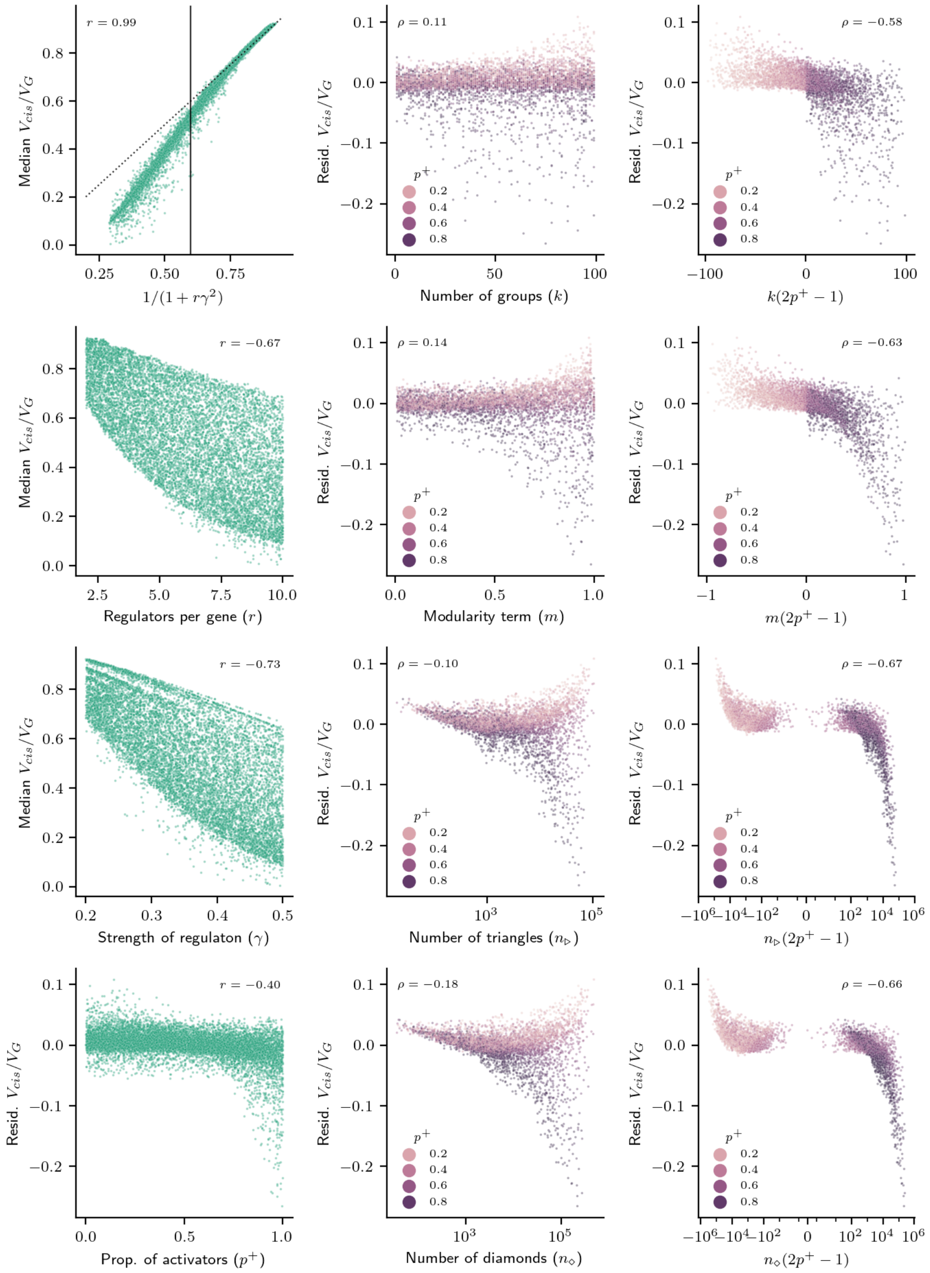

Figure S6: Parameters of the planted partition model (PPM) affect the distribution of heritability, related to **Fig. 3**. Relationships between properties of GRNs produced using the PPM and summaries of the median fraction of *cis*-acting expression variance in the corresponding $10{,}000$ GRNs. Subpanels are annotated by the Pearson (r) or Spearman (ρ) correlation between the quantities shown on the axes — median $V_{\text{cis}}/V_{\text{G}}$ denotes the untransformed value, while "Resid. $V_{\text{cis}}/V_{\text{G}}$" denotes the residual having regressed out direct effects as $1/(1 + r\gamma^2)$. Networks in the middle and right columns are shown stratified by the fraction of activators p^+ and are subsetted to GRNs with lower expected contributions from direct effects $(1/(1 + r\gamma^2) < 0.6)$, denoted by a solid vertical line in the top left panel (the dashed line is $y = x$); these are also the networks shown in **Fig. 3**.

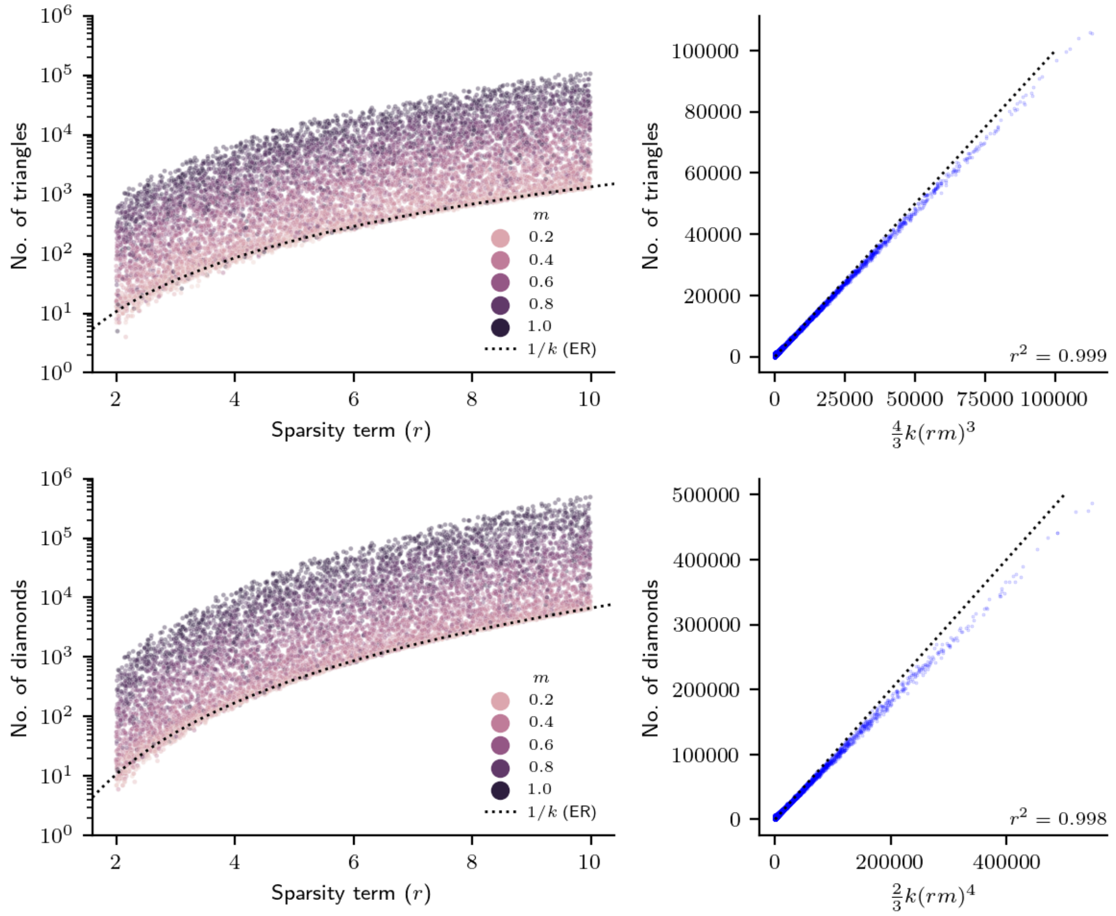

Figure S7: Parameters of the planted partition model (PPM) affect the number of motifs in the network, related to **Fig. 3**. Relationships between the sparsity (r) and modularity (m) parameters of the PPM and the resulting number of triangle and diamond motifs (defined as diagrammed in **Fig. 2**) in $10{,}000$ PPM GRNs. In both plots, each point is a network — the left panels show the interaction between r and m (note that $m = 1/k$ is the minimum value simulated in the study, and corresponds to the binomial graph); the right panels show the relationship between motif counts and the leading term of a mathematical approximation to the expected number of motifs.

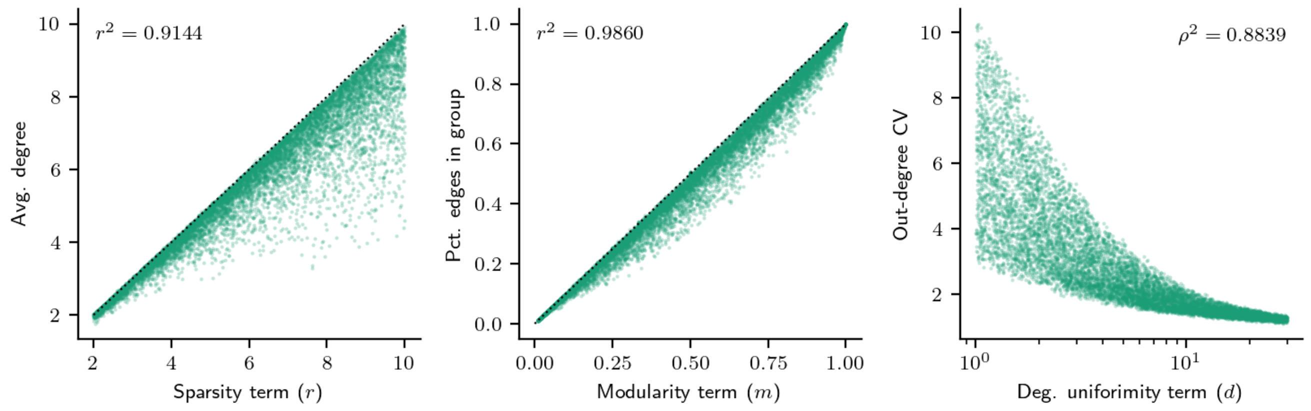

Figure S8: Parameters of the modular scale-free graph model control key graph properties, related to **Fig. 4**. Values for the sparsity term (r; left panel), modularity term (m; center panel), and degree uniformity term (d; right panel) in the $10{,}000$ synthetic GRNs generated using the modular scale-free graph generating algorithm (see **Methods**). The sparsity term r is strongly correlated with the average degree in the GRN. The modularity term m is strongly correlated with the fraction of edges in the GRN that are drawn between genes in the same group (rather than genes in separate groups). The degree uniformity term d is strongly correlated with the coefficient of variation (CV) of the out-degree distribution — here, CV is the standard deviation of the degree distribution over its mean, and a larger CV corresponds to a more dispersed distribution.

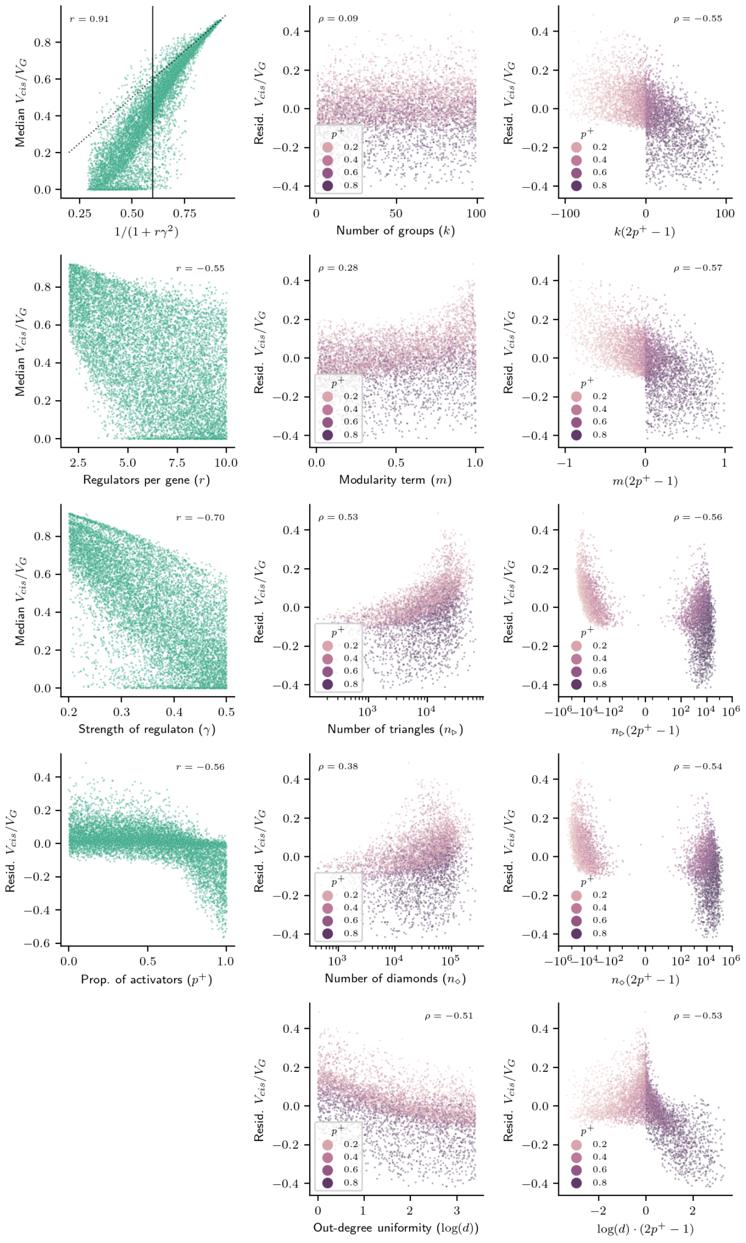

Figure S9: Parameters of the modular scale-free graph model affect the distribution of heritability, related to **Fig. 4**. Relationships between properties of GRNs produced using the modular scale-free graph model and summaries of the median fraction of *cis*-acting expression variance in the corresponding $10{,}000$ GRNs. Subpanels are annotated by the Pearson (r) or Spearman (ρ) correlation between the quantities shown on the axes — median $V_{\text{cis}}/V_{\text{G}}$ denotes the untransformed value, while "Resid. $V_{\text{cis}}/V_{\text{G}}$" denotes the residual variance after regressing out direct effects as $1/(1 + r\gamma^2)$. Networks in the middle and right columns are shown stratified by the fraction of activators p^+ and are subset to GRNs with lower expected contributions from direct effects ($1/(1 + r\gamma^2) < 0.6$, denoted by a solid vertical line in the top left panel (the dashed line is $y = x$); these are also the networks shown in **Fig. 4**.

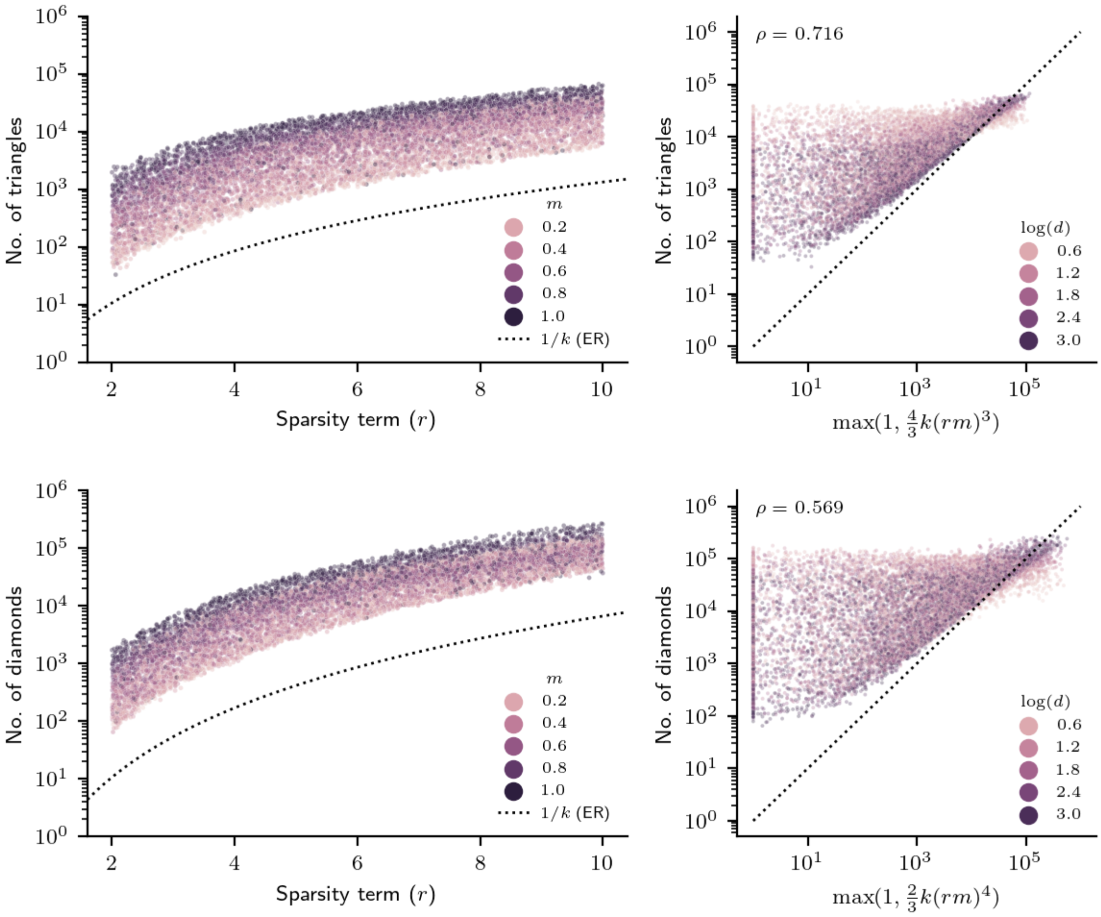

Figure S10: Parameters of the modular scale-free graph model affect the number of motifs in the network, related to **Fig. 4**. Relationships between the sparsity (r) and modularity (m) parameters of the modular scale-free graph model and the resulting number of triangle and diamond motifs (defined as diagrammed in **Fig. 2**) in $10{,}000$ GRNs. In both plots, each point is a network — the left panels show the interaction between r and m (note that $m = 1/k$ is the minimum value simulated in the study, and corresponds to the value which yields the binomial graph in the PPM); the right panels show the relationship between motif counts and the leading term of a mathematical approximation to the expected number of motifs to the PPM, and that smaller values of d (which introduce hubs into the graph) drive deviations from this expected value.

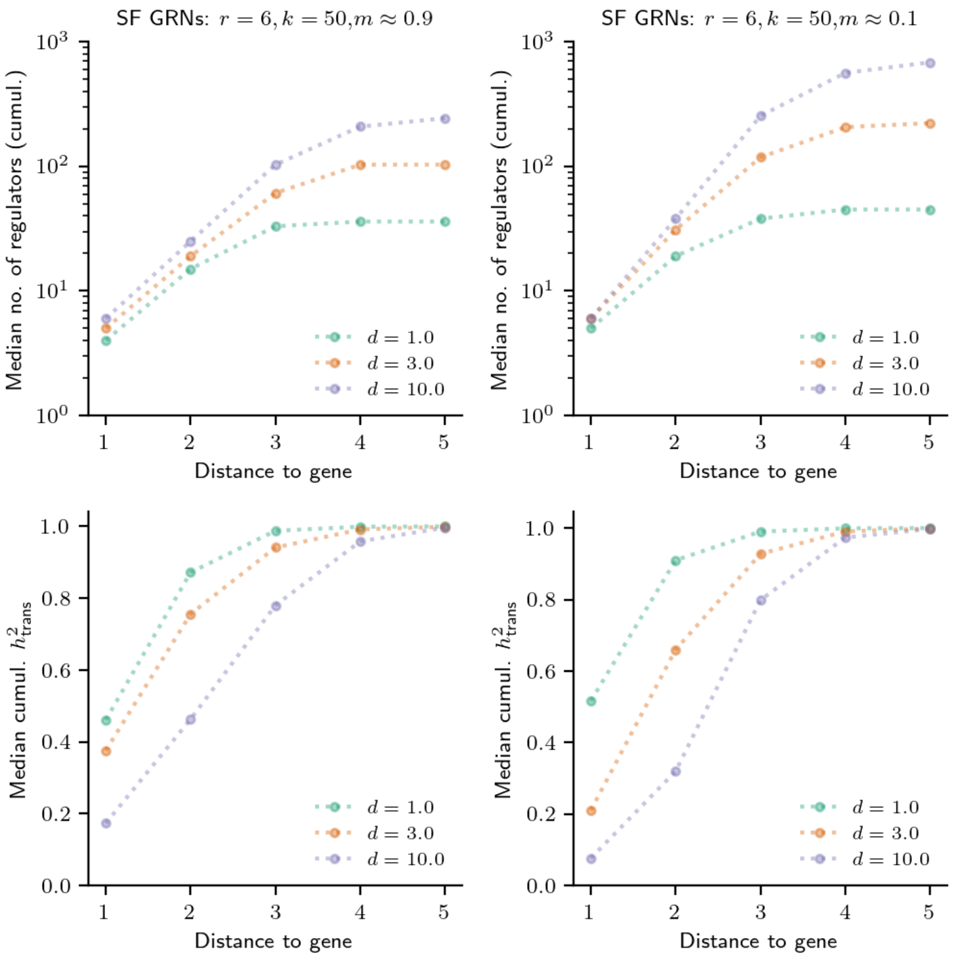

Figure S11: Degree uniformity alters the distribution of path lengths in the network, related to **Fig. 4**. Median number of regulators (top panels) and cumulative *trans*-acting expression variance (bottom panels) in six example modular scale-free GRNs, generated with identical terms for sparsity ($r = 6$), number of groups ($k = 50$), regulatory strength ($\gamma = 0.5$), and composition ($p^+ = 0.7$), but varying modularity ($m \approx 0.9$ or $m \approx 0.1$) or degree dispersion ($d = 1$, 3, or 10). Medians are over genes in the middle of the topological sorted order of the DAG (indexes 2000 to 3000). GRNs with regulatory hubs (smaller values of d) have fewer distant *trans*-regulators and *trans*-acting heritability is thus typically closer to a given gene in the network.

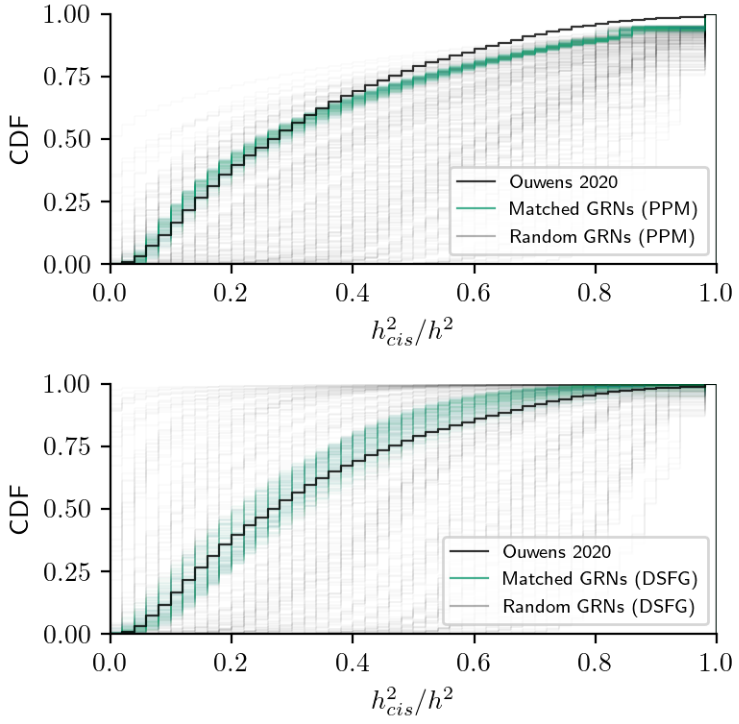

Figure S12: Distribution of heritability in synthetic GRNs and for whole-blood gene expression, related to **Fig. 5**. Distribution of the fraction of *cis*-acting heritability (h^2_{cis}/h^2) from real data (Ouwens *et. al.* 2020)[1] and from synthetic GRNs generated using the planted partition model (PPM; top panel) or the directed scale-free graph generating algorithm (SF; bottom panel). In both plots, teal lines are the 250 GRNs closest to the distribution from data (lowest K-S test statistics; see **Methods**), and grey lines are a random sample of 250 GRNs from the other $9{,}750$ simulated GRNs.

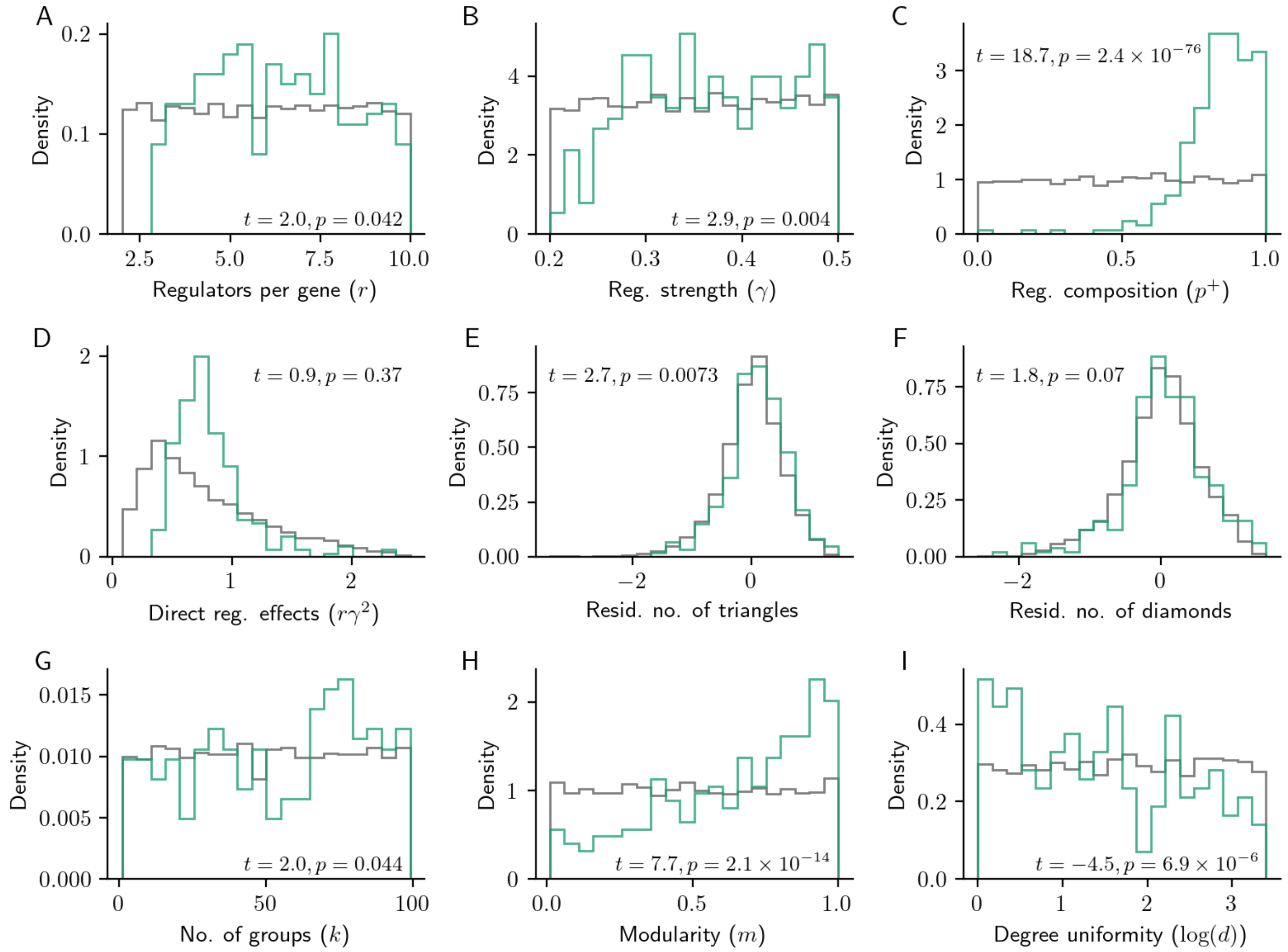

Figure S13: Properties of synthetic GRNs that best resemble real data, related to **Fig. 5**. Additional properties of the 250 GRNs that were best matched to the observed distribution of *cis*-acting heritability fractions (shown in teal, as in **Fig. 5**). Each panel is annotated with results from a two-sample t-test for a difference in mean with the remaining $9{,}750$ GRNs (shown in grey).

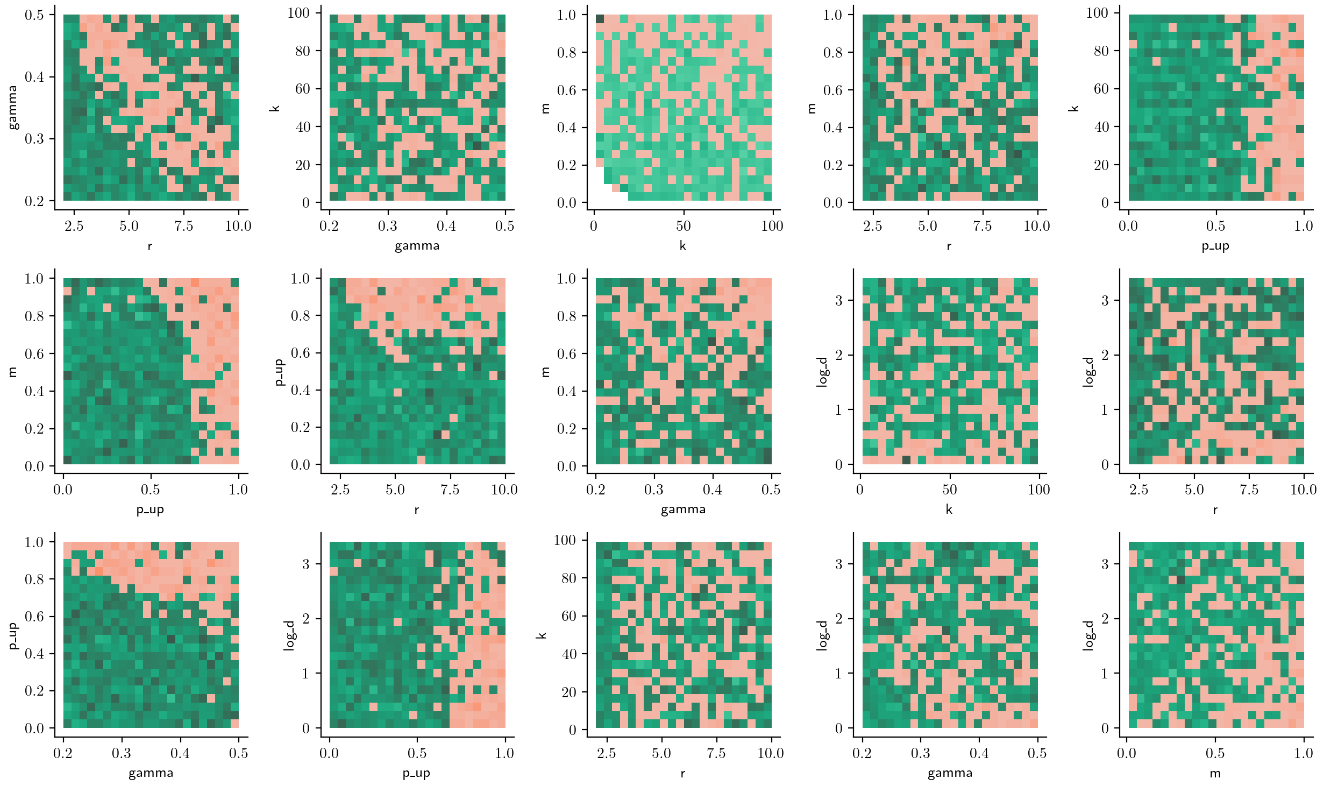

Figure S14: Interactions between properties of synthetic GRNs that best resemble real expression heritability data, related to **Fig. 5**. Pairwise interactions between properties of the 250 GRNs that were best matched to the observed distribution of *cis*-acting heritability fractions. In each subpanel, red denotes an enrichment of matched GRNs in and green denotes depletion.

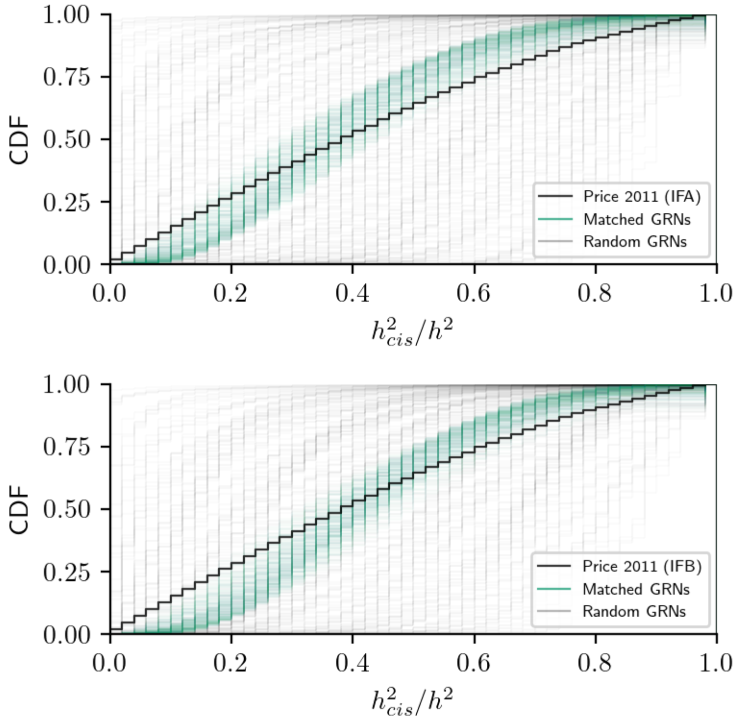

Figure S15: Distribution of heritability in synthetic GRNs and for whole-blood and adipose tissue gene expression, related to **Fig. 5**. Distribution of the fraction of *cis*-acting heritability (h^2_{cis}/h^2) from real data (Price *et. al.* 2011[2]; top panel [IFA] is adipose tissue, and bottom panel [IFB] is whole blood) and from synthetic GRNs generated using the directed scale-free graph generating algorithm. In both plots, teal lines are the 250 GRNs closest to the distribution from data (lowest K-S test statistics; see **Methods**), and grey lines are a random sample of 250 GRNs from the other $9{,}750$ simulated GRNs.

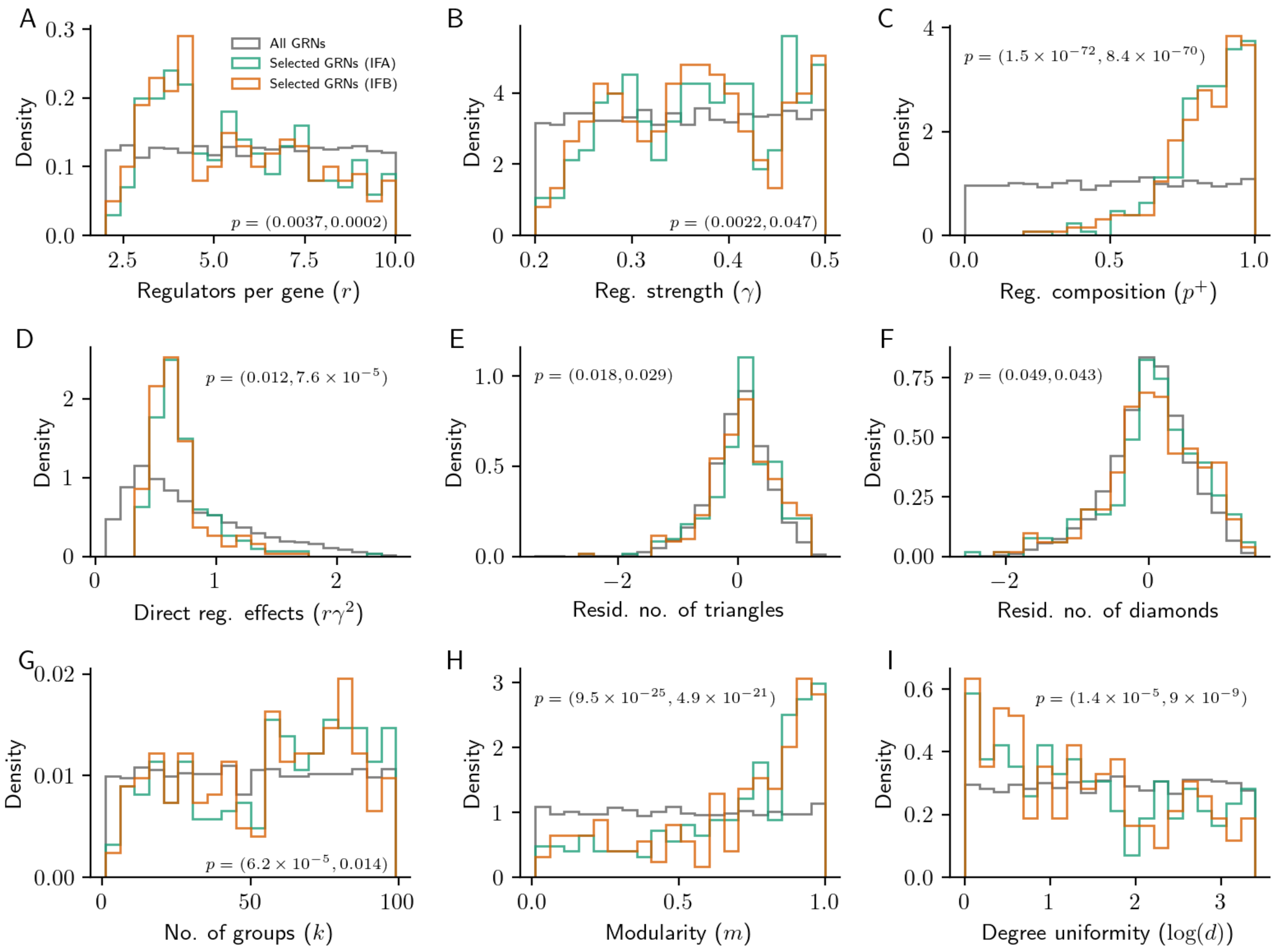

Figure S16: Properties of synthetic GRNs that best resemble replication data, related to **Fig. 5**. Additional properties of the 250 GRNs that were best matched to the observed distribution of *cis*-acting heritability fractions from the replication data (Price *et. al.* 2011[2]), as in **Fig. 5**. GRNs matched to results from adipose (IFA) are shown in teal, GRNs matched to results from whole blood are shown in orange, and unmatched GRNs are shown in grey. Each panel is annotated with results from a two-sample *t*-test for a difference in mean with the remaining $9{,}750$ GRNs (shown in grey).

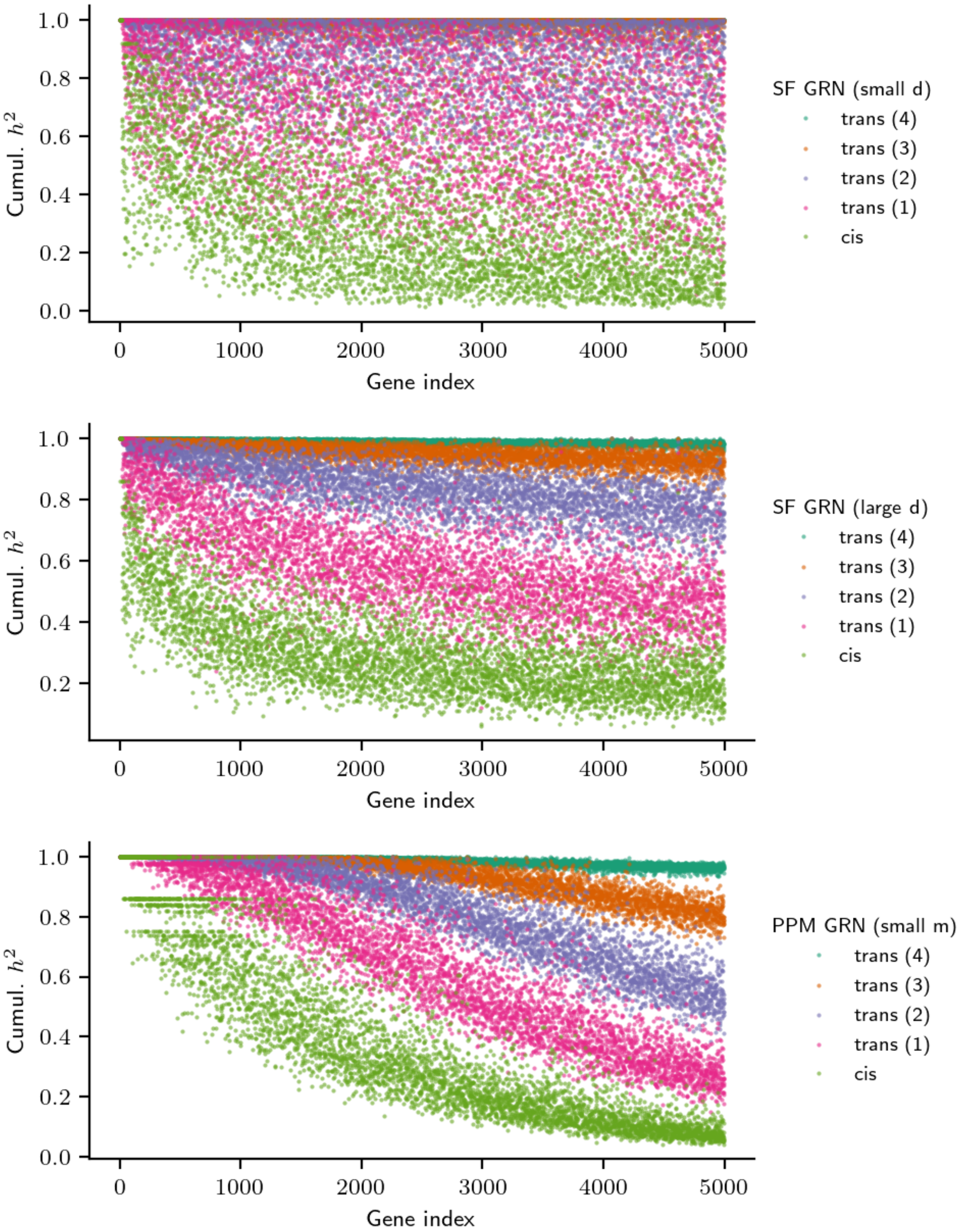

Figure S17: Gene-level heritability distribution over network distances in three example GRNs, related to **Fig. 6**. Cumulative heritability in *cis* and in *trans*, stratified by distance, for all genes in the three example networks from **Fig. 6**.

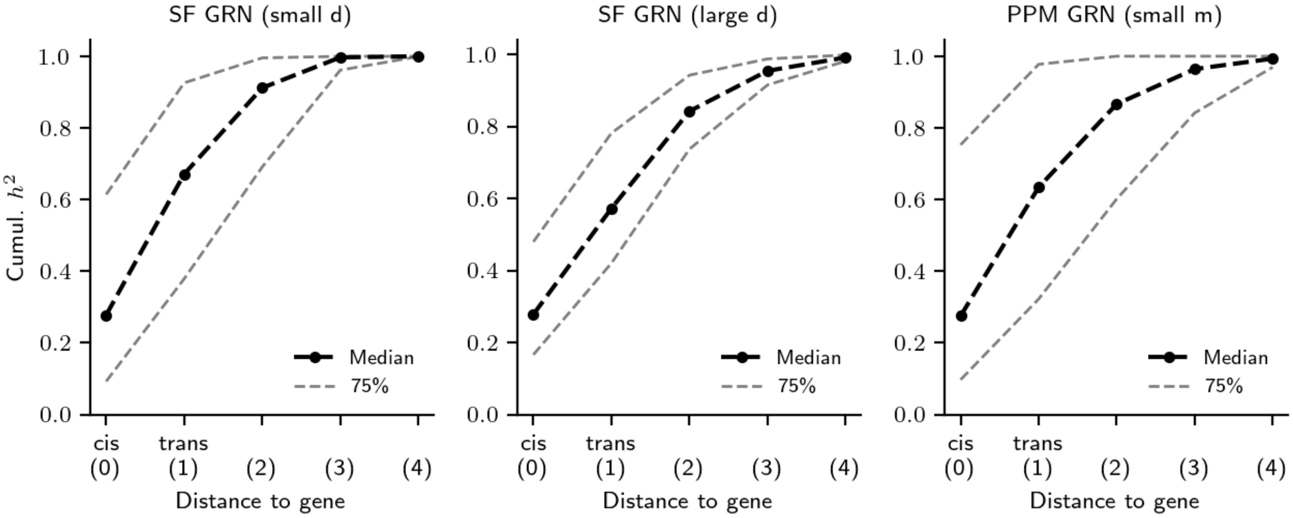

Figure S18: Cumulative heritability distribution over network distances in three example GRNs, related to **Fig. 6**. Cumulative heritability in *cis* and in *trans*, stratified by distance, for the median gene (or quartiles) in the three example networks from **Fig. 6B** — note that the medians and quantiles are computed separately for each tick in the x-axis (i.e., the gene with the median *cis*-acting heritability fraction may not be the gene with the median one-hop *trans*-acting heritability fraction).

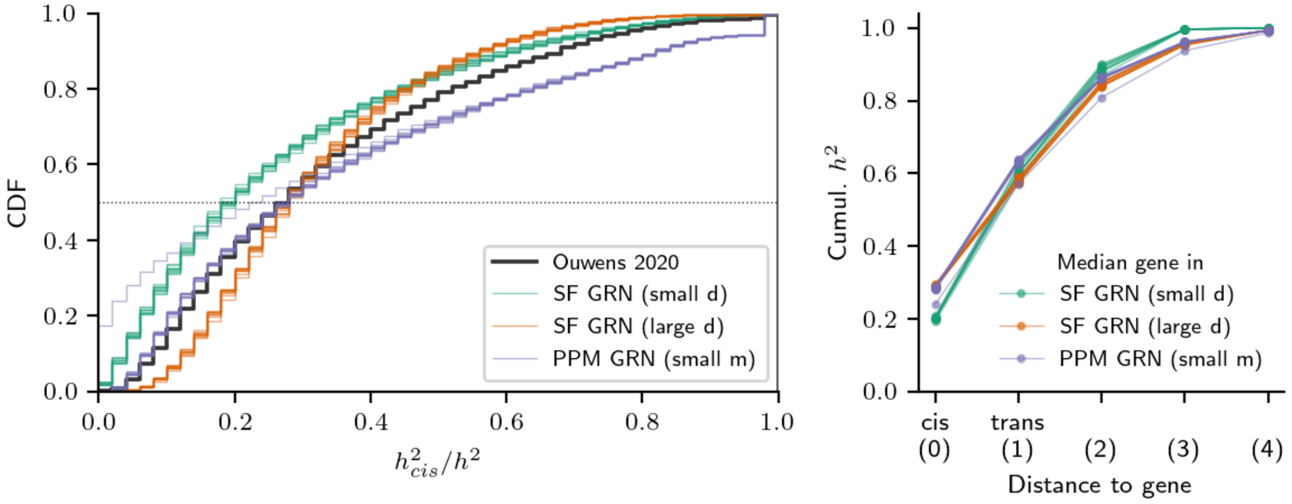

Figure S19: Median *cis*- and cumulative heritability over distances in several example GRNs, related to **Fig. 6**. Replication of results in **Fig. 6A-B**. Thirty GRNs in this figure have the same edges as the three example GRNs in **Fig. 6**, but have resampled parameters of the gene expression model, taking $|\gamma|_i \sim_{iid} \mathcal{N}(\gamma, \gamma/5)$ for each gene i (10 resampled GRNs for each in the main Figure). **(A)** Cumulative distribution of the fraction of *cis*-acting expression variance in each resampled GRN, plotted against the distribution from real data (Ouwens et. al., 2020)[1] **(B)** Median cumulative heritability as a function of network distance in each resampled GRN. Median is over genes in the network; cis-effects are at distance 0, trans-effects from direct regulators are at distance 1, etc.

References

1. Ouwens, K.G., Jansen, R., Nivard, M.G., van Dongen, J., Frieser, M.J., Hottenga, J.J., Arindrarto, W., Claringbould, A., van Iterson, M., Mei, H., Franke, L., Heijmans, B.T., A. C. 't Hoen, P., van Meurs, J., Brooks, A.I., Penninx, B.W.J.H., and Boomsma, D.I. (2020). A characterization of cis- and trans-heritability of RNA-Seq-based gene expression. Eur J Hum Genet *28*, 253–263. URL: https://www.nature.com/articles/s41431-019-0511-5. doi: 10.1038/s41431-019-0511-5. Publisher: Nature Publishing Group.

2. Price, A.L., Helgason, A., Thorleifsson, G., McCarroll, S.A., Kong, A., and Stefansson, K. (2011). Single-Tissue and Cross-Tissue Heritability of Gene Expression Via Identity-by-Descent in Related or Unrelated Individuals. PLOS Genetics *7*, e1001317. URL: https://journals.plos.org/plosgenetics/article?id=10.1371/journal.pgen.1001317. doi: 10.1371/journal.pgen.1001317. Publisher: Public Library of Science.
